# Ecological harm and criminal thresholds: A pilot legal-ecological coding framework for environmental crime judgments

**DOI:** 10.12688/openreseurope.24170.1

**Published:** 2026-06-17

**Authors:** Esteban Morelle-Hungría

**Affiliations:** 1 Public Law, University Jaume I Faculty of Law and Economics, Castellón de la Plana, Valencian Community, 12071, Spain

**Keywords:** empirical legal studies, environmental crime, ecological harm, criminal thresholds, judicial decisions, legal coding, composite index

## Abstract

Environmental criminal law frequently relies on open-textured threshold concepts such as “substantial damage,” “serious harm,” and “serious risk” to distinguish administrative unlawfulness from criminally relevant conduct. Yet courts often apply these thresholds without a reproducible framework for structuring ecological seriousness across cases. This article introduces LEDAT ^®^ (Legal-Ecological Damage Assessment Tool), a structured coding framework that combines legal and ecological variables into a Material Environmental Criminal Damage Index (IDPAm), while distinguishing material seriousness from procedural and evidential enforceability. The framework is piloted on 36 Spanish first-instance environmental crime judgments issued between 2020 and 2025. Cases were independently coded by two trained researchers using a shared coding manual and reconciled after comparison. IDPAm values ranged from 0.7 to 9.3. Most cases fell within predominantly administrative or intermediate grey-area bands, while four reached high material criminal significance. Convictions showed higher descriptive IDPAm values than acquittals, although distributions overlapped substantially and eleven of twenty convictions were conformity-based. Sensitivity checks indicate high ordinal stability under alternative legal/ecological weights, while trigger removal affects band classification. The article makes no predictive, causal, or inferential claims. Its contribution is methodological: LEDAT offers a transparent, contestable, and reproducible framework for empirical analysis of environmental criminal thresholds.

## 1. Introduction

Environmental criminal ^1^ law in the European Union continues to rely on open-textured threshold concepts such as “substantial damage,” “serious harm,” and “serious risk” to distinguish administrative unlawfulness from criminally relevant conduct. These concepts perform an essential legal function: they mark the point at which regulatory non-compliance becomes sufficiently serious to justify criminal intervention. Yet they also create a recurring problem for empirical legal analysis. Although Directive (EU) 2024/1203 significantly strengthens the European framework by expanding offences, sanctions, and enforcement obligations, it does not provide an operational methodology for structuring the assessment of ecological seriousness across cases and jurisdictions. Legally decisive threshold concepts therefore remain methodologically under-specified, generating interpretative variability in the treatment of materially similar cases ( Fuentes Osorio, 2019, 2021).

This problem is not merely doctrinal. It is also empirical and evidential. Environmental crime poses particular challenges for criminal law because the harms involved are frequently cumulative, multidimensional, and difficult to delimit within conventional legal categories. Such harms may affect individuals, communities, species, and ecosystems simultaneously, often extending beyond immediate or localised degradation and producing broader forms of social harm that exceed traditional models of individualised victimisation ( Council of the European Union, 2023; Ruiz Arias, 2021). At the same time, environmental offences commonly depend on complex scientific evidence, since ecological systems are dynamic, adaptive, and rarely susceptible to simple linear attribution. The evidential difficulties associated with ecological harm are further intensified by institutional limitations in specialist expertise and investigative capacity ( Baucells, 2024).

These challenges arise within a broader context of ecological crisis and legal transformation. European environmental policy increasingly operates under conditions of escalating ecological pressure and political tension regarding the scope and intensity of environmental protection ( Fajardo del Castillo, 2023). From the ecocentric perspective adopted in this article, the relevant protected legal interest cannot be reduced to a merely administrative conception of “the environment”; rather, the ecosystem itself must be understood as a legal interest capable of autonomous criminal-law relevance ( Morelle-Hungría, 2024). This perspective is especially relevant following Directive (EU) 2024/1203, which replaces Directive 2008/99/EC and reinforces the role of criminal law within environmental governance. The recast Directive expands the catalogue of offences, strengthens sanctions for both natural and legal persons, and obliges Member States to improve training, coordination, resources, and enforcement capacity. At the same time, however, it continues to rely on evaluative threshold expressions—such as “substantial damage,” “widespread damage,” “irreversible or long-lasting damage,” and “non-negligible quantities”—whose practical application still depends on national interpretation, expert evidence, and judicial assessment.

The Directive is therefore relevant not only as a legal instrument, but also as a source of a measurement problem. First, it confirms that environmental criminal liability cannot be reduced to the formal breach of administrative standards alone, but requires an additional assessment of seriousness. Secondly, although the Directive identifies relevant assessment factors—including the baseline condition of the affected environment, the duration and reversibility of the damage, its extent, and the exceedance of regulatory thresholds—it does not itself provide a reproducible coding framework capable of organising those criteria within prosecutorial, expert, or judicial practice. Judicial discretion consequently remains unavoidable. Such discretion is not objectionable in itself; comparative scholarship has warned against excessively rigid decision-guidance models that could unduly constrain judicial autonomy ( Poppleton et al., 2021). The difficulty arises when the assessment of ecological seriousness remains implicit, fragmented, and methodologically opaque.

The Spanish legal system provides a useful empirical setting in which to examine this broader problem. Under Articles 325–327 of the Spanish Criminal Code, courts must frequently determine whether an unlawful emission, discharge, extraction, or ecological disturbance remains within the sphere of administrative unlawfulness or instead reaches the additional gravity required for criminal punishment. In practice, this determination often depends on technical assessments concerning ecological impact, risk, duration, reversibility, ecosystem sensitivity, pollutant concentration, or protected species and habitats. Spain is therefore not treated here as an isolated doctrinal case study, but as a jurisdictional context in which a general empirical legal problem can be observed: how courts reason about environmental criminal thresholds when the underlying harm is ecologically complex and evidentially mediated.

This article addresses that problem by proposing LEDAT ^®^ (Legal-Ecological Damage Assessment Tool), a structured legal-ecological coding framework designed to organise the assessment of material environmental criminal harm in a transparent, traceable, and contestable manner. LEDAT combines a Legal Component and an Ecological Component into a synthetic indicator—the Material Environmental Criminal Damage Index (IDPAm)—while distinguishing that substantive assessment from a separate Enforceability module (IDPAe), which concerns procedural and evidential viability. This distinction is central to the model’s logic. Materially serious ecological harm may fail procedurally, just as evidential sufficiency does not itself determine substantive ecological gravity. The framework therefore separates the empirical coding of material seriousness from the procedural question of whether a criminal case can be successfully sustained.

The architecture of LEDAT is intentionally restrained. The weighting assigned to the Legal Component (Wj = 0.4) and the Ecological Component (We = 0.6) reflects the fact that the index is designed primarily to structure material ecological seriousness while preserving a normative legal filter so that criminal relevance is not reduced to ecological measurement alone. Likewise, the trigger mechanism is capped at a maximum of +2 in order to capture qualified qualitative escalations—such as critically endangered species, maximum-protection ecosystems, or grave risks to human health—without allowing those factors to overwhelm the base matrix or generate disproportionate inflationary effects. LEDAT is therefore not conceived as an automated decision-making system, a sentencing grid, or a substitute for judicial reasoning. Its purpose is methodological: to make explicit, structured, and contestable the factors that already underlie threshold reasoning in environmental criminal law.

The article does not claim definitive validation of the model. Instead, it presents a pilot empirical application of LEDAT to a sample of 36 first-instance Spanish environmental crime judgments issued between 2020 and 2025 by Criminal Courts and Provincial Courts acting as trial courts according to the applicable procedural rules. The cases were independently coded by two trained junior researchers using a shared coding manual and an independent parallel coding protocol, in which each coder worked separately and without access to the other coder’s classifications before reconciliation. The empirical objective is exploratory: to assess the initial coherence, usability, and descriptive potential of the methodology in real case material.

The article makes three contributions to empirical legal studies. First, it reframes environmental criminal thresholds as a problem of legal-ecological coding and measurement, rather than solely as a problem of doctrinal interpretation. Secondly, it introduces a transparent and reproducible framework for organising heterogeneous legal and ecological variables in environmental crime judgments. Thirdly, it provides a pilot empirical application showing how structured coding may improve the comparability and contestability of threshold assessment without displacing judicial discretion. More broadly, the article argues that structured legal-ecological methodologies may contribute to greater transparency, consistency, and comparability in the enforcement of environmental criminal law within the post-2024 European framework, provided that their use remains non-binding, methodologically transparent, and fully compatible with the constitutional safeguards governing criminal adjudication.

## 2. The problem of “substantial harm”

The central difficulty addressed in this section is that “substantial harm” operates as a threshold concept whose legal function is indispensable but whose practical application remains unstable. It marks the boundary between administrative illegality and criminal wrongdoing, but does so through open-textured evaluative language that is difficult to apply consistently across cases. For empirical legal analysis, this creates a measurement problem: courts, prosecutors, experts, and researchers must assess ecological seriousness, yet the criteria used to structure that assessment often remain implicit, fragmented, or unevenly documented.

The problem is threefold. First, threshold clauses generate legal uncertainty because they distinguish administrative unlawfulness from criminal relevance through evaluative terms such as “substantial damage,” “serious harm,” and “serious risk.” Secondly, the scientific assessment of ecological damage remains empirically demanding, particularly where effects are cumulative, delayed, multi-causal, spatially dispersed, or only probabilistically foreseeable. Thirdly, the concept is structurally fragile because it sits at the intersection of criminal-law guarantees, ecological complexity, and evidential limitations. The issue is therefore not only how environmental criminal law should define “substantial harm,” but also how legal systems can make the assessment of such harm transparent, reproducible, and contestable.

Directive (EU) 2024/1203 improves the European framework by identifying factors relevant to “substantial damage,” likely damage, and non-negligible quantity, but it does not provide a fully operational methodology for translating those factors into everyday prosecutorial, expert, and judicial practice. The argument advanced here is not that quantification eliminates discretion, nor that ecological seriousness can be reduced to a single numerical value. Rather, it is that structured and auditable criteria may improve the quality, transparency, and contestability of threshold reasoning. This is especially important in intermediate cases, where the distinction between administrative unlawfulness and criminal relevance is not self-evident and where legal, ecological, and evidential considerations must be made explicit rather than left implicit in unstructured evaluative reasoning.

### 2.1 Legal uncertainty and threshold clauses

In environmental criminal law, the notion of “substantial harm” fulfils a threshold function: it marks the point at which conduct that is already unlawful in administrative terms acquires the additional gravity required for criminal intervention. That function is neither accidental nor marginal. In systems such as the Spanish one, it is constitutive of the dual sanctioning model itself, because environmental offences often combine a prior breach of environmental regulation with a further clause of criminal significance indicating when the conduct becomes punishable as a crime. As Fuentes Osorio has argued, these clauses are intended to identify the harmful potential that transforms an administrative infringement into a criminal offence, but they do so through open-ended and indeterminate concepts that do not always provide the certainty required by the principle of specificity in criminal law ( Fuentes Osorio, 2019). This has contributed to broader doubts about the practical effectiveness of the dual protection model and, in the Spanish debate, to the claim that criminal environmental protection may in some cases be less effective than administrative protection in remediating ecological damage ( Cuerda Arnau, 2023; Fuentes Osorio, 2023).

The difficulty is not simply lexical. “Substantial harm,” “substantial damage,” “serious harm,” and “serious risk” are threshold expressions that perform a normative sorting function. They are meant to protect criminal law from over-extension, but they also create a zone of interpretative instability in which materially similar cases may be classified differently. For empirical legal studies, this instability is not only a problem of legal doctrine; it is also a problem of observable classification. If the factors that move a case from administrative unlawfulness to criminal significance are not structured transparently, it becomes difficult to compare cases, assess consistency, or evaluate how courts operationalise ecological seriousness in practice.

The doctrinal importance of this problem has increased with the revision of the European framework. Directive 2008/99/EC referred to “substantial damage” without defining the concept, a feature that attracted sustained criticism for its vagueness and for the practical difficulties it created in ecological contexts, especially in aquatic environments ( Rebelo et al., 2024, p. 423). Directive (EU) 2024/1203 improves that position by identifying relevant assessment factors rather than by creating a quantified legal test. Article 3(6) requires Member States to take into account, where relevant, the baseline condition of the affected environment, the temporal profile of the damage, its extent, and its reversibility when assessing whether damage or likely damage is “substantial.” Article 3(7) adds factors relevant to the assessment of likely damage, including the hazardousness of the activity or substance and the extent to which regulatory thresholds have been exceeded; Article 3(8) does the same for negligible and non-negligible quantities. Recital 25 confirms that these are non-exhaustive elements intended to support coherent application, while Recital 46 stresses that Member States should define the scope of administrative and criminal enforcement clearly.

The post-2024 European framework should therefore be understood as an improvement in orientation rather than a full solution to the threshold problem. The Directive narrows the field of discretion by identifying factors that competent authorities must consider, and it strengthens the broader enforcement architecture through obligations relating to resources, training, national strategies, and statistical data. Yet it deliberately leaves room for national translation and case-specific judicial assessment. This is not objectionable in itself. Comparative literature has rightly warned that highly prescriptive models can undermine judicial autonomy and distort penal proportionality ( Poppleton et al., 2021). The difficulty arises when legally decisive concepts remain implicit, fragmented, and methodologically opaque. In such circumstances, discretion is not merely preserved; it becomes difficult to observe, compare, or evaluate.

The Spanish doctrinal debate reinforces this concern. Morelle-Hungría argues that the current structure of environmental offences remains affected by the insufficient integration of scientific knowledge into the offence definition itself, a weakness already identified in earlier work on the difficulty of clarifying the protected legal interest and the level of gravity required by environmental offences ( Morelle-Hungría, 2024; Morelle-Hungría, 2022a). From the ecocentric perspective adopted here, the ecosystem should not be treated merely as a regulatory backdrop but as the substantive core of protection, allowing legal analysis to incorporate system-level ecological values. Eco-criminological arguments reinforce the point: anthropogenic pressures have reached levels that exceed quantifiable planetary boundaries, exposing the limits of legal models that assess ecological wrongdoing solely through traditional categories of individualised injury or abstract risk ( Morelle-Hungría, 2022b). The consequence is not that criminal law must become purely technocratic, but that evaluative legal clauses require better methodological mediation if they are to remain compatible with legality, proportionality, and evidential rationality.

From the standpoint of LEDAT, the implication is straightforward. The model is not presented as a substitute for legal interpretation, nor as a mechanism for converting threshold clauses into fixed numerical rules. It is proposed as a disciplined way of structuring the assessment that threshold clauses already require. Its usefulness depends on whether the criteria identified by Directive (EU) 2024/1203 can be mapped onto transparent legal and ecological variables capable of supporting reasoned, auditable, and contestable decision-making. That mapping must remain methodological rather than determinative: it may improve consistency and comparability, but it cannot by itself resolve the normative question of criminal significance. Without such a transparent mapping, however, the empirical study of environmental criminal thresholds remains limited, because the factors that structure judicial classification remain insufficiently observable.

### 2.2 Empirical uncertainty and the scientific assessment of ecological harm

If legal uncertainty explains why the threshold is contestable, empirical uncertainty explains why it is difficult to prove and difficult to code consistently. “Substantial harm” is an indeterminate legal concept, but it is also an evidentially demanding one. Ecological damage caused by anthropogenic pressures is multidimensional, often cumulative, and not reducible to a single scalar variable. It may involve spatial extent, duration, reversibility, hazardousness, biodiversity effects, ecosystem sensitivity, and interactions between these dimensions. It therefore requires structured methodologies capable of distinguishing levels and intensities of harm while remaining sensitive to ecological context ( Morelle-Hungría, 2020).

Scientific research has made significant progress in this direction. Rebelo et al. develop a multi-criteria decision framework for surface waters that integrates chemical, ecological, and functional variables in order to assess “substantial damage” ( Rebelo et al., 2024). Zhang et al. distinguish between quantifiable and non-quantifiable damage in illegal fishing and show how the absence of standardised assessment criteria undermines the fairness and consistency of judicial compensation ( Zhang et al., 2023). Auffhammer, in a different but relevant domain, shows that climate-damage estimation has evolved towards more sophisticated econometric techniques, while also warning that short-term adaptation effects can distort damage estimates if longer-term dynamics are not adequately modelled ( Auffhammer, 2018). Taken together, this literature supports a modest but important conclusion: ecological harm can be assessed in a more structured way than traditional legal practice often assumes, but scientific measurement does not by itself resolve legal attribution, normative grading, or criminal threshold-setting.

The Directive’s revised architecture makes this point more salient. By requiring Member States to consider baseline condition, duration, extent, reversibility, threshold exceedance, hazardousness, conservation status, and, where relevant, restoration cost, Directive (EU) 2024/1203 implicitly acknowledges that environmental criminal liability depends on ecologically granular forms of reasoning. At the same time, the Directive requires Member States to strengthen the professional and institutional capacity needed to manage that complexity: national authorities must be given sufficient qualified staff and resources; specialised regular training must be provided to judges, prosecutors, police, judicial staff, and competent authorities; national coordination mechanisms must promote a common understanding of the relationship between criminal and administrative enforcement; and systems for anonymised statistical data must be established, with a common Union format to follow. These provisions indicate that the European legislator now expects environmental criminal enforcement to be more methodologically explicit and better documented than under the 2008 framework. For empirical legal studies, this is significant because it shifts attention from abstract legal standards to the observable ways in which institutions identify, document, and classify ecological seriousness.

The Spanish experience illustrates the stakes of this evidential problem. The Prestige litigation remains paradigmatic. Caballero and Soto-Oñate explain that the Supreme Court’s 2016 judgment did not alter the international liability regime, but used domestic criminal liability to strengthen the practical reach of compensation and the “polluter pays” principle ( Caballero & Soto-Oñate, 2017). The Supreme Court ultimately quashed the previous acquittal, convicted the captain for a reckless environmental offence aggravated by catastrophic damage, and deferred the quantification of compensation to the enforcement phase of the judgment ( Supreme Court, 2016, ECLI:ES:TS:2016:11). The later transnational litigation culminating in the judgment of the Court of Justice of the European Union in Case C-700/20 showed, in turn, that the legal determination of ecological damage can shape not only liability but also the enforceability of redress across jurisdictions ( Court of Justice of the European Union, 2022, Case C-700/20). These proceedings do not show that quantification solves causation, attribution, or jurisdictional fragmentation. They show why courts need better-structured ways of reasoning about ecological seriousness when the underlying scientific and procedural record is complex.

For LEDAT, the methodological consequence is that the assessment of ecological harm must be separated from, but connected to, legal and procedural reasoning. The ecological variables should be identifiable as indicators of material ecological gravity; the legal variables should operate as a distinct normative filter within IDPAm; and evidential or procedural constraints should remain within the separate domain of IDPAe. This separation reduces the risk of treating IDPAm as a pure ecological metric when it is, in fact, an integrated legal-ecological indicator. It also prevents the opposite error: treating evidential failure or procedural weakness as if it necessarily implied the absence of materially serious ecological harm.

The model’s credibility therefore depends on a coding structure capable of making uncertainty visible rather than concealing it behind the numerical output. This requires a coding manual with closed anchors, defined evidential sources, rules for incomplete or contested information, inter-coder reliability procedures, sensitivity analysis for default weights, and a strict anti-duplication protocol for triggers. In empirical applications, these safeguards are not secondary technical details. They are necessary conditions for treating LEDAT as a transparent and auditable methodology rather than as an impressionistic scoring exercise.

### 2.3 A necessary but structurally fragile concept

The reference to “substantial harm” is normatively necessary because environmental criminal law requires a threshold capable of preventing the over-criminalisation of trivial or merely technical infringements within a dual sanctioning system. The concept therefore performs an important guarantee function. At the same time, its current formulation is structurally fragile for four reasons.

First, clauses of criminal significance remain vulnerable from the perspective of legality because they do not always provide sufficiently determinate criteria for distinguishing criminal offences from administrative infringements ( Fuentes Osorio, 2019). Secondly, the insufficient integration of scientific evidence into the formulation and application of environmental offences weakens practical enforceability and may contribute to the limited effectiveness of criminal protection, particularly where ecological complexity is not translated into operationally clear criteria ( Morelle-Hungría, 2024). Thirdly, the absence of uniform and replicable methodologies for assessing ecological seriousness continues to generate uncertainty in determining the extent of ecological harm, despite advances in environmental science and damage modelling ( Rebelo et al., 2024; Zhang et al., 2023). Fourthly, judicial interpretation remains decisive not only for criminal liability, but also for the practical scope of remediation and compensation, as comparative and transnational litigation shows ( Caballero & Soto-Oñate, 2017).

What follows is not that threshold clauses should be abandoned, nor that criminal law should surrender its evaluative structure to a formula. The more careful conclusion is that the difficulty lies less in the existence of open language as such than in the absence of a shared bridging methodology capable of operationalising that language in a coherent, transparent, and replicable way. For empirical legal studies, this is the central point: threshold concepts are not only doctrinal categories, but also classification devices whose operation can be studied only if the factors behind their application are made observable.

Directive (EU) 2024/1203 strengthens the case for such a methodology by requiring clearer national delineation between administrative and criminal enforcement, by identifying factors relevant to threshold assessment, and by obliging Member States to develop better statistical and institutional infrastructures. In that sense, the European relevance of LEDAT is immediate, especially in light of the transposition deadline of 21 May 2026 and the subsequent move towards common statistical reporting formats at Union level. However, the significance of the model is not limited to European legal harmonisation. Its broader empirical value lies in providing a structured way to code and compare how legal-ecological seriousness is assessed across cases.

A restrained formulation is therefore preferable. LEDAT should be understood as a structured interpretative aid that may improve the quality of threshold reasoning, particularly in the “grey area” between administrative illegality and criminal significance. In the empirical pilot, this grey area is treated as an indicative analytical band rather than as a legally binding category, and its usefulness depends on whether the model makes the underlying legal and ecological assumptions more transparent. Its authority does not derive from mathematical determinacy, but from methodological discipline: transparent coding rules, explicit evidential anchors, reliability-sensitive coding, sensitivity analysis for the default weights, a strict anti-duplication rule for triggers, and a clear distinction between the ecological component, the legal component, IDPAm, and IDPAe.

Framed in those terms, LEDAT preserves the essential insight of the present article: environmental criminal law needs a bridge between legal indeterminacy and ecological complexity, but that bridge must remain transparent, contestable, non-binding, and compatible with the constitutional safeguards that govern criminal adjudication. The following section therefore turns from the threshold problem to the inadequacy of existing legal, administrative, scientific, and structural approaches when they operate in isolation. This sets the stage for the formal methodological proposal developed later in the article.

## 3. Inadequacy of existing models: normative, epistemological and structural limitations

The inadequacy of existing approaches to ecological damage in criminal law does not stem from a complete absence of analytical tools, but from the fragmentation of the tools that already exist. Administrative thresholds provide useful indicators of regulatory non-compliance, yet they do not by themselves measure criminal seriousness. Case-law assessment remains indispensable, particularly in systems that treat environmental offences as offences of danger, but it is constrained by recurring problems of causation, evidential heterogeneity, and judicial dependence on expert reports. Scientific models demonstrate that ecological damage can be described and quantified with increasing sophistication, although they typically do so in ways that are not directly translatable into criminal-law categories such as threshold seriousness, imputable risk, culpability, or proportionality of punishment. Finally, structural approaches drawn from green and Southern criminology show that ecological harm is distributed through unequal political and economic arrangements, a dimension that purely technical models may overlook.

The problem, therefore, is not that law, science, or criminology lack relevant concepts. It is that their insights are rarely integrated into a common empirical structure capable of supporting transparent comparison across cases. Administrative law identifies breaches; judicial reasoning classifies legal significance; ecological science evaluates environmental impact; and critical criminology situates harm within broader structures of power and inequality. Each perspective captures something important, but none by itself provides a reproducible framework for coding legal-ecological seriousness in environmental criminal adjudication. This fragmentation limits not only legal consistency, but also empirical analysis, because researchers cannot easily observe which dimensions of harm, risk, culpability, evidence, or ecological vulnerability drive threshold assessment.

Directive (EU) 2024/1203 strengthens the European framework by identifying relevant threshold criteria and by requiring better resources, training, coordination, national strategies, and statistical infrastructures. However, it does not itself resolve the methodological gap between ecological measurement and criminal adjudication. Against that background, LEDAT should be framed as a bridging methodology: its purpose is not to replace administrative thresholds, judicial interpretation, scientific modelling, or structural critique, but to organise their legally relevant insights within a transparent, auditable, and contestable framework. Its usefulness therefore depends on explicit coding rules, reliability-sensitive implementation, sensitivity analysis, and a clear distinction between the Material Environmental Criminal Damage Index (IDPAm) and the Enforceability module (IDPAe).

For that reason, the empirical use of LEDAT must be presented as a pilot exercise in structured case ordering, not as full statistical validation of a model. The database used in this article allows the calculation of IDPAm across 36 first-instance judgments, but it does not yet support strong inferential claims about judicial outcomes, sector-level patterns, or predictive performance. The purpose of the empirical application is therefore narrower: to examine whether the model can be applied coherently to real judgments, whether its scores produce a plausible descriptive distribution, and whether its assumptions remain traceable when confronted with heterogeneous environmental cases.

### 3.1 Administrative thresholds and the limits of regulatory objectivity

Administrative environmental law has long relied on quantitative limits—maximum emission values, discharge thresholds, catch quotas, concentration limits, or protected-species restrictions—to discipline economic activity and preserve ecosystem functions. Those thresholds are indispensable within environmental regulation, and they may also serve as evidential anchors in criminal proceedings. Yet they were not designed primarily to grade criminal wrongdoing. Rather, they belong to a broader regulatory logic in which administrative, preventive, civil, and criminal instruments coexist and interact. As Wade et al. observe, environmental sanctions in such systems are often structured around deterrence, compliance, economic benefit, prior history, and restoration rather than around a dogmatically elaborated criminal metric of ecological gravity ( Wade et al., 2002). For that reason, an administrative exceedance may be highly relevant without being conclusive, just as serious ecological damage may occur in situations not perfectly captured by pre-existing thresholds.

For empirical legal analysis, this distinction matters because regulatory thresholds are attractive but potentially misleading measurement devices. They are attractive because they are apparently objective, often quantitative, and relatively easy to code. They are potentially misleading because the fact of exceedance does not necessarily capture duration, reversibility, ecological sensitivity, biodiversity effects, hazardousness, culpability, or the systemic significance of the harm. Treating regulatory breach as equivalent to criminal seriousness would therefore collapse a multidimensional threshold assessment into a single administrative indicator. It would also risk reproducing the limits of the regulatory scheme itself, especially where regulatory parameters fail to capture cumulative or ecosystemic harm.

Directive (EU) 2024/1203 confirms this point at the level of Union law. The recast Directive strengthens the threshold structure of environmental criminal offences and clarifies that, when assessing “substantial damage,” likely damage, or non-negligible quantities, Member States must take into account factors such as the baseline condition of the affected environment, the duration, extent, and reversibility of the damage, the extent to which a regulatory threshold or mandatory parameter has been exceeded, the hazardousness of the activity or substance, the conservation status of the species concerned, and, where feasible, the cost of restoration. In other words, threshold exceedance is treated as an important factor, but not as a self-sufficient criminal metric. The Directive therefore narrows the interpretative field without equating criminal seriousness with regulatory breach alone.

Illegal fishing illustrates this limitation clearly. Morelle-Hungría notes that some forms of IUU fishing may account for a very substantial proportion of catches of certain species, thereby affecting not only the target species itself, but also the broader food webs and ecological balances in which it is embedded ( Morelle-Hungría, 2017, pp. 1, 9). Yet quota breaches or the taking of protected specimens do not necessarily capture the full ecosystemic significance of the harm. Zhang et al. likewise show that the lack of standardised rules for applying guidance documents creates uncertainty in the judicial assessment of ecological damage in illegal fishing cases ( Zhang et al., 2023), while Valeije Álvarez observes that criminal law has been applied only marginally in this domain ( Valeije Álvarez, 2023). The broader lesson is that administrative metrics may identify unlawful conduct, but they do not, without further analysis, provide a reliable measure of criminal relevance.

For LEDAT, the implication is methodological rather than rhetorical. Administrative thresholds should be treated as structured evidential inputs—particularly within ecological variables concerning concentration or volume, hazardousness, and protected status—but not as proxies for criminal seriousness in themselves. The value of a legal-ecological assessment model lies precisely in making explicit how regulatory exceedance interacts with duration, reversibility, ecosystem sensitivity, biodiversity impact, hazardousness, and normative legal factors. In the empirical application, this is why variables such as CONC, SUS, ECO, BIO, and REV are coded separately rather than collapsed into a single indicator of regulatory breach. In that sense, administrative objectivity can support criminal assessment, but it cannot replace it.

### 3.2 Limits of case-law assessment: causation, expert evidence, and the tension between danger and injury

Unlike the forms of loss more commonly addressed by private law, ecological damage in criminal law does not ordinarily converge on a clearly identifiable victim or an easily individualisable proprietary interest. It often affects collective and diffuse goods—ecosystems, biodiversity, the quality of air, soil, and water—whose degradation may develop cumulatively, interact with natural background conditions, and become visible only after a temporal delay. As Silva Sánchez and Montaner Fernández point out, polluting activities frequently generate cumulative or synergistic effects that cannot be reduced to a linear chain of causation ( Silva Sánchez & Montaner Fernández, 2012). Bonorino and Leal emphasise that ecological causation is often proved under conditions of scientific uncertainty, dispersion of sources, and difficulty in attributing both the damage and the responsible conduct with precision ( Bonorino & Leal, 2010). The problem is therefore twofold: proving that ecological harm exists, and proving that a given act is causally relevant within a dynamic environmental system.

These difficulties are intensified by the partial character of the valuation methods that have traditionally informed judicial reasoning. Some approaches privilege the market value of the affected resource; others focus on restoration cost or on associated economic loss. Yet such methods cannot fully capture the ecological, functional, and systemic dimensions of environmental degradation. Abbad and Gutiérrez underline that no comprehensive expert methodology currently translates ecological impacts constituting a criminal offence satisfactorily into economic terms, precisely because ecological damage is not reducible to the loss of an individualisable asset ( Abbad & Gutiérrez, 2015). The same authors also stress that available valuation methods remain partial and incomplete, even where they quantify market value, restoration cost, or derivative losses ( Abbad & Gutiérrez, 2015). Rivera-Olarte reaches a similar conclusion when discussing restoration, noting that the heterogeneity and dynamism of ecological processes resist simplification within traditional frameworks ( Rivera-Olarte, 2017).

In this context, expert evidence becomes decisive. As Górriz Royo argues, expert reports must not only prove pollution and its ecological implications; they must also translate scientific information into legal categories that courts can use ( Górriz Royo, 2015). The result is a hybrid process in which ecological science, technical expertise, and criminal-law reasoning converge, often without a common methodological script. That absence of shared methodological standards can expand interpretative variability and produce materially divergent outcomes in analogous cases.

Spanish case law addresses part of this difficulty by treating environmental offences as offences of danger. Supreme Court Ruling 81/2008 states that the protected legal interest is not merely compliance with administrative regulations, but the environment itself as a collective interest of a supra-individual nature; criminal liability depends on whether the conduct is capable of creating a serious danger to that interest, which in turn requires technical assessment supported by expert evidence ( Supreme Court, 2008, ECLI:ES:TS:2008:1028). Fuentes Osorio correctly notes that the danger-offence structure allows criminal intervention to anticipate irreversible ecological damage, but also that the vagueness of the offence elements makes the criminally relevant danger difficult to define with precision ( Fuentes Osorio, 2012). Górriz Royo adds that the gravity of the danger must ultimately be related to the ecological disturbance caused or threatened, which means that criminal assessment remains deeply dependent on technical judgments about intensity, probability, geographical reach, and duration ( Górriz Royo, 2015).

From an eco-criminological perspective, White is right to insist that ecological harm cannot be addressed solely through a reactive punitive lens. Prevention, contextual analysis, situational controls, and structural understanding remain essential, especially because ecological harm is frequently cumulative and partly irreversible ( White, 2022). That insight is directly relevant to LEDAT. If the tool is to assist legal reasoning rather than obscure it, IDPAm must be understood not as a pure measure of ecological harm, but as an integrated legal-ecological indicator of material seriousness. IDPAe, by contrast, concerns the distinct question of procedural and evidential viability. This separation helps prevent the conflation of substantive gravity with prosecutorial success or judicial outcome. It also allows future applications of the model to distinguish whether a case is grounded in actual injury, technically substantiated danger, or a combination of both, while reserving causal attribution, evidential weaknesses, conformity dynamics, and procedural barriers for the enforceability analysis.

The European relevance of this clarification is considerable. Directive (EU) 2024/1203 does not eliminate judicial discretion, but it does require Member States to ensure sufficient qualified staff and adequate financial, technical, and technological resources for authorities involved in detection, investigation, prosecution, and adjudication; specialised regular training for judges, prosecutors, police, judicial staff, and competent authorities; and strategic and operational coordination between criminal and administrative enforcement bodies. These provisions reinforce the need for explainable and contestable methodologies rather than ad hoc reliance on unstructured expert disagreement. In empirical terms, this also means that any structured assessment model should avoid treating convictions, acquittals, or conformity-based resolutions as simple external validations of ecological seriousness. Judicial outcome is relevant descriptive information, but it remains procedurally mediated and must be analysed separately from the material gravity captured by IDPAm.

### 3.3 Scientific models: analytical progress and legal limits

Scientific and technical research has made substantial progress in the modelling and quantification of ecological harm. Quesada-Ruiz et al. develop spatial models based on Random Forest techniques to identify geographic patterns of environmental crime in the Canary Islands, linking those patterns to land use and socio-economic variables ( Quesada-Ruiz et al., 2023). Cañizares Galarza et al. propose a multi-criteria approach that weights environmental indicators and aggregates them using ordered weighted average operators ( Cañizares Galarza et al., 2021). The Ministry for Ecological Transition has produced a guidance document for determining the significance of environmental damage under Law 26/2007, using variables such as extent, intensity, temporal scale, baseline condition, and risk to human health ( Ministerio para la Transición Ecológica, 2019). These developments show that ecological damage is not analytically intractable: it can be described, modelled, and, to a meaningful extent, quantified.

Yet analytical success in environmental science does not automatically translate into legal adequacy in criminal law. Castañón del Valle notes that the assessment of ecological damage continues to be affected by doctrinal, legal, jurisprudential, and scientific shortcomings, and famously suggests that the traditional civil-liability system is “taking on water” when confronted with environmental damage ( Castañón del Valle, 2006, p. 91). Bonorino and Leal similarly argue that traditional liability regimes are structurally ill-equipped for ecological damage because such damage is diffuse, collective, ongoing, and not easily individualised; even when legal systems alleviate evidential burdens through presumptions or circumstantial reasoning, the underlying complexity of causal attribution does not disappear ( Bonorino & Leal, 2010). The point is not that scientific metrics are useless, but that they generally measure damage in ecological, economic, or managerial terms rather than in categories tailored to criminal-law analysis.

For empirical legal studies, this distinction is crucial. A scientifically sophisticated model may describe ecological degradation accurately while remaining poorly suited to coding how criminal courts assess threshold seriousness. Conversely, a legally useful coding framework need not claim to provide a complete biophysical measure of ecological damage. Its task is different: to translate legally relevant ecological information into variables that can be applied transparently, compared across cases, and challenged by legal actors. The relevant question is therefore not whether scientific models should be replaced by legal coding, but how scientific information can be structured so that its legal significance becomes observable and reproducible.

This is precisely where LEDAT can make a contribution, provided that its methodological architecture remains restrained. The model does not seek to transform ecological criminal adjudication into a purely scientific measurement exercise. Rather, it organises legally relevant ecological information through closed variables, explicit evidential anchors, reliability-sensitive coding, and a transparent integration structure. Its default weighting scheme (Wj = 0.4; We = 0.6) is therefore best understood as a provisional modelling choice rather than as a statistically discovered parameter. In the pilot database, alternative plausible weighting schemes produce highly similar ordinal case rankings, which supports the internal structure at an exploratory level. This finding, however, should be interpreted as a preliminary sensitivity check rather than as statistical calibration or external validation of the model.

Similarly, the trigger system should operate only where the qualitative gravity of the case exceeds what is already captured by the ordinary variables, thereby preventing double counting between biodiversity, ecosystem protection, hazardousness, and similar factors. The empirical application confirms that triggers have a substantive effect on some classifications and are not merely decorative additions to the index. For that reason, their activation must be explicitly justified, capped, and subject to a strict anti-duplication protocol. These safeguards do not make LEDAT deterministic; they make its use more transparent, more contestable, and more coherent with its stated purpose.

Directive (EU) 2024/1203 adds a further reason to formalise those methodological safeguards. The Directive now requires Member States to record, publish, and transmit anonymised statistical data covering reporting, investigation, prosecution, adjudication, dismissals, convictions, legal-person liability, and penalties, with a common Union format to follow. A structured tool that aspires to support environmental criminal analysis should therefore be designed from the outset in a way that is compatible with comparability, traceability, and later empirical review. In practical terms, this requires not only a final IDPAm score, but also a case-level record of the underlying legal variables, ecological variables, trigger activations, evidential sources, coding uncertainty, and procedural outcome.

### 3.4 Structural perspective: ecological justice and global asymmetries

The assessment of ecological damage is not only a technical or doctrinal problem. Green criminology and Southern criminology have shown that ecological harm is also shaped by relations of power, by asymmetries of regulation and enforcement, and by the uneven distribution of ecological burdens across territories and populations. Walters and Fuentes Loureiro argue that ecological damage often follows patterns of structural inequality connected to the global economy; their discussion of hazardous-waste transfers from industrialised countries to regions of the Global South illustrates how ecological costs may be externalised to territories with weaker institutional capacities, while the benefits remain elsewhere ( Walters & Fuentes Loureiro, 2020). In such contexts, legal visibility is not determined by measurable harm alone, but also by geopolitical and institutional power.

For empirical legal analysis, this point matters because case datasets are not neutral mirrors of ecological harm. They are shaped by enforcement priorities, inspection capacity, prosecutorial discretion, access to expert evidence, institutional resources, and the kinds of harm that legal systems are able or willing to recognise. A dataset of judgments therefore captures adjudicated and documented harm, not the full universe of ecological degradation. This does not make judicial datasets unusable, but it requires caution in interpreting what their distributions represent. A low number of cases in a sector, or a low average seriousness score, may reflect lower ecological gravity; it may also reflect under-detection, under-prosecution, evidential barriers, or doctrinal invisibility.

This structural dimension intersects with the conceptual distinction between damage affecting identifiable interests and “pure ecological damage.” Rivera-Olarte stresses that ecological damage may directly affect ecosystems or biodiversity even where no immediate injury to specific persons can be demonstrated ( Rivera-Olarte, 2017). Cafferatta similarly highlights the difficulty of fitting diffuse, collective, and transgenerational ecological harms into traditional legal categories that privilege identifiable victims and patrimonial loss ( Cafferatta, 2004). As a result, some forms of serious ecological degradation remain only partially visible in legal systems, especially where no direct victim can anchor the claim.

Noise pollution is a useful example of that selective visibility. García Ruiz and South show that, although its impacts on well-being, wildlife, and ecological balance are well documented, noise is still predominantly treated as an administrative or regulatory issue rather than as a core instance of ecological criminal harm ( García Ruiz & South, 2019). The pilot database used in this article reinforces the need for caution in interpreting that field: noise-related cases constitute the largest sectoral group in the corpus, but they generally occupy the lower range of IDPAm values. This descriptive result should not be read as a general finding that noise pollution lacks criminal relevance. It reflects the specific evidential and factual configuration of the judgments included in the pilot sample. The significance of the example is therefore not that every under-criminalised environmental harm should automatically be absorbed into criminal law. Rather, it is that the legal salience of ecological harm is culturally, institutionally, and evidentially mediated. Any methodology that claims to structure ecological seriousness must therefore remain alert to the possibility that what appears minor in conventional practice may reflect institutional invisibility, evidential framing, or doctrinal categorisation rather than ecological insignificance.

For LEDAT, the lesson is one of interpretative caution. The index should not be understood as a purely technical score detached from the political economy of ecological harm. Materially similar ecological harms may differ in legal visibility because of enforcement priorities, evidential infrastructures, institutional capacities, procedural routes, conformity-based resolutions, or the diffuse character of the affected good. That recognition does not require converting structural inequality into a scored variable within the model. It does require, however, that LEDAT outputs be interpreted within a broader account of ecological justice and legal recognition, particularly where the protected good is diffuse or where the harm is cumulative and socially normalised.

### 3.5 The need for a bridging methodology

The foregoing discussion suggests that the central problem is not a lack of legal concepts, scientific tools, or critical perspectives in isolation. The problem lies in the weak articulation between them. Administrative law supplies thresholds but not a criminal metric of seriousness. Case law supplies doctrinal categories but often works without a common methodology for translating expert knowledge into consistent threshold reasoning. Scientific models supply increasingly refined forms of ecological measurement but do not, on their own, determine criminal relevance. Structural approaches reveal the unequal production and recognition of ecological harm, yet often remain external to trial-level assessment. Existing models are therefore insufficient not because they are individually useless, but because they remain epistemologically fragmented.

A bridging methodology is needed precisely at that point of fragmentation. For empirical legal studies, such a methodology must do more than restate legal standards or import ecological metrics. It should translate legally relevant ecological information into variables that can be coded, compared, audited, and challenged across cases. It should integrate scientifically measurable dimensions capable of reflecting the complexity of ecological damage; organise those dimensions through legally relevant categories; preserve judicial discretion while making its basis more observable; and distinguish material gravity from procedural and evidential enforceability. This is the explanatory space within which LEDAT is situated.

LEDAT is not presented as a completed solution, nor as a validated decision instrument. It is a structured methodological proposal whose current empirical support remains pilot in nature. Its contribution lies in making explicit the assumptions, variables, and weighting choices that often remain implicit in environmental criminal threshold reasoning. By doing so, the framework seeks to improve transparency and comparability without converting judicial assessment into a mechanical exercise.

In the present article, the pilot application concerns 36 first-instance judgments issued by Criminal Courts and Provincial Courts acting as trial courts according to the applicable procedural rules. The empirical exercise supports only cautious claims: LEDAT can be applied to heterogeneous judgment material, it generates a traceable distribution of IDPAm scores, and it allows descriptive comparison across outcomes and sectors. It does not establish predictive validity, causal effects, or population-level generalisability. Future development should expand the dataset, test inter-coder reliability formally, perform weight and trigger sensitivity analyses, distinguish conformity-based convictions from contested convictions, and incorporate IDPAe systematically in order to explain why some materially serious cases fail evidentially or procedurally. That future inclusion of IDPAe is particularly important if the model is to illuminate the difference between ecological seriousness, procedural viability, and judicial outcome.

## 4. A formal legal-ecological coding framework: The LEDAT model

LEDAT ^®^ (Legal-Ecological Damage Assessment Tool) is best understood as a structured coding framework designed to organise the assessment of criminally relevant ecological harm or risk in environmental cases. Its core contribution lies in combining two analytically distinct dimensions—a Legal Component and an Ecological Component—into a synthetic legal-ecological indicator, the Material Environmental Criminal Damage Index (IDPAm), while preserving a separate Enforceability module (IDPAe) for procedural and evidential viability.

The model is not presented as a fully validated decision instrument, but as a transparent and auditable framework for structuring threshold reasoning in environmental criminal law. The default weightings (Wj = 0.4; We = 0.6) and the capped trigger system (+2 maximum) are treated as provisional modelling choices. They are normatively defensible because they prioritise ecological materiality while preserving a legal filter and limiting disproportionate escalation. In empirical applications, however, their performance must be assessed through sensitivity checks, case-level traceability, and reliability-sensitive implementation rather than assumed as fixed parameters.

The methodological credibility of LEDAT depends on four conditions: explicit coding rules, reliability-sensitive implementation, a strict anti-duplication protocol for triggers, and a clear distinction between ecological/material assessment, normative/legal grading, and procedural enforceability. These requirements are especially pertinent in light of Directive (EU) 2024/1203, which strengthens the enforcement framework across the European Union, requires transposition by 21 May 2026, and obliges Member States to improve resources, training, coordination, national strategies, and statistical comparability, while still leaving national authorities with the practical task of operationalising ecological seriousness.

**Figure 1.**
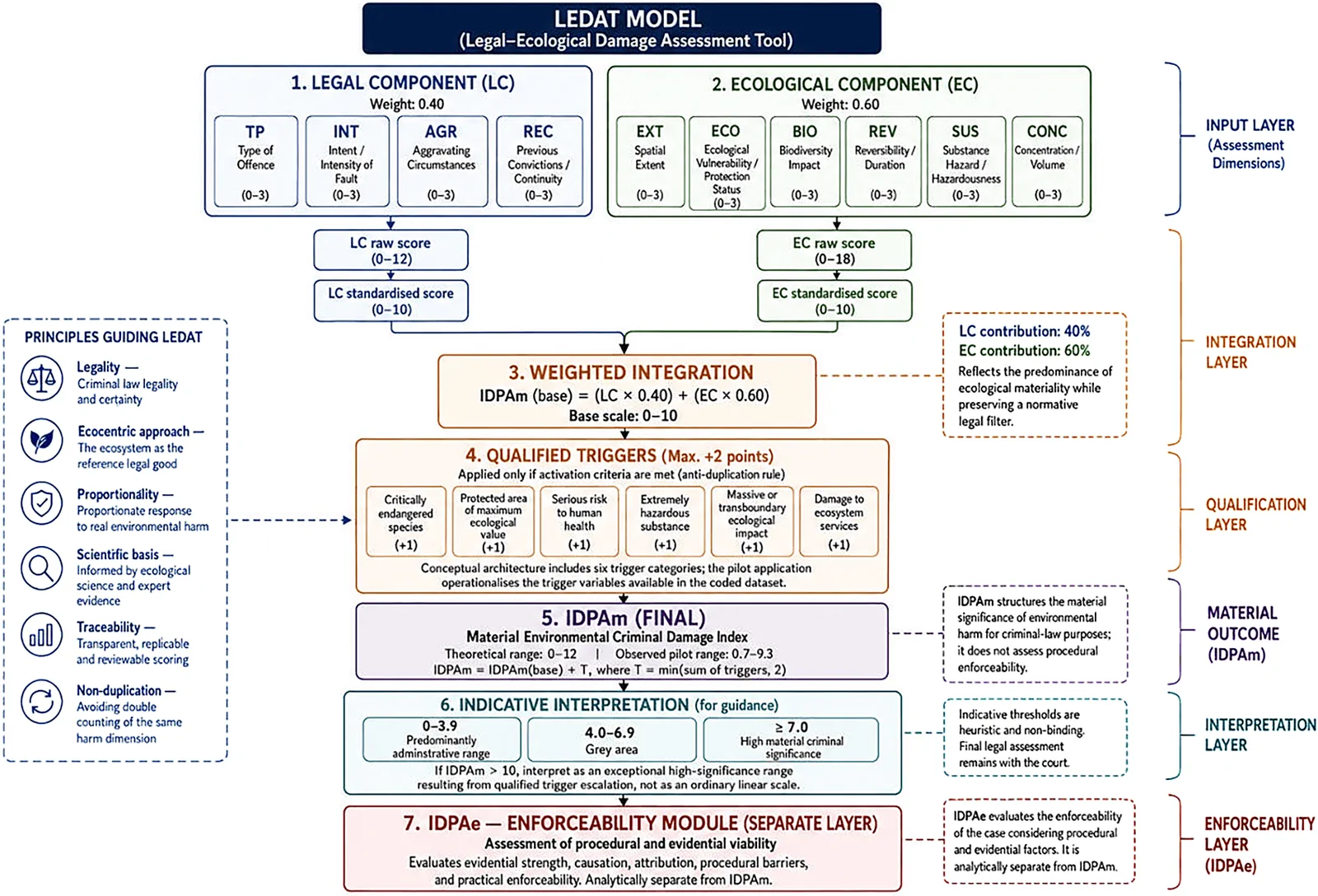
Conceptual architecture of the LEDAT model and integration process for the Material Environmental Criminal Damage Index (IDPAm). The figure distinguishes the Legal Component (LC), the Ecological Component (EC), weighted integration, the capped trigger adjustment, the final IDPAm score, indicative interpretive bands, and the separate Enforceability module (IDPAe). The IDPAm theoretical range is 0–12, while the observed pilot range is 0.7–9.3.

Figure 1 summarises the conceptual architecture of LEDAT and illustrates the interaction between the Legal Component (LC), the Ecological Component (EC), the qualified trigger system, and the distinction between the material and enforceability dimensions of the model.

### 4.1 Purpose, scope, and conceptual structure

To address the enduring indeterminacy of clauses such as “substantial damage,” “serious harm,” and “serious risk” in environmental criminal law, LEDAT ^®^ is proposed as a formalised legal-ecological coding framework. Its purpose is not to replace legal classification, evidential assessment, or judicial discretion, but to structure the analysis of the additional seriousness that distinguishes administrative unlawfulness from criminal relevance. In that sense, LEDAT is designed to make threshold reasoning more transparent, auditable, and comparable across cases.

Methodologically, LEDAT should be understood as a formative composite index rather than as a reflective latent-variable scale. The variables included in the model are not assumed to be interchangeable manifestations of a single hidden construct. Instead, they represent distinct legal and ecological dimensions that jointly contribute to the structured assessment of material environmental criminal seriousness. Consequently, the purpose of the model is not to maximise internal consistency between variables, but to organise heterogeneous yet legally relevant indicators within a transparent and auditable analytical framework. Classical psychometric assumptions associated with reflective scales—such as high inter-item correlation or dimensional unidimensionality—are therefore not treated here as conditions of validity.

Conceptually, the model operates across three levels. The first is the Legal Component (LC), which captures the normative seriousness of the case within the framework of Articles 325–327 of the Spanish Criminal Code and associated criminal-law doctrine. The second is the Ecological Component (EC), which captures the material magnitude of the ecological harm or risk through objectifiable scientific variables. The third level is structured integration, through which the two components are combined into the Material Environmental Criminal Damage Index (IDPAm). In empirical applications, this integration should be interpreted as an ordinal and comparative structuring device rather than as a precise measurement of ecological damage in a strict biophysical sense.

This architecture remains consistent with the revised European framework. Directive (EU) 2024/1203 identifies harm-related factors such as baseline condition, duration, extent, reversibility, hazardousness, conservation status, and threshold exceedance, but leaves Member States and courts with the task of operationalising them in concrete enforcement settings. LEDAT is intended to contribute to that operationalisation in a disciplined, transparent, and contestable way. Its credibility therefore depends less on internal psychometric consistency than on the clarity of its coding rules, the traceability of its evidential anchors, the reliability of its application across coders, and the stability of its outputs under plausible alternative modelling assumptions.

### 4.2 The legal component

The Legal Component (LC) is defined as a structured coding component of normative criminal seriousness, not as a measure of ecological harm. It comprises four closed variables, each scored on a scale from 0 to 3: offence type (TP), intent (INT), aggravating circumstances (AGR), and recidivism or continuity (REC).

The raw legal score ranges from 0 to 12 and is standardised to a 0–10 scale:

LCraw=TP+INT+AGR+REC

LC=(LCraw/12)×10

LC therefore captures the normative gravity of the case as criminal law perceives it, including offence structure, culpability-relevant elements, aggravating circumstances, and continuity or repetition of conduct. It does not measure ecological impact as such. This distinction is essential because IDPAm is not a biophysical damage metric, but an integrated legal-ecological indicator of material criminal seriousness.

For empirical purposes, LC should also be understood as one component of the index rather than as an independent validation criterion. Some of its variables are closely connected to judicial reasoning on liability, especially offence classification, aggravation, and subjective elements. Consequently, when IDPAm is compared descriptively with convictions or acquittals, that comparison cannot be treated as an external test of predictive validity. The Legal Component partly reconstructs the normative structure of the case, and this must be kept separate from claims about judicial outcome. In other words, outcome comparisons are descriptive checks of how IDPAm distributions relate to adjudicated results, not validation tests of the model.

The clarification is also important from the perspective of legality and proportionality, since Directive (EU) 2024/1203 itself distinguishes between offence definitions, aggravating circumstances, sanctions, restoration-related measures, and broader enforcement obligations.

### 4.3 The ecological component

The Ecological Component (EC) is defined as the model’s structured coding component of material ecological gravity or risk, independent of culpability. It consists of six closed scientific variables, each scored from 0 to 3: extent (EXT), ecosystem sensitivity or protection status (ECO), biodiversity impact (BIO), reversibility or duration (REV), substance type or intrinsic hazardousness (SUS), and concentration or volume (CONC).

The raw ecological score ranges from 0 to 18 and is standardised to a 0–10 scale:

ECraw=EXT+ECO+BIO+REV+SUS+CONC

EC=(ECraw/18)×10

This structure aligns with both ecological logic and the revised European framework. Directive (EU) 2024/1203 requires competent authorities, where relevant, to consider the condition of the affected environment, the duration, extent, and reversibility of the damage, the extent to which legal thresholds have been exceeded, the hazardousness of the conduct or substance, and the conservation status of affected species when assessing seriousness. These elements map naturally onto ECO, REV, EXT, CONC, SUS, and BIO.

The value of LEDAT depends on turning this conceptual correspondence into operational coding rules. For that reason, each variable requires closed anchors, admissible evidential sources, rules for handling uncertainty, and criteria for resolving incomplete or contested information. The coding manual should therefore operate as an essential component of the model rather than as an optional supplement. In empirical applications, the EC score should be interpreted as a structured ordinal-comparative coding of ecological seriousness, not as a direct biophysical measurement of damage. This distinction is important because the six variables organise heterogeneous ecological dimensions that are legally relevant but not necessarily commensurable in a strict scientific sense (see Table 1).

**Table 1.** Mapping Directive (EU) 2024/1203 criteria onto LEDAT variables.

Directive (EU) 2024/1203 criterion	LEDAT variable	Operational logic
Baseline condition of the affected environment	ECO/REV	Establishes the ecological context against which degradation or recovery must be assessed
Duration of damage or likely damage	REV	Captures temporal persistence and long-term ecological alteration
Extent of damage or likely damage	EXT	Measures spatial reach or territorial scale of the affected area
Reversibility of damage	REV	Distinguishes short-term, long-term, and irreversible ecological effects
Exceedance of regulatory thresholds or mandatory parameters	CONC	Uses legal or technical limits as evidential inputs without treating them as conclusive of criminal seriousness
Hazardousness of the activity or substance	SUS	Captures toxicological, persistence, bioaccumulative, carcinogenic, or equivalent risk properties
Conservation status of affected species	BIO	Reflects biodiversity vulnerability and protected-species relevance
Protected status or ecological sensitivity of affected area	ECO	Captures Natura 2000 areas, National Parks, SPA/SAC, or equivalent protection status
Restoration cost, where feasible	REV/ECO/IDPAe	May inform reversibility, ecological seriousness, and enforceability, but does not replace ecological assessment
Aggravating circumstances under Directive logic	AGR/triggers	Captures legally relevant aggravation while avoiding double counting with ordinary variables

Table 2 summarises the harmonised scoring structure of the LEDAT model. It integrates the legal and ecological variables into a single operational matrix, while preserving the distinction between normative criminal relevance and material ecological severity. The score anchors are intended as coding rules for structured assessment; they do not create autonomous legal thresholds and should be applied with explicit reference to the evidential material available in each case.

**Table 2.** Harmonised LEDAT variables and scoring matrix.

Component	Variable	Score 0	Score 1	Score 2	Score 3
Legal Component (LC)	TP — Offence classification	Administrative offence or doubtful criminality	Article 325 CC	Article 326 CC	Article 327 CC
Legal Component (LC)	INT — Intent	Minor negligence	Gross negligence	Recklessness/indirect intent	Direct intent
Legal Component (LC)	AGR — Aggravating circumstances	None	One aggravating factor	Two aggravating factors	Three or more/continuing offence
Legal Component (LC)	REC — Recidivism/continuity	No previous conviction or pattern	Isolated previous conviction	Multiple recidivism	Systematic or structural conduct
Ecological Component (EC)	EXT — Spatial extent	<0.5 ha or minimal impact	0.5–1 ha	1–10 ha	>10 ha or widespread impact
Ecological Component (EC)	ECO — Ecosystem sensitivity	Common or urban area	Transformed or agricultural area	Sensitive ecosystem	Protected area: Natura 2000, National Park, SPA/SAC
Ecological Component (EC)	BIO — Biodiversity impact	Common species	Regionally protected species	Vulnerable species	Endangered, CITES-listed or critically endangered species
Ecological Component (EC)	REV — Reversibility/duration	<1 year	1–5 years	>5 years	Irreversible
Ecological Component (EC)	SUS — Substance hazard	Non-toxic	Toxic	Hazardous	Persistent, bioaccumulative or extremely hazardous
Ecological Component (EC)	CONC — Concentration/volume	Within legal limits	Slight excess	High excess	Critical or massive excess

Because the pilot database applies these variables to heterogeneous judicial material, Table 2 should be read as a harmonised coding matrix rather than as a claim that all sectors generate the same evidential profile. For example, CONC may be central in discharge, waste, or emission cases, while BIO or ECO may carry greater interpretive weight in protected-species or protected-area cases. The standardised EC score permits comparison across cases, but the case-level coding annex should preserve the factual and evidential justification for each assigned value.

### 4.4 Structured integration and default weighting of the model

The weighted integration of LC and EC generates the ordinary 0–10 base scale of the model, while the trigger system operates as a capped exceptional increment designed to capture qualitative ecological escalations that may not be adequately reflected through linear aggregation alone.

Once standardised, the two components are combined according to the following structure:

IDPAmbase=(Wj×LC)+(We×EC)

T=min(∑triggers,2)

IDPAm=IDPAmbase+T

where

IDPAmbase∈[0,10]

T∈[0,2]

IDPAm∈[0,12]

In this formulation, LC represents the Legal Component, EC the Ecological Component, WjWj the legal weighting coefficient, We the ecological weighting coefficient, and TT the qualified trigger adjustment. Mathematically, the final IDPAm may therefore range from 0 to 12. Conceptually, however, the model should be understood as a 0–10 ordinary base scale with a capped exceptional increment of up to +2 for qualitatively aggravated ecological scenarios.

Because LEDAT operates as a formative composite methodology, the weighting structure does not seek to estimate latent covariance between variables. The weights instead express a transparent modelling assumption about the relative contribution of legal and ecological dimensions to the structured assessment of material criminal seriousness. Accordingly, the proposed weighting scheme should be interpreted as a theory-driven and contestable modelling choice open to future empirical refinement rather than as a statistically discovered parameter.

The default values—Wj = 0.4 and We = 0.6—are proposed as provisional defaults. Because the index is intended to structure the assessment of material ecological seriousness, the ecological dimension carries greater weight than the legal one. At the same time, criminal significance cannot be reduced to ecological measurement alone, since offence structure, culpability, aggravation, and continuity remain integral to criminal-law reasoning. The default weighting therefore reflects the model’s dual purpose: prioritising ecological materiality while preserving a normative legal filter.

The empirical rationale is necessarily provisional. In the pilot application reported later in the article, the proposed weighting appears descriptively consistent with the aim of distinguishing predominantly administrative cases, an intermediate grey area of debatable criminal significance, and a smaller set of materially more serious cases. In the database analysed here, IDPAm values range from 0.7 to 9.3, and most cases fall below the high-significance band. This supports the internal plausibility of the scale as a descriptive ordering device, but it does not validate the weighting structure in a definitive statistical sense.

Future iterations of the model should therefore examine whether case ranking and interpretive classification remain stable under plausible alternative weighting structures, including 0.5/0.5 and 0.3/0.7, and whether the relative contribution of LC and EC varies across different sectors of environmental offending. In the present pilot, those alternatives are used only as sensitivity checks. The results suggest substantial ordinal stability under alternative legal/ecological weights, whereas the removal of triggers has a more visible effect on band classification. This confirms that the weighting scheme and the trigger system should be analysed separately: the former structures the ordinary integration of legal and ecological seriousness, while the latter captures capped qualitative escalation.

The numerical structure of LEDAT should not be interpreted as expressing mathematical precision in the strict scientific sense. The purpose of quantification within the model is organisational and comparative rather than deterministic. Numerical integration is used here as a transparent mechanism for structuring heterogeneous legal and ecological considerations that would otherwise remain implicit, fragmented, or inconsistently articulated in environmental criminal assessment. Because the underlying variables are ordinal and partly theory-driven, the resulting numerical outputs should be interpreted as structured ordinal-comparative approximations rather than metrically exact measurements of ecological criminal seriousness.

### 4.5 Trigger activation, anti-duplication rules, and interpretive bands

The trigger system operates as a capped qualitative adjustment designed to capture exceptional ecological features that may not be adequately reflected through ordinary linear aggregation. Its function is not to multiply severity mechanically, but to make visible qualified forms of ecological escalation that exceed the ordinary coding of the Legal Component and the Ecological Component.

Triggers may be activated only where there is explicit evidential support and where the relevant qualitative factor is not already fully captured by the ordinary variable scores. The cumulative trigger adjustment is capped according to the rule:

T=min(∑triggers,2)

This means that, regardless of the number of activated triggers, the final adjustment cannot exceed +2. The cap is intended to preserve proportionality and prevent exceptional qualitative factors from overwhelming the ordinary weighted structure of the model.

In Table 3 the cumulative trigger adjustment is limited according to the rule T = min(∑triggers, 2). Triggers operate as qualitative corrective mechanisms in scenarios where linear integration may underestimate material ecological gravity. Their application requires explicit justification and cannot duplicate dimensions already fully incorporated into the Legal Component (LC) or Ecological Component (EC). The conceptual protocol distinguishes the six trigger categories listed above. In the pilot dataset, these categories were operationalised through the available trigger variables T1–T4, with the capped value T calculated from their cumulative activation. Future applications should disaggregate trigger categories more explicitly at the case level.

**Table 3.** Qualified trigger activation protocol and anti-duplication safeguards.

Trigger	Activation condition	Additional score	Anti-duplication safeguard
Critically endangered species	Impact affecting species classified as critically endangered, CITES Appendix I, or equivalent	1	Cannot duplicate the BIO variable where biodiversity severity is already fully captured
Protected area of maximum ecological value	Impact occurring within National Parks, Natura 2000 core areas, SPA/SAC, or equivalent maximum-protection areas	1	Cannot duplicate the ECO variable where ecosystem protection is already fully reflected
Serious risk to human health	Scientifically supported evidence of severe or widespread human health exposure	1	Requires independent evidential justification beyond pollutant concentration
Extremely hazardous substance	Presence of persistent, bioaccumulative, carcinogenic, mutagenic, or highly toxic substances generating exceptional ecological risk	1	Cannot duplicate the SUS variable where intrinsic hazard has already been fully weighted
Massive or transboundary ecological impact	Ecological effects extending beyond the immediate affected area or producing large-scale ecosystemic disruption	1	Requires demonstrable ecosystemic amplification beyond the EXT variable
Damage to ecosystem services	Significant disruption of essential ecosystem functions, including water purification, habitat integrity, trophic balance, or equivalent functions	1	Must reflect qualitative ecosystemic effects not already captured quantitatively

The anti-duplication rule is central to the model. A trigger may operate only when the relevant qualitative factor is not already fully exhausted by the ordinary score assigned to the corresponding variable, or where its legal or ecological significance goes beyond what the ordinary variable can capture. Otherwise, the model would risk double counting the same feature through, for example, BIO and a species-based trigger, ECO and a protected-area trigger, or SUS and a hazardous-substance trigger.

This logic mirrors the structure of Directive (EU) 2024/1203, which distinguishes between offence elements and aggravating circumstances and requires that aggravating circumstances apply only insofar as they do not already form part of the constituent elements of the offence. LEDAT follows the same principle: triggers are not cumulative severity multipliers, but capped qualitative corrective mechanisms. Their role is therefore not to inflate the score mechanically, but to ensure that exceptional features that are not sufficiently captured by the ordinary variables remain visible, auditable, and contestable.

The proposed interpretive bands—0–3.9 as predominantly administrative, 4–6.9 as a grey area, and ≥7 as high material criminal significance—are heuristic, indicative, and non-binding. Their function is not to create new legal thresholds, but to order the gradation of seriousness already implicit in criminal-law doctrine and in the Directive’s reliance on qualitative threshold concepts. In empirical applications, the bands are applied to the final IDPAm score, including any capped trigger adjustment.

Where qualified triggers increase the score above 10, the case should be understood as falling within an exceptional high-significance range reflecting qualitative ecological escalation rather than ordinary linear accumulation. In the present pilot, however, the observed IDPAm range remains within 0.7–9.3, so no case exceeds the ordinary 0–10 range after trigger adjustment.

For empirical legal analysis, the trigger system has two implications. First, trigger activation must be documented at case level, including the evidential basis for each activation and the reason why the anti-duplication rule is satisfied. Secondly, sensitivity checks should examine how classifications change when triggers are removed or modified. In the pilot application, this issue is especially important because trigger removal has a more visible effect on band classification than alternative legal/ecological weighting schemes.

### 4.6 IDPAm and IDPAe: substance and enforceability

One of the strongest features of LEDAT is its distinction between the Material Environmental Criminal Damage Index (IDPAm) and the Enforceability module (IDPAe). IDPAm concerns the substantive or material gravity of ecological harm or risk as structured through the combined legal and ecological dimensions. IDPAe, by contrast, concerns the practical viability of sustaining a criminal charge in light of procedural and evidential constraints, including limitation periods, nullity risks, weakness of expert evidence, causal uncertainty, attribution problems, conformity dynamics, or defects in the chain of custody.

This separation prevents a common analytical error: equating acquittal or procedural failure with the absence of material criminal seriousness. A case may be ecologically grave yet evidentially weak; conversely, a procedurally robust case may concern only moderate material gravity. For that reason, IDPAm and IDPAe should not be collapsed into a single indicator. Nor should judicial outcome be treated as a direct validation of IDPAm, since convictions, acquittals, and conformity-based resolutions are shaped by procedural, evidential, strategic, and institutional factors that extend beyond material ecological seriousness.

For empirical legal studies, this distinction is especially important. Judicial outcomes are observable and therefore tempting as validation criteria, but they are not clean measures of ecological seriousness. A conviction may reflect strong evidence, negotiated conformity, prosecutorial strategy, or moderate but easily provable harm. An acquittal may reflect weak causation, evidential insufficiency, procedural defects, or attribution problems, rather than low material seriousness. Outcome comparisons can therefore be descriptively informative, but they cannot establish predictive validity or external validation of IDPAm without a separate account of enforceability.

The minimum operationalisation of IDPAe should include structural obstacles, procedural validity of key evidence, expert robustness, causal link and objective attribution, perpetration or organisational attribution, chain-of-custody reliability, and the procedural form of case resolution. However, the present empirical application is limited to IDPAm. IDPAe is not systematically applied in the pilot because publicly available judgments do not provide sufficiently uniform access to the full evidential and procedural record. This limitation is particularly relevant where cases end by conformity or where acquittals may reflect evidential insufficiency rather than low material seriousness. Future validation should incorporate IDPAe systematically, precisely because it is the module best suited to explaining divergence between material seriousness, procedural viability, and judicial outcome.

### 4.7 Coding protocol, implementation formats, and methodological transparency

LEDAT can be implemented in several formats: a structured expert matrix, a spreadsheet-based tool, or an open computational module. The choice of format is secondary to the conditions that make the framework scientifically useful and legally accountable. Whatever the format, the model must be explainable, traceable, and contestable. It cannot operate as a black box.

Those requirements are not merely good practice. They are consistent with Directive (EU) 2024/1203’s broader insistence on resources, specialised training, coordination, risk-based national strategy, and statistical comparability. A structured methodology that may influence criminal adjudication must remain open to contradiction by the parties and to judicial scrutiny. In empirical legal research, the same principle translates into reproducibility: other researchers must be able to understand how variables were assigned, how scores were calculated, and how final classifications were produced.

For that reason, implementation should include an auditable record of who entered or modified each variable, preservation of version history, identification of the evidential basis for every assigned value, a short justification for each score, and a clear statement of any assumptions made where the underlying record is incomplete. The coding manual should contain not only scoring anchors, but also decision rules for missing data, rules for conflict between documentary and expert evidence, and the anti-duplication protocol for triggers.

In empirical applications, the dataset should also preserve the variables needed to reproduce the final score: the individual LC variables, the individual EC variables, each trigger activation, the capped trigger value, the calculated LC, EC, IDPAm base score, final IDPAm, interpretive band, court, year, sector, outcome, and procedural disposition where relevant. This is particularly important when the model is applied to heterogeneous judicial material, because the final numerical value is meaningful only if the underlying coding trail remains available for audit, replication, and challenge.

For the pilot application reported in this article, the derived case-level dataset, methodological appendix, and replication scripts are treated as part of the empirical contribution rather than as ancillary materials. The purpose of these materials is not to create a black-box implementation of LEDAT, but to allow readers to verify the descriptive statistics, reproduce the reported figures and sensitivity checks, and evaluate the coding assumptions underlying the pilot.

### 4.8 Inter-coder reliability and pilot application

Because LEDAT is presented as a replicable coding methodology, the coding process must be treated as reliability-sensitive. In the present exploratory application, cases were coded independently by two trained junior researchers using a shared coding manual and an independent parallel coding protocol. The coders worked separately and without access to each other’s classifications before reconciliation. The coded cases were subsequently reviewed and reconciled through consensus. This procedure was designed to reduce individual interpretative bias and to test whether the coding framework could be applied coherently to heterogeneous judgment material.

The pilot does not yet report a formal inter-coder reliability coefficient. Accordingly, the reported coding convergence should be understood as descriptive and provisional. This is an important limitation. Because LEDAT seeks to reduce interpretative arbitrariness through structured coding, future applications should report formal agreement measures before reconciliation. Cohen’s kappa may be appropriate for paired coders and categorical variables, while Krippendorff’s alpha may be appropriate where variable structure, missingness, or coding design make it preferable. For continuous or quasi-continuous outputs such as LC, EC, and IDPAm, future work should also consider reporting intraclass correlation coefficients or equivalent agreement measures.

The absence of a formally reported inter-coder reliability coefficient affects the status of the empirical claims advanced here. The present application should therefore be understood as a feasibility-oriented coding exercise rather than as a full reliability validation of the model. It tests whether the framework can be applied to real judicial material, whether the resulting scores generate a traceable descriptive distribution, and whether the assumptions underlying the model remain visible when applied across heterogeneous cases. It does not establish that different coders would reproduce the same results across larger samples without further training, calibration, and formal reliability testing.

The same caution applies to claims of validation more generally. The present empirical application remains a pilot. It is based on 36 first-instance judgments issued by Criminal Courts and Provincial Courts acting as trial courts according to the applicable procedural rules. It offers an initial test of usability, coherence, and comparative ordering, but not full external validation. The pilot also does not yet incorporate IDPAe systematically and therefore cannot fully explain the relationship between material seriousness, evidential viability, procedural disposition, and judicial outcome. This limitation is especially important for JELS readers because the empirical contribution lies in structured case ordering and methodological transparency, not in prediction, causal inference, or population-level generalisation.

### 4.9 Structural safeguards and legal nature of the model

The use of structured matrices and integration formulas in criminal-law analysis requires a clear account of legal nature and constitutional limits. LEDAT should therefore be characterised as a technical-expert support tool, an argument-structuring framework, and a replicable research instrument. It is neither a sentencing grid nor an automated decision-making system. It does not create new offences, redefine the content of Articles 325–327 of the Spanish Criminal Code, or displace the court’s freedom to assess the evidence. Rather, it organises factors that are already relevant in environmental criminal law: the seriousness of the harm or danger, the structure of the offence, the subjective form of the conduct, aggravating factors, the protected status of ecosystems or species, and the objective features of the ecological impact.

For empirical legal studies, this legal characterisation is also methodologically important. If LEDAT were treated as a decision rule, its scores would appear to dictate legal outcomes. If it were treated as a purely ecological metric, its legal component and normative assumptions would become obscured. If it were treated as a predictive model, the pilot would be judged against standards it does not claim to satisfy. The model is instead best understood as a structured framework for coding and comparing the legal-ecological seriousness of cases. Its outputs are therefore interpretive and comparative, not dispositive.

On that basis, LEDAT is compatible with legality because it does not add new offence elements; with minimal intervention because it is designed precisely to structure the distinction between administrative illegality and criminal significance; with culpability because it does not quantify moral blame in the abstract but organises already relevant elements of intent, continuity, and aggravation; and with proportionality because it seeks comparative consistency rather than illusory mathematical precision. These claims remain persuasive only if the model remains non-binding, transparent, and open to challenge ( Fuentes Osorio, 2019; Wade et al., 2002; White, 2022; Zhang et al., 2023; Rebelo et al., 2024; Auffhammer, 2018).

LEDAT is therefore most convincing when described as a structured methodology for improving the traceability and quality of reasoning in environmental criminal cases, especially in the grey area between administrative and criminal unlawfulness. It becomes less convincing if presented as though the present pilot had already established full validation. Its next stage should consist not in stronger claims, but in stricter testing: formal reliability reporting, sensitivity analysis, fuller documentation of coding decisions, clearer variable mapping to the Directive and to administrative thresholds, separation between conformity-based and contested outcomes, and systematic inclusion of IDPAe in future empirical validation.

LEDAT does not assume that ecological criminal seriousness can be reduced exhaustively to numerical representation. Rather, the model proceeds from the more limited premise that structured and transparent approximation may improve the consistency and traceability of legal reasoning under conditions of ecological and evidential complexity. The empirical pilot should therefore be read as an initial feasibility and coherence exercise: it shows that the model can be applied to a heterogeneous set of 36 first-instance judgments and can generate a reproducible descriptive ordering, but it does not establish predictive validity, causal explanation, or definitive empirical validation.

## 5. Pilot empirical application to Spanish environmental crime judgments

This section reports a pilot empirical application of LEDAT to Spanish first-instance environmental crime judgments. Its purpose is limited and methodological: to assess whether the framework can be operationalised in a transparent and replicable way, and whether its outputs provide an internally plausible descriptive ordering of cases according to the Material Environmental Criminal Damage Index (IDPAm). The empirical exercise is therefore exploratory rather than definitive. It is not designed to estimate the prevalence of environmental crime, predict judicial outcomes, or validate LEDAT as a decision instrument.

The search strategy covers the period from 1 January 2020 to 31 December 2025 and yields a final valid sample of 36 judgments issued by Criminal Courts and Provincial Courts acting as trial courts according to the applicable procedural rules. Cases were coded independently by two junior researchers using a shared coding manual and an independent parallel coding protocol in which coders worked separately and without access to each other’s classifications before reconciliation. Because a formal inter-coder reliability coefficient has not yet been calculated, the present results should be read as provisional pending future reporting of Cohen’s kappa or Krippendorff’s alpha.

The pilot applies the Legal Component (LC), the Ecological Component (EC), and the capped trigger system in order to calculate IDPAm. The Enforceability module (IDPAe) is not applied in this phase because publicly available judgments do not provide sufficiently uniform access to the full evidential and procedural record. The descriptive findings show that most cases fall within the predominantly administrative or intermediate grey-area bands of the index, while four cases reach the high material criminal significance band. Higher IDPAm values are descriptively associated with convictions within the pilot sample, but the overlap between convictions and acquittals is substantial and the association is attenuated when conformity-based convictions are distinguished from contested convictions. No predictive, causal, or inferential claim is advanced.

### 5.1 Sample selection and search strategy

The empirical basis for the pilot study was drawn from the Tirant Prime jurisprudential database, accessed through the institutional portal of the Universitat Jaume I. The temporal scope extends from 1 January 2020 to 31 December 2025. The search strategy was designed to identify criminal proceedings potentially related to environmental offences and used the Boolean string: “artículo 325” OR “art. 325” OR “delito contra el medio ambiente”. Jurisdictional filters were restricted to criminal courts, including High Courts of Justice, Provincial Courts, and criminal courts competent to adjudicate first-instance environmental criminal proceedings.

The initial search yielded 1,145 documents. An internal filter for first-instance decisions reduced the working set to 157 judgments. A subsequent qualitative screening excluded cases in which the reference to Article 325 of the Criminal Code was only tangential, formally pleaded but legally irrelevant to the final judgment, or subsumed into unrelated offences. On that basis, 121 judgments were discarded, producing a final valid sample of n = 36n = 36 decisions suitable for systematic coding.

The final corpus consists of first-instance judgments in the procedural sense: that is, judgments issued by the court competent to adjudicate the case at trial level, regardless of whether that body was a Criminal Court or a Provincial Court. In the final coded sample, 26 judgments were issued by Provincial Courts and 10 by Criminal Courts. No High Court of Justice decision was retained in the final valid corpus. The annual distribution of the sample was uneven: 12 judgments correspond to 2020, 10 to 2021, 4 to 2022, 4 to 2023, 4 to 2024, and 2 to 2025.

The final sample should therefore be understood as a filtered jurisprudential corpus rather than as a complete census of Spanish environmental criminal litigation between 2020 and 2025. The search protocol was designed to maximise recall, but it remains dependent on database indexing practices, search terms, procedural classification, and the availability of written judgments. It may over-include documents in which Article 325 appears only incidentally and under-include relevant cases not indexed under the chosen terms. This qualification does not diminish the usefulness of the pilot; it clarifies its scope. The dataset is appropriate for testing whether LEDAT can be applied coherently to real judicial material, but not for estimating the population-level frequency or distribution of Spanish environmental criminal cases.

### 5.2 Coding protocol and calculation of the index

Each of the 36 judgments was coded using the LEDAT framework. The coding process was conducted independently by two trained junior researchers on the basis of a shared coding manual defining the variables, evidential anchors, scoring rules, and trigger activation criteria. The principal researcher supervised the design of the protocol, reviewed discrepancies after independent coding, and conducted final harmonisation of the dataset.

To reduce individual interpretative bias, all cases were coded independently before the reconciliation phase. The coding protocol should therefore be understood as an independent parallel coding procedure rather than as a fully blind design in the strict experimental sense, since judgments normally disclose procedural outcome, factual reasoning, legal classification, and evidential assessment. Discrepancies in variable assignment were subsequently reviewed jointly and resolved through consensus-based discussion grounded in the predefined coding criteria and in the documentary evidence contained in the judgments (see Table 4).

**Table 4.** Coding protocol and inter-rater reliability structure used in the pilot application of LEDAT.

Phase	Researcher(s) involved	Function
Protocol design and calibration	Principal researcher	Preparation of coding manual, variable definitions, and scoring criteria
Independent parallel coding	Researcher A	Independent application of the LEDAT matrix
Independent parallel coding	Researcher B	Independent application of the LEDAT matrix
Discrepancy review	Principal researcher and coders	Comparative analysis of divergent scores
Reconciliation phase	Joint review	Consensus-based final assignment of variables
Final harmonisation	Principal researcher	Consistency verification and preparation of final dataset

The pilot does not yet report a formal inter-coder reliability coefficient. For that reason, Table 5 reports only descriptive convergence across the main coding dimensions. These descriptive observations should not be interpreted as formal reliability estimates. Table 5 reports only descriptive convergence observed during the pilot coding process. It does not constitute a formal inter-rater reliability estimate. Because the present study is exploratory and based on a limited pilot sample (n = 36n = 36), formal reliability coefficients are pending future calculation. Future iterations of LEDAT should report Cohen’s kappa or Krippendorff’s alpha for each coded variable and, where appropriate, for the final interpretive band assignment. For continuous or quasi-continuous outputs such as LC, EC, and IDPAm, future work should also consider reporting intraclass correlation coefficients or equivalent agreement measures prior to reconciliation.

**Table 5.** Preliminary descriptive convergence across LEDAT coding dimensions.

Dimension	Preliminary convergence	Main source of discrepancy
Legal Component (LC)	Generally consistent	Offence classification and aggravating factors showed limited divergence
Ecological Component (EC)	Moderate convergence	Greater variability in reversibility and ecosystem sensitivity
Trigger activation	Exploratory and case-sensitive	Divergences concentrated in qualified ecological scenarios
Overall IDPAm band	Generally consistent	Most discrepancies concerned borderline cases between adjacent interpretive bands

The calculation of IDPAm follows the structure presented in Section 4. The Legal Component (LC) captures normative criminal seriousness through offence type (TP), intent (INT), aggravating factors (AGR), and recidivism or continuity (REC). The Ecological Component (EC) captures material ecological gravity through extent (EXT), ecosystem sensitivity or protection status (ECO), biodiversity impact (BIO), reversibility or duration (REV), substance type or intrinsic hazardousness (SUS), and concentration or volume (CONC). The final index is calculated as:

IDPAmbase=(Wj×LC)+(We×EC)

T=min(∑triggers,2)

IDPAm=IDPAmbase+T

where the default weights are Wj = 0.4 and We = 0.6. As explained above, the ordinary base scale ranges from 0 to 10, while the trigger adjustment may add up to +2 in qualified cases. The present pilot therefore assesses the operational feasibility of the IDPAm calculation, not full parameter calibration or external validation.

For reproducibility, the case-level dataset preserves the raw variable scores, standardised LC and EC values, trigger activations, capped trigger value, final IDPAm score, interpretive band, judicial outcome, court, year, sector, and procedural disposition where available. IDPAm was calculated using the unrounded standardised LC and EC values derived from the raw component scores. Rounded values are reported only for presentation purposes.

The Enforceability module (IDPAe) is not applied in this pilot phase. That omission is methodologically justified because public judgments rarely provide uniform or complete access to limitation issues, evidential nullities, expert disputes, chain-of-custody questions, attribution problems, conformity dynamics, or other procedural elements necessary for reliable IDPAe coding. The present empirical application therefore concerns only IDPAm. Future validation should incorporate IDPAe in order to explain divergence between material seriousness, procedural viability, and judicial outcome.

### 5.3 Descriptive results

The 36 coded cases generated IDPAm values ranging from 0.7 to 9.3, with a marked concentration in the lower and intermediate ranges of the scale. The mean IDPAm was 4.18, the median was 3.70, the standard deviation was 2.14, and the interquartile range extended from 3.00 to 5.78. This distribution is consistent with the article’s central premise: much environmental criminal litigation appears to operate within an uncertain zone situated between administrative unlawfulness and clearly serious criminal harm, rather than at the extremes of triviality or catastrophic ecological damage.

Using the indicative interpretive bands proposed by LEDAT, nineteen cases (52.8%) fall within the 0–3.9 range, corresponding to a predominantly administrative profile; thirteen cases (36.1%) fall within the 4.0–6.9 range, corresponding to the grey area of debatable criminal significance; and four cases (11.1%) reach the ≥7.0 range, corresponding to high material criminal significance. Consequently, 88.9% of the analysed cases fall below the high-significance range. This concentration should be interpreted descriptively rather than inferentially, given the pilot nature of the sample and its limited size.

**Figure 2.**
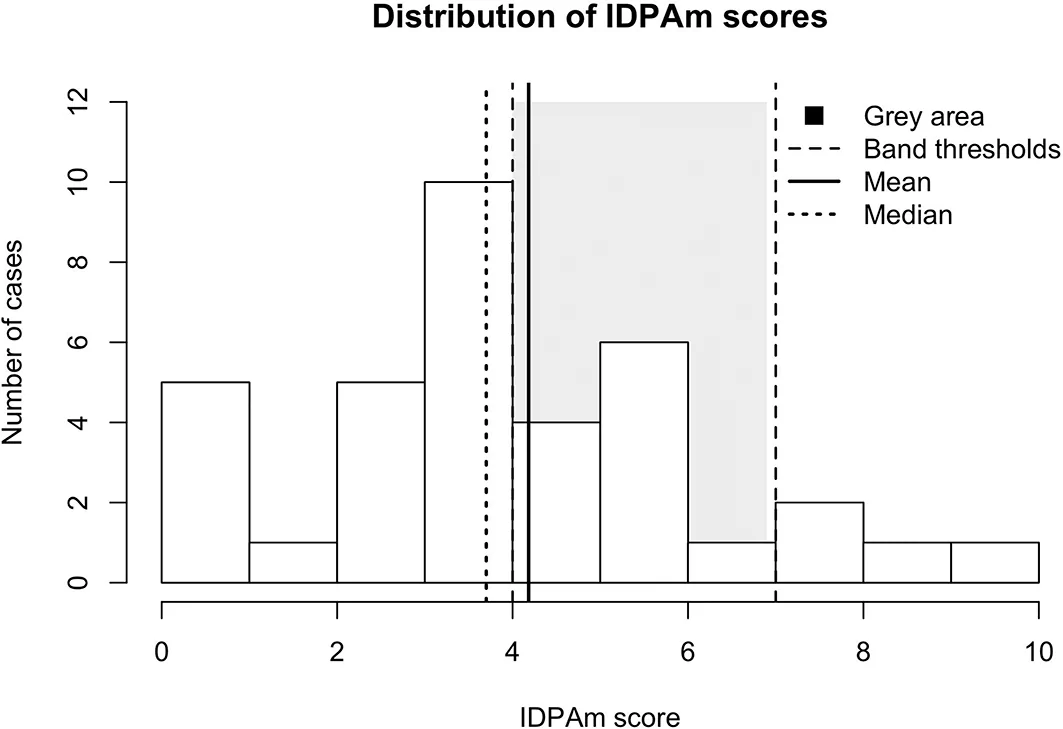
Distribution of IDPAm scores across the analysed sample (n = 36). The shaded area represents the interpretive grey zone proposed by the LEDAT model. Dashed vertical lines indicate the lower boundary of the grey zone and the high-significance threshold; solid and dotted vertical lines indicate the sample mean and median, respectively.

Figure 2 illustrates the overall distribution of IDPAm scores across the analysed sample. The histogram incorporates the indicative interpretive bands proposed by LEDAT and highlights the concentration of cases within the predominantly administrative and intermediate ranges of the index, particularly within the “grey area” corresponding to debatable criminal significance. Because of the small sample size and the likely non-normal distribution of IDPAm scores, the figure should be interpreted together with the median and interquartile range rather than relying exclusively on the mean.

This concentration should be interpreted descriptively rather than causally. It suggests that the practical utility of the model lies precisely in cases where judicial classification may be most unstable: situations involving intermediate gravity, moderate ecological damage, technically disputed impacts, or evidentially complex scenarios.

Within the pilot sample, cases ending in conviction displayed higher average IDPAm values than acquittals. Convictions had a mean IDPAm of 4.825, compared with 3.38 for acquittals. The corresponding medians were 4.50 and 3.50, respectively. This descriptive difference should not be interpreted as evidence of predictive capacity, causal influence, or statistical classification performance, but as an exploratory indication that the structured assessment of material seriousness tends to move in the same general direction as judicial outcomes in part of the sample.

However, the overlap between convictions and acquittals remains substantial. Moreover, eleven of the twenty convictions in the sample were resolved by conformity, which means that judicial outcome cannot be treated as a clean external validation criterion for IDPAm. Given the pilot nature of the sample, this comparison is reported descriptively only and is not treated as a test of predictive validity or statistical significance. Future applications should distinguish more systematically between acquittals, contested convictions, and conformity-based convictions, and should incorporate IDPAe in order to assess the procedural and evidential conditions that may explain divergence between material seriousness and judicial outcome.

**Figure 3.**
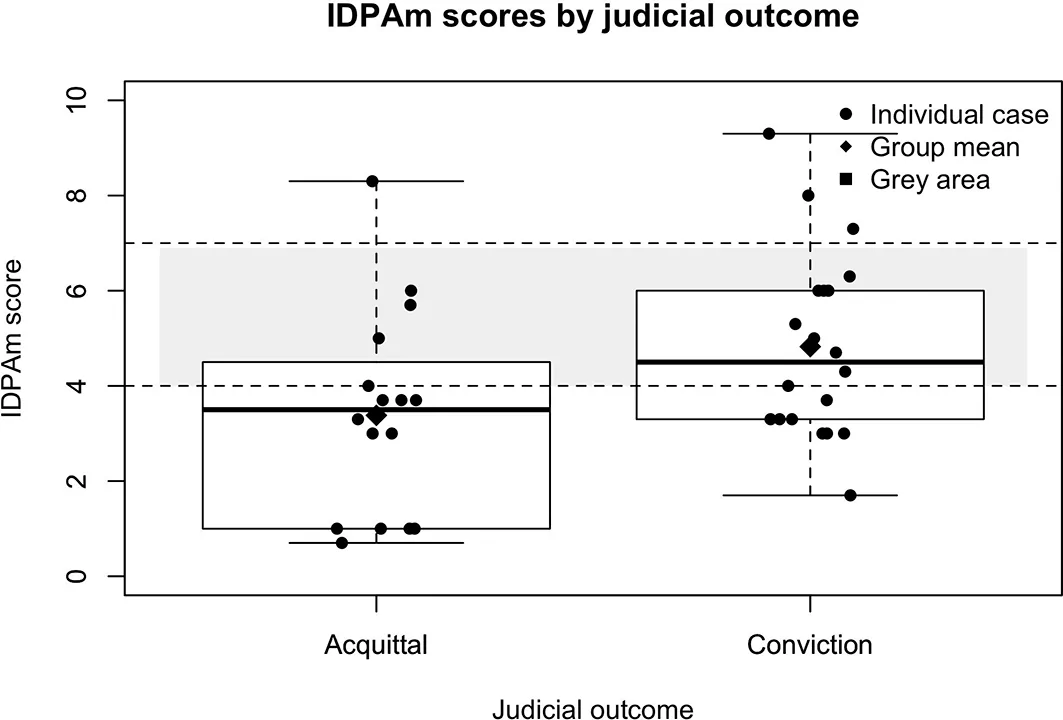
IDPAm scores by judicial outcome. The figure compares the distribution of IDPAm values in acquittals and convictions and illustrates the substantial overlap between both groups. The shaded area represents the interpretive grey zone. Points represent individual cases; diamonds indicate group means.

Figure 3 compares the distribution of IDPAm scores according to judicial outcome and illustrates the substantial overlap between convictions and acquittals.

### 5.4 Sectoral breakdown

The 36 valid cases are distributed across eleven sectoral labels in the coding database (see Table 6): noise pollution (contaminación acústica), noise pollution + injuries (contaminación acústica + lesiones), water dischargues (vertidos), waste (residuos), waste + emissions (residuos + emisiones), extractions (extracciones), emissions (emisiones), atmosferic pollution (contaminación atmosférica), damage to protected areas (daños a espacio protegido), natural resources (recursos naturales), and urban planing (urbanismo). These labels are retained in the descriptive table in order to preserve fidelity to the coded dataset. Because several sectors contain very small numbers of cases, the sectoral analysis is descriptive only and should not be interpreted as supporting sector-level inference.

**Table 6.** Sectoral distribution of the pilot sample and descriptive IDPAm values.

Material sector	n	Convictions	Mean IDPAm	SD	Range	Conviction rate (%)
Noise Pollution	12	6	2.63	1.38	0.7–5.0	50.0
Water discharges	6	3	5.40	1.80	3.0–8.0	50.0
Waste	4	4	5.65	1.54	3.7–7.3	100.0
Extractions	4	1	4.43	3.02	1.0–8.3	25.0
Air Pollution	2	1	7.65	2.33	6.0–9.3	50.0
Emissions	2	2	4.65	0.49	4.3–5.0	100.0
Noise Pollution + Injuries	2	2	2.35	0.92	1.7–3.0	100.0
Waste + Emissions	1	1	6.00	—	6.0–6.0	100.0
Urban Planning	1	0	4.00	—	4.0–4.0	0.0
Natural Resources	1	0	3.70	—	3.7–3.7	0.0
Damage to a protected area	1	0	3.30	—	3.3–3.3	0.0

In Table 6, sectoral means, standard deviations, ranges, and conviction rates are reported descriptively only. Standard deviations are not reported for sectors with a single observation. The sectoral labels reproduce the coding categories used in the pilot dataset and should not be interpreted as mutually exclusive legal categories or as supporting sector-level inference.

The largest sectoral group in the pilot corpus is noise pollution, with twelve cases and a mean IDPAm of 2.63. If the two cases coded as noise pollution + injuries are considered together with the acoustic-pollution group, noise-related cases account for fourteen of the thirty-six judgments. This indicates that noise-related litigation is prominent in the corpus, but those cases generally remain in the lower part of the IDPAm distribution. This result should not be generalised beyond the pilot sample, because it may reflect the evidential and factual configuration of the judgments selected rather than the inherent seriousness of noise pollution as an environmental harm.

Water discharges constitute the second largest group, with six cases and a mean IDPAm of 5.40. This places discharge cases within the interpretive grey zone and illustrates the methodological problem that LEDAT is intended to structure: ecologically relevant pollution cases often arise in precisely the range where criminal relevance is neither trivial nor self-evidently high. Waste cases also occupy an intermediate-to-high range, with a mean IDPAm of 5.65, while extractions show substantial dispersion, ranging from 1.0 to 8.3. Air pollution displays the highest sectoral mean, 7.65, but this figure is based on only two observations and should be treated with particular caution.

These patterns should not be overstated. The number of cases is too small for sectoral inference, particularly in sectors with one or two observations. Nonetheless, the sectoral breakdown is useful because it shows that LEDAT can be applied across heterogeneous forms of environmental offending while preserving the case-level variability of the underlying judgments. Its practical value may be greatest in domains such as discharges, waste, emissions, and extraction-related cases, where ecological seriousness is neither merely technical nor self-evidently catastrophic, and where the conventional legal threshold is especially unstable.

### 5.5 Interpretation of the pilot findings

The empirical application suggests three provisional findings.

First, the model appears to be operationally workable. The variables can be coded from publicly available judgments, and the resulting scores generate an internally plausible descriptive ordering. IDPAm values in the pilot corpus range from 0.7 to 9.3, which indicates that the model is capable of distinguishing lower, intermediate, and higher material-seriousness cases within heterogeneous judicial material. This is an important methodological point: the value of LEDAT lies not only in its abstract design, but in the fact that it can be applied to real case material in a way that supports structured comparison.

Secondly, the pilot data are consistent with the existence of a broad threshold zone between administrative and criminal unlawfulness. Most cases cluster below 7.0: nineteen cases fall within the predominantly administrative range, thirteen within the grey area, and four within the high material criminal significance band. In percentage terms, 52.8% of the sample falls within the predominantly administrative range, 36.1% within the grey area, and 11.1% within the high material criminal significance band. This distribution is consistent with long-standing doctrinal concerns about the indeterminacy of environmental criminal thresholds. The contribution of LEDAT is not to eliminate that indeterminacy, but to make the location of cases within it more transparent, auditable, and comparable.

Thirdly, the comparison between convictions and acquittals suggests that material seriousness and judicial outcome move in the same general direction in part of the sample, but not in a one-to-one fashion. Convictions show a higher mean IDPAm than acquittals: 4.825 compared with 3.38. The corresponding medians are 4.50 for convictions and 3.50 for acquittals. However, the distributions overlap substantially. Moreover, eleven of the twenty convictions in the corpus were resolved by conformity, which means that judicial outcome cannot be treated as a clean external validation criterion for IDPAm. This is precisely why the distinction between IDPAm and IDPAe matters. The present pilot does not yet model the procedural-evidential reasons why some materially more serious cases may fail, why some moderate cases may result in conviction, or how conformity-based outcomes affect the relationship between material seriousness and judicial result. That limitation should be recognised explicitly rather than obscured.

The present findings should therefore be interpreted exclusively in terms of descriptive coherence, methodological feasibility, and structured case ordering. The pilot application does not establish predictive validity, causal relationships, statistical classification performance, or inferential generalisability. LEDAT is proposed as an interpretative and organisational methodology rather than as a predictive adjudicative instrument.

In this respect, the future development of the model is also relevant to the broader European context. Directive (EU) 2024/1203 requires Member States to strengthen specialised training, coordination, national strategy, and statistical systems, and to transmit comparable data in a common format after the Commission establishes the standardised reporting framework. A structured methodology such as LEDAT may eventually prove useful within that wider movement toward more transparent and comparable environmental criminal enforcement, but only if its empirical reporting is equally rigorous and auditable.

The methodological ambition of LEDAT is therefore not to transform ecological criminal adjudication into a purely quantitative exercise, but to provide a structured and explainable composite framework capable of improving transparency, comparability, and argumentative coherence under conditions of legal and ecological complexity.

### 5.6 Sensitivity analysis of weighting structure and trigger adjustment

Given the formative and composite nature of LEDAT, the weighting structure should not be understood as a statistically fixed parameter but as a transparent modelling choice requiring exploratory assessment. Accordingly, a preliminary sensitivity analysis was conducted to evaluate whether plausible alternative weighting configurations produced substantial alterations in the relative ordering of cases or in their assignment to the proposed interpretive bands.

In addition to the default configuration (Wj = 0.4; We = 0.6)(Wj = 0.4; We = 0.6), two alternative scenarios were examined: balanced weighting (0.5/0.5)(0.5/0.5) and ecology-prioritised weighting (0.3/0.7)(0.3/0.7). The trigger system was also examined under a restricted scenario in which no trigger adjustment was applied. Alternative weighting scenarios were calculated using the same standardised LC and EC values and the same capped trigger adjustment, except in the no-trigger scenario, where TT was set to zero for all cases.

In the pilot dataset, the capped trigger value TT was derived from the available trigger variables T1–T4. The raw trigger sum was 0 in 12 cases, 1 in 14 cases, 2 in 5 cases, and 3 in 5 cases; accordingly, the cap affected 5 cases. Future versions of the coding manual should disaggregate the conceptual trigger categories more explicitly and document the evidential basis for each activation.

The exploratory comparison suggests that the overall ordinal distribution of cases remains highly stable across alternative weighting configurations. Compared with the default 0.4/ 0.6 structure, the balanced 0.5/ 0.5 configuration produced a Spearman rank correlation of 0.981 and a Kendall rank correlation of 0.936, with two cases changing interpretive band. The ecology-prioritised 0.3/0.7 configuration produced a Spearman rank correlation of 0.989 and a Kendall rank correlation of 0.950, also with two cases changing interpretive band. In both alternative weighting scenarios, the aggregate band distribution remained unchanged: nineteen cases in the predominantly administrative range, thirteen in the grey area, and four in the high material criminal significance band. The two band changes therefore concern case-level movement between adjacent categories, not a change in the overall distribution of cases across bands.

The removal of triggers produced a more visible effect. Although the ordinal association with the default configuration remained high, with a Spearman rank correlation of 0.940 and a Kendall rank correlation of 0.861, eight cases changed interpretive band. The distribution shifted from 19/ 13/4 under the default model to 24/ 11/1 under the no-trigger scenario. This indicates that the trigger mechanism has a substantive effect on classification in some cases, especially those involving qualified aggravating features. At the same time, the capped design prevents triggers from overwhelming the ordinary weighted matrix.

The pilot therefore suggests two exploratory conclusions. First, the relative weighting of the Legal Component and Ecological Component appears reasonably stable within the tested range of plausible alternatives. Secondly, the trigger mechanism is substantively consequential and should be documented at case level rather than treated as an auxiliary or merely illustrative element of the model. For empirical purposes, the sensitivity analysis supports the internal transparency of the pilot application: it shows how much of the final classification depends on ordinary weighting assumptions and how much depends on capped qualitative escalation (see Table 7). It does not, however, amount to statistical calibration or external validation of the model.

**Table 7.** Exploratory sensitivity check of weighting structure and trigger adjustment.

Configuration	Spearman correlation with default	Kendall correlation with default	Band changes	Band distribution	Main observed variation
0.4/0.6 default	1.000	1.000	0	19/13/4	Baseline
0.5/0.5	0.981	0.936	2	19/13/4	Minor movement in borderline cases
0.3/0.7	0.989	0.950	2	19/13/4	Minor movement in cases with divergent legal and ecological scores
No triggers	0.940	0.861	8	24/ 11/1	Reduced escalation in cases with qualified trigger activation

In Table 7 band distribution is reported as predominantly administrative/grey area/high material criminal significance. “Band changes” refers to the number of individual cases assigned to a different interpretive band relative to the default configuration. Because the present empirical application is exploratory and based on a limited sample (n = 36)(n = 36), these observations should not be interpreted as definitive statistical validation. Their function is more modest: to assess whether LEDAT remains reasonably stable under plausible alternative parameter configurations.

### 5.7 Limitations and next steps

The pilot nature of the empirical exercise should be stated clearly. The contribution of this study lies in methodological feasibility, structured case ordering, and transparency, not in statistical generalisation or predictive modelling. Several limitations therefore qualify the interpretation of the results.

The first limitation is sample size. A final valid sample of 36 judgments is sufficient for a pilot application, but not for robust inferential modelling. The descriptive patterns observed here are useful for methodological appraisal, yet they cannot support strong generalisations about Spanish environmental criminal enforcement as a whole. The sensitivity analysis is likewise exploratory and descriptive. The limited sample size prevents robust statistical calibration of weights or trigger effects, and future validation phases should incorporate larger comparative datasets and formal robustness metrics.

The second limitation is selection bias. The Tirant Prime search strategy is systematic, but still dependent on indexing practices, search terms, procedural classification, and the availability of written judgments. The resulting sample is therefore structured by the database architecture as well as by judicial practice. The corpus should accordingly be understood as a filtered jurisprudential sample rather than as a complete census of Spanish environmental criminal litigation between 2020 and 2025.

The third limitation concerns the procedural composition of the sample. The 36 judgments are first-instance decisions in the procedural sense, but they were issued by different trial courts depending on the applicable rules of jurisdiction: 26 by Provincial Courts and 10 by Criminal Courts. This is compatible with the design of the study, but it should be stated expressly to avoid equating “first instance” exclusively with Criminal Courts.

The fourth limitation is the absence of a fully reported inter-coder reliability coefficient. Because LEDAT is presented as a replicable coding methodology, the pilot should not rely solely on descriptive convergence after consensus. Cohen’s kappa or Krippendorff’s alpha should be calculated and reported in future applications, preferably at the level of individual variables and final interpretive bands. For continuous or quasi-continuous outputs such as LC, EC, and IDPAm, future work should also consider reporting intraclass correlation coefficients or equivalent agreement measures before reconciliation.

The fifth limitation is the non-inclusion of IDPAe. Without full case files, it is not possible to code limitation periods, evidential nullities, attribution problems, expert disputes, chain-of-custody issues, conformity dynamics, or other procedural obstacles in a uniform way. This means that the present pilot can describe the relation between material seriousness and judicial outcome only imperfectly. This limitation is particularly relevant because 11 of the 20 convictions in the corpus were resolved by conformity, and those outcomes cannot be treated as equivalent to fully contested convictions for validation purposes.

The sixth limitation is offence heterogeneity. The database includes noise-related cases, discharges, waste, waste-emission combinations, extractions, emissions, atmospheric pollution, protected-area damage, natural-resource cases, and urban planning offences. These categories differ substantially in evidential architecture, ecological dynamics, and normative framing. That heterogeneity is analytically interesting because it tests whether LEDAT can be applied across different types of environmental offending, but it also complicates direct comparison and prevents strong sector-level conclusions, especially where some sectors contain only one or two observations.

An additional limitation concerns the composite structure of the model itself. LEDAT is intentionally designed as a formative legal-ecological index integrating heterogeneous dimensions rather than as a reflective psychometric scale. Consequently, the model does not aim to demonstrate latent unidimensionality or internal consistency in the classical psychometric sense. Its future assessment should therefore focus on coding reliability, transparency of assumptions, sensitivity analysis, case-level traceability, and external coherence across expanded datasets rather than on psychometric criteria designed for reflective scales.

A further limitation concerns trigger documentation. The conceptual model defines a capped trigger system for qualified qualitative escalation, but empirical applications must record precisely which triggers are activated, why they are activated, and how the anti-duplication rule is applied. In the present pilot, the trigger mechanism has a meaningful effect on band classification: removing triggers changes the interpretive band in eight cases and reduces the number of high-significance cases from four to one. This confirms that triggers are methodologically important and should be documented at case level in future applications.

The present pilot does not demonstrate that LEDAT predicts judicial outcomes, nor does it seek to replace discretionary legal reasoning through algorithmic classification. Its contribution is methodological and organisational: to structure the assessment of ecological criminal seriousness in a more transparent, traceable, and contestable manner.

These limitations point directly to the next stage of research. Future work should expand the sample temporally and geographically; conduct formal inter-rater reliability testing; include the Enforceability module (IDPAe); distinguish acquittals, contested convictions, and conformity-based convictions; perform more detailed sensitivity analyses for the default weights and trigger treatment; and test the descriptive relationship between IDPAm, IDPAe, and judicial outcome using more systematic statistical tools where the data justify them. Cross-jurisdictional pilot application would be especially important if the model is to have broader European relevance after the transposition of Directive (EU) 2024/1203 on 21 May 2026 and the later development of comparable Union-wide statistical reporting.

For reproducibility, a case-level coding annex will be made available as supplementary material upon publication, subject to the limitations described in the Data Availability Statement and Code Availability Statement. At minimum, that annex should list: case identifier, court, year or date, material sector, procedural disposition where available, TP, INT, AGR, REC, EXT, ECO, BIO, REV, SUS, CONC, triggers activated, LC, EC, IDPAm, interpretive band, judicial outcome, and notes on evidential uncertainty. If licensing, confidentiality, or editorial limits prevent full publication of any component, the annex should preserve the maximum level of derived coding information compatible with those constraints. This addition is central to the article’s claim that LEDAT offers a transparent and auditable methodology.

## 6. Implications for enforcement, remedies, and empirical monitoring

LEDAT is designed as a transparent argumentative aid, not as a binding metric. Its function is to structure the assessment of ecological criminal seriousness in a way that can be explained, reviewed, challenged, and reproduced. In line with Directive (EU) 2024/1203, which requires adequate resources, specialised training, coordination, national strategies, and comparable statistics, LEDAT may assist prosecutors, experts, courts, and defence counsel only if its operation remains fully traceable and open to contradiction.

The practical use of LEDAT must therefore respect three limits. First, the output of the model cannot replace legal classification, evidential assessment, or judicial reasoning. Secondly, IDPAm and IDPAe must remain analytically separate: IDPAm concerns material gravity, whereas IDPAe concerns procedural and evidential viability. Thirdly, any use of LEDAT in criminal proceedings must be accompanied by a source-by-variable explanation, identifying the factual and evidential basis for each score. A final IDPAm value without a transparent coding trail would be methodologically insufficient and legally unacceptable.

Traceability is particularly important because environmental criminal cases often depend on complex expert evidence. LEDAT should therefore be used, where appropriate, as a structured format for organising expert reasoning rather than as a substitute for that reasoning. Each assigned value should be linked to a specific evidential source, such as expert reports, environmental inspections, administrative files, laboratory results, judicial findings, maps, protected-area classifications, species conservation status, or toxicological data. Where the evidence is incomplete, uncertain, or contested, that uncertainty should be expressly recorded rather than hidden behind the numerical output.

The same logic applies to remedies. LEDAT may help structure reasoning on restoration, compensation, remediation, and ancillary measures, but it cannot mechanically determine them. Where damage is reversible, the ecological variables may assist in identifying the intensity, duration, and territorial scope of restoration needs. Where damage is irreversible or only partially reversible, the model may support a more transparent discussion of compensation, substitute restoration, or ecosystem-service loss. However, remedial decisions must remain governed by the applicable legal framework, the evidence in the case, and the principle of proportionality.

Nor should LEDAT be understood as a sentencing grid. A high IDPAm score may indicate high material ecological seriousness, but it does not by itself determine the penalty. Sentencing requires additional legal reasoning concerning culpability, participation, aggravating and mitigating circumstances, proportionality, deterrence, reparation, and the statutory penalty framework. LEDAT can support that reasoning by making the ecological and legal seriousness of the case more explicit, but it cannot replace the court’s individualised assessment.

The model may also be relevant to ancillary measures such as permit withdrawal, disqualification, compliance obligations, confiscation-linked remediation, or corporate environmental monitoring schemes. In these contexts, LEDAT can help identify why a case is materially serious, which ecological dimensions are most affected, and what type of institutional response may be proportionate. Yet those measures should never be imposed mechanically on the basis of the index alone. Their justification must remain legal, evidential, and case-specific.

For these reasons, any use of LEDAT in judgments, prosecutorial files, expert reports, or defence submissions should comply with minimum procedural safeguards: use of a coding manual; identification of the evidential source for each variable; a short justification for each score; explicit explanation of any trigger activation; application of the anti-duplication rule; preservation of an audit trail; and access for the parties to examine and challenge the coding. These safeguards are especially important because the pilot application shows that trigger activation can affect interpretive band classification in some cases, and because conformity-based outcomes cannot be treated as equivalent to fully contested adjudication. Black-box use is incompatible with the legal nature of the model.

The point of LEDAT is not to automate the process of enforcing environmental laws, but to improve the quality of legal reasoning when it comes to complex ecological and evidential issues. When used correctly, this model can show the factors that are often not considered in threshold reasoning. These include how serious the ecological harm or risk is, the legal relevance of the behaviour, how strong the evidence in the case is, the way the problem is being solved, and the relationship between how serious the problem is, how it can be enforced, the remedies and penalties. For it to be seen as legitimate, it must be transparent, not binding, open to debate and subject to judicial review, as shown in Table 8.

**Table 8.** Procedural uses and limits of LEDAT in enforcement, remedies, sentencing, and empirical monitoring.

Procedural use	LEDAT contribution	Limit
Prosecutorial assessment	Structures ecological seriousness and threshold reasoning	Does not determine whether charges must be filed
Expert report	Organises ecological variables and evidential sources	Does not replace expert judgment
Judicial reasoning	Makes explicit the basis of material gravity assessment	Does not bind judicial discretion
Defence challenge	Allows variable-by-variable contradiction	Does not reverse the burden of proof
Remedies	Supports proportional restoration or compensation reasoning	Does not mechanically determine reparation
Sentencing	Informs seriousness and proportionality analysis	Is not a sentencing grid
Empirical monitoring	Supports descriptive comparison across cases, sectors, and outcomes	Does not establish predictive validity, causal effects, or inferential generalisability

## 7. Conclusion

Environmental criminal law increasingly depends on the ability to distinguish administrative unlawfulness from criminally relevant ecological harm or risk. Yet that distinction continues to be mediated through open-textured threshold concepts such as “substantial damage,” “serious harm,” and “serious risk.” Directive (EU) 2024/1203 strengthens the European framework by expanding offences, sanctions, enforcement obligations, and harm-related assessment criteria, but it does not eliminate the practical difficulty of operationalising ecological seriousness in concrete cases. The central problem is therefore not the existence of judicial discretion as such, but the absence of transparent methodologies capable of structuring that discretion in an auditable and empirically observable way.

This article has proposed LEDAT as a structured legal-ecological coding framework designed to address that problem. Its contribution lies in organising legally relevant ecological and normative variables into a transparent framework capable of supporting threshold reasoning without replacing it. The Material Environmental Criminal Damage Index (IDPAm) structures the assessment of material seriousness through the integration of a Legal Component, an Ecological Component, and a capped trigger system for qualified qualitative escalation. At the same time, the Enforceability module (IDPAe) preserves the necessary distinction between substantive gravity and procedural or evidential viability. This distinction is essential: a case may involve materially serious ecological harm yet fail evidentially or procedurally, just as a procedurally robust case may involve only moderate ecological seriousness.

The pilot application to 36 first-instance Spanish environmental crime judgments issued by Criminal Courts and Provincial Courts acting as trial courts suggests that LEDAT can be operationalised in real case material and that its outputs provide an internally plausible descriptive ordering of cases. IDPAm values range from 0.7 to 9.3, with a mean of 4.18 and a median of 3.70. Most cases in the pilot sample clustered within the predominantly administrative or intermediate grey-area bands: nineteen cases fell within the 0–3.9 range, thirteen within the 4.0–6.9 grey area, and four within the high material criminal significance band. In percentage terms, this corresponds to 52.8%, 36.1%, and 11.1% of the sample, respectively. This finding is consistent with the premise that environmental criminal law often operates in a zone of threshold uncertainty, where ecological seriousness is neither trivial nor self-evidently catastrophic.

The comparison between convictions and acquittals also suggests that higher IDPAm values tend descriptively to coincide with convictions in part of the sample. Convictions displayed a higher mean IDPAm than acquittals, 4.825 compared with 3.38, while the corresponding medians were 4.50 and 3.50. However, the overlap between both groups was substantial. Moreover, eleven of the twenty convictions were resolved by conformity, which confirms that judicial outcome cannot be treated as a clean external validation criterion for IDPAm. This reinforces the importance of distinguishing material seriousness from procedural disposition, evidential viability, and negotiated or conformity-based outcomes.

The empirical claims advanced here are necessarily limited. The pilot does not establish predictive validity, causal relationships, statistical classification performance, or inferential generalisability. Nor does it provide full validation of the model. Its purpose is more modest: to test whether LEDAT can be applied coherently, transparently, and contestably to a small corpus of real judgments. The absence of a formal inter-coder reliability coefficient, the limited sample size, the non-inclusion of IDPAe, the presence of conformity-based convictions, and the heterogeneity of environmental offence types all require caution. Future research should therefore expand the dataset, report formal reliability measures, conduct more detailed sensitivity analyses, include IDPAe systematically, distinguish contested convictions from conformity-based outcomes, and test the model across different jurisdictions and offence sectors.

The broader contribution of the article is methodological rather than predictive. LEDAT does not assume that ecological criminal seriousness can be reduced exhaustively to numerical representation. Its premise is more restrained: structured approximation may improve the transparency, consistency, and traceability of legal reasoning under conditions of ecological and evidential complexity. Properly understood, the model does not create new offences, determine criminal liability, fix penalties, or displace judicial discretion. It organises factors that are already relevant to environmental criminal assessment and makes their use more explicit, auditable, and open to challenge.

For empirical legal studies, the significance of LEDAT lies in making environmental criminal thresholds more observable. The framework translates heterogeneous legal and ecological considerations into variables that can be coded, compared, audited, and contested across cases. In the post-2024 European framework, this kind of methodological development is particularly important. Directive (EU) 2024/1203 requires Member States to strengthen resources, training, coordination, national strategies, and statistical comparability in environmental criminal enforcement. A structured legal-ecological methodology such as LEDAT may contribute to that broader movement by helping courts, prosecutors, experts, defence counsel, and researchers reason more transparently about ecological seriousness.

Its legitimacy, however, depends on remaining non-binding, methodologically restrained, contestable, and subordinate to the constitutional safeguards of criminal adjudication. Framed in those terms, LEDAT offers not a mechanical solution to environmental criminal thresholds, but a structured bridge between legal indeterminacy, ecological complexity, and empirical observability.

## Code availability statement

The R scripts used to generate the descriptive statistics, figures, consistency checks, and sensitivity analyses are available as Supporting Information. The scripts reproduce the analyses reported in the article from the derived case-level coding dataset. They are provided for transparency, verification, and scholarly replication of the pilot results.

## Ethics statement

This study does not involve interviews, surveys, experiments, animal subjects, or interaction with human participants. The empirical material consists of judicial decisions and derived coding variables. No personal data beyond information contained in judicial decisions was independently collected. The analysis is reported at aggregate and case-coded level, and the supplementary dataset avoids unnecessary personal identifiers. For these reasons, formal ethics committee approval was not required.

## Artificial intelligence and language editing disclosure

The author used DeepL Write/Translator to assist with English-language translation, grammar, and stylistic refinement, as English is not the author’s native language. The tool was not used to generate research data, conduct analysis, produce results, or determine the article’s arguments or conclusions. All AI-assisted language suggestions were reviewed, revised, and approved by the author, who takes full responsibility for the content of the manuscript.

## Data Availability

The coded case-level dataset and all replication materials underlying the analyses reported in this article are openly available in Zenodo under a Creative Commons Attribution 4.0 International licence (CC-BY 4.0): Morelle-Hungría, E. (2026). *Dataset and replication materials: Ecological harm and criminal thresholds – A pilot legal-ecological coding framework for environmental crime judgments (LEDAT, Spain 2020–2025).* Zenodo. https://doi.org/10.5281/zenodo.20524581 The deposit includes the full case-level coding dataset (n = 36) with all legal and ecological variables, derived IDPAm scores, band classifications, judicial outcomes, summary tables underlying all reported statistics and figures, and the R analysis script. The full text of the judgments is not included due to database licensing restrictions (Tirant Prime); all judgments are publicly accessible through official Spanish legal databases. No datasets are under embargo.

## Footnotes

^1^
This article forms part of the research results obtained within the project *“Análisis criminológico de la eficacia de la política criminal medioambiental frente a la emergencia climática – CRIMICLIMA”* (Reference: UJI-2023-02), hosted by the Department of Public Law at Universitat Jaume I and developed during the period 1 January 2024–31 December 2026. This article forms part of the research results obtained within the project ‘Análisis criminológico de la eficacia de la política criminal medioambiental frente a la emergencia climática – CRIMICLIMA’ (Reference: UJI-2023-02), hosted by the Department of Public Law at Universitat Jaume I (1 January 2024–31 December 2026). It also forms part of the project ‘Ocean Crime Narratives: A Polyhedral Assessment of Hegemonic Discourse on Environmental Crime and Harm at Sea (1982-present) – OCN’, funded by the European Commission under Horizon Europe (Grant Agreement No. 101043711), Principal Investigator: Dra. Marta Puxan-Oliva (1 October 2022–30 September 2027). The author participates in the OCN project as a collaborating researcher.
