# Ecological harm and criminal thresholds: A pilot legal-ecological coding framework for environmental crime judgments

**DOI:** 10.12688/openreseurope.24170.2

**Published:** 2026-09-17

**Authors:** Esteban Morelle-Hungría

**Affiliations:** 1 Public Law, University Jaume I Faculty of Law and Economics, Castellón de la Plana, Valencian Community, 12071, Spain

**Keywords:** empirical legal studies, environmental crime, ecological harm, criminal thresholds, judicial decisions, legal coding, composite index

## Abstract

Environmental criminal law frequently relies on open-textured threshold concepts such as “substantial damage, “ “serious harm, “ and “serious risk” to distinguish administrative unlawfulness from criminally relevant conduct. Yet courts often apply these thresholds without a reproducible framework for structuring ecological seriousness across cases. This article introduces LEDAT ® (Legal-Ecological Damage Assessment Tool), a structured coding framework that combines legal and ecological variables into a Material Environmental Criminal Damage Index (IDPAm), while distinguishing material seriousness from procedural and evidential enforceability. IDPAm measures the material legal-ecological seriousness of a case as structured by the framework; it does not measure criminal liability, ecological damage alone, or the likelihood of conviction. The framework is piloted on 36 Spanish first-instance environmental crime judgments issued between 2020 and 2025, identified through a systematic search of a national jurisprudential database followed by qualitative screening. Cases were independently coded by two trained researchers using a shared coding manual and reconciled after comparison; pending formal agreement measures, coding convergence is reported as descriptive. IDPAm values ranged from 0.7 to 9.3. Most cases fell within predominantly administrative or intermediate grey-area bands, while four reached high material criminal significance. Convictions showed higher descriptive IDPAm values than acquittals, although distributions overlapped substantially and eleven of twenty convictions were conformity-based. Sensitivity checks indicate high ordinal stability under alternative legal/ecological weights, while trigger removal affects band classification. The article makes no predictive, causal, or inferential claims. Its contribution is methodological: LEDAT offers a transparent, contestable, and reproducible framework for empirical analysis of environmental criminal thresholds.

## 1. Introduction

Environmental criminal ^1^ law in the European Union continues to rely on open-textured threshold concepts such as “substantial damage,” “serious harm,” and “serious risk” to distinguish administrative unlawfulness from criminally relevant conduct. These concepts perform an essential legal function: they mark the point at which regulatory non-compliance becomes sufficiently serious to justify criminal intervention. Yet they also create a recurring problem for empirical legal analysis. Although Directive (EU) 2024/1203 significantly strengthens the European framework by expanding offences, sanctions, and enforcement obligations, it does not provide an operational methodology for structuring the assessment of ecological seriousness across cases and jurisdictions. Legally decisive threshold concepts therefore remain methodologically under-specified, generating interpretative variability in the treatment of materially similar cases ( Fuentes Osorio, 2019, 2021).

This problem is not merely doctrinal. It is also empirical and evidential. Environmental crime poses particular challenges for criminal law because the harms involved are frequently cumulative, multidimensional, and difficult to delimit within conventional legal categories. Such harms may affect individuals, communities, species, and ecosystems simultaneously, often extending beyond immediate or localised degradation and producing broader forms of social harm that exceed traditional models of individualised victimisation (Council of the European Union, 2023; Ruiz Arias, 2021). At the same time, environmental offences commonly depend on complex scientific evidence, since ecological systems are dynamic, adaptive, and rarely susceptible to simple linear attribution. The evidential difficulties associated with ecological harm are further intensified by institutional limitations in specialist expertise and investigative capacity ( Baucells, 2024).

These challenges arise within a broader context of ecological crisis and legal transformation. European environmental policy increasingly operates under conditions of escalating ecological pressure and political tension regarding the scope and intensity of environmental protection ( Fajardo del Castillo, 2023). From the ecocentric perspective adopted in this article, the relevant protected legal interest cannot be reduced to a merely administrative conception of “the environment”; rather, the ecosystem itself must be understood as a legal interest capable of autonomous criminal-law relevance ( Morelle-Hungría, 2024). This perspective is especially relevant following Directive (EU) 2024/1203, which replaces Directive 2008/99/EC and reinforces the role of criminal law within environmental governance. The recast Directive expands the catalogue of offences, strengthens sanctions for both natural and legal persons, and obliges Member States to improve training, coordination, resources, and enforcement capacity. At the same time, however, it continues to rely on evaluative threshold expressions—such as “substantial damage,” “widespread damage,” “irreversible or long-lasting damage,” and “non-negligible quantities”—whose practical application still depends on national interpretation, expert evidence, and judicial assessment.

The Directive is therefore relevant not only as a legal instrument, but also as a source of a measurement problem. First, it confirms that environmental criminal liability cannot be reduced to the formal breach of administrative standards alone, but requires an additional assessment of seriousness. Secondly, although the Directive identifies relevant assessment factors—including the baseline condition of the affected environment, the duration and reversibility of the damage, its extent, and the exceedance of regulatory thresholds—it does not itself provide a reproducible coding framework capable of organising those criteria within prosecutorial, expert, or judicial practice. Judicial discretion consequently remains unavoidable. Such discretion is not objectionable in itself; comparative scholarship has warned against excessively rigid decision-guidance models that could unduly constrain judicial autonomy ( Poppleton et al., 2021). The difficulty arises when the assessment of ecological seriousness remains implicit, fragmented, and methodologically opaque.

The Spanish legal system provides a useful empirical setting in which to examine this broader problem. Under Articles 325–327 of the Spanish Criminal Code, courts must frequently determine whether an unlawful emission, discharge, extraction, or ecological disturbance remains within the sphere of administrative unlawfulness or instead reaches the additional gravity required for criminal punishment. In practice, this determination often depends on technical assessments concerning ecological impact, risk, duration, reversibility, ecosystem sensitivity, pollutant concentration, or protected species and habitats. Spain is therefore not treated here as an isolated doctrinal case study, but as a jurisdictional context in which a general empirical legal problem can be observed: how courts reason about environmental criminal thresholds when the underlying harm is ecologically complex and evidentially mediated.

This article addresses that problem by proposing LEDAT (Legal-Ecological Damage Assessment Tool), a structured legal-ecological coding framework designed to organise the assessment of material environmental criminal harm in a transparent, traceable, and contestable manner. LEDAT combines a Legal Component and an Ecological Component into a synthetic indicator—the Material Environmental Criminal Damage Index (IDPAm)—while distinguishing that substantive assessment from a separate Enforceability module (IDPAe), which concerns procedural and evidential viability. This distinction is central to the model’s logic. Materially serious ecological harm may fail procedurally, just as evidential sufficiency does not itself determine substantive ecological gravity. The framework therefore separates the empirical coding of material seriousness from the procedural question of whether a criminal case can be successfully sustained.

It is important to define the scope of IDPAm precisely. IDPAm is not a direct biophysical measure of environmental damage, nor a measure of ecological harm considered independently of its legal context: it is an integrated indicator of material criminal seriousness. The Ecological Component records structured dimensions of ecological gravity or risk, whereas the Legal Component records the normative criminal configuration of the case, including offence classification, intent, aggravating circumstances, and recidivism or continuity. Cases involving comparable ecological harm may therefore receive different IDPAm scores where their legal characteristics differ. This is a deliberate design feature: LEDAT is intended to make explicit the interaction between ecological materiality and legally relevant features of criminal seriousness, not to collapse them into a purely ecological metric. In particular, a higher IDPAm score indicates greater material legal-ecological seriousness as structured by the framework; it does not imply criminal liability, a higher probability of conviction, or a more severe penalty.

The architecture of LEDAT is intentionally restrained. The weighting assigned to the Legal Component (Wj = 0.4) and the Ecological Component (We = 0.6) reflects the fact that the index is designed primarily to structure material ecological seriousness while preserving a normative legal filter so that criminal relevance is not reduced to ecological measurement alone. The index should be understood as a formative composite framework: its variables are not interchangeable manifestations of a single latent construct, but distinct legal and ecological indicators organised for structured comparison. Because those variables are ordinal, the resulting scores should be read as structured ordinal-comparative approximations rather than metrically exact measurements. Likewise, the trigger mechanism is capped at a maximum of +2 in order to capture qualified qualitative escalations—such as critically endangered species, maximum-protection ecosystems, or grave risks to human health—without allowing those factors to overwhelm the base matrix or generate disproportionate inflationary effects. LEDAT is therefore not conceived as an automated decision-making system, a sentencing grid, or a substitute for judicial reasoning. Its purpose is methodological: to make explicit, structured, and contestable the factors that already underlie threshold reasoning in environmental criminal law.

The interpretive bands proposed by LEDAT are also deliberately provisional. The categories of 0–3.9, 4–6.9, and ≥ 7 are heuristic devices for organising descriptive comparison among cases; they do not establish legal thresholds or determine criminal liability. In particular, the expression “predominantly administrative” refers only to a lower material-criminal-significance profile generated by the index; it does not mean that a case was, or should have been, resolved through administrative rather than criminal proceedings. Because ecological harm is continuous rather than naturally divided into discrete categories, cases close to the proposed boundaries require particular interpretative caution, and the empirical analysis therefore examines the sensitivity of classifications to plausible alternative weighting structures, trigger configurations, and band boundaries.

The article does not claim definitive validation of the model. Instead, it presents a pilot empirical application of LEDAT to a sample of 36 first-instance Spanish environmental crime judgments issued between 2020 and 2025 by Criminal Courts and Provincial Courts acting as trial courts according to the applicable procedural rules, identified through a systematic search of a national jurisprudential database (1,145 records retrieved, 157 first-instance judgments, 36 retained after qualitative screening). The cases were independently coded by two trained junior researchers using a shared coding manual and an independent parallel coding protocol, in which each coder worked separately and without access to the other coder’s classifications before reconciliation. Because formal agreement coefficients have not yet been calculated from the pre-reconciliation codings, the coding convergence reported here is descriptive and provisional. The empirical objective is exploratory: to assess the initial coherence, usability, and descriptive potential of the methodology in real case material. The limitations of the pilot—including sample size, selection effects, heterogeneity across offence sectors, incomplete procedural information, and the presence of conformity-based convictions—are considered when interpreting the results.

The article makes three contributions to empirical legal studies. First, it reframes environmental criminal thresholds as a problem of legal-ecological coding and measurement, rather than solely as a problem of doctrinal interpretation. Secondly, it introduces a transparent and reproducible framework for organising heterogeneous legal and ecological variables in environmental crime judgments. Thirdly, it provides a pilot empirical application showing how structured coding may improve the comparability and contestability of threshold assessment without displacing judicial discretion. More broadly, the article argues that structured legal-ecological methodologies may contribute to greater transparency, consistency, and comparability in the enforcement of environmental criminal law within the post-2024 European framework, provided that their use remains non-binding, methodologically transparent, and fully compatible with the constitutional safeguards governing criminal adjudication. That contribution also remains conditional in a methodological sense: it depends on explicit coding rules, transparent handling of non-applicable, missing, insufficient, and contested information, preservation of the evidential basis for each assigned value, formal inter-coder reliability assessment, documented trigger activation, sensitivity analysis, and cautious interpretation of judicial outcomes. Under those conditions, LEDAT is proposed not as a definitive solution to the indeterminacy of environmental criminal thresholds, but as a structured and auditable framework for investigating how those thresholds are assessed in practice.

## 2. The problem of “substantial harm”

The central difficulty addressed in this section is that “substantial harm” performs an indispensable threshold function in environmental criminal law while remaining difficult to apply in a stable and transparent manner. It marks the point at which conduct that is already unlawful in administrative terms may acquire the additional seriousness required for criminal intervention. Yet it does so through open-textured evaluative language that may be interpreted differently across cases. For empirical legal analysis, this creates a problem of structured observation: courts, prosecutors, experts, and researchers must assess ecological seriousness, but the legal, ecological, and evidential considerations informing that assessment are often implicit, fragmented, or unevenly documented in judicial decisions.

The difficulty has three interconnected dimensions. First, threshold clauses generate legal uncertainty because they distinguish administrative unlawfulness from criminal relevance through evaluative expressions such as “substantial damage,” “serious harm,” and “serious risk.” Secondly, the scientific assessment of ecological damage is empirically demanding, particularly where effects are cumulative, delayed, multi-causal, spatially dispersed, or only probabilistically foreseeable. Thirdly, the practical assessment of seriousness is conditioned by evidential limitations: the available judicial record may provide incomplete, contested, or unevenly detailed information concerning ecological impact, causation, duration, reversibility, hazardousness, or ecosystem sensitivity. The issue is therefore not only how environmental criminal law should define “substantial harm,” but also how the factors relevant to that assessment can be identified, documented, and critically examined without treating ecological complexity as if it could be reduced to a single, legally determinative numerical value.

Directive (EU) 2024/1203 improves the European framework by identifying factors relevant to substantial damage, likely damage, and non-negligible quantity. It thereby provides a more detailed legal orientation for assessing ecological seriousness than the previous framework. However, it does not establish a fully operational methodology for translating those factors into everyday prosecutorial, expert, judicial, or empirical research practice. The argument advanced here is not that quantification can eliminate discretion, resolve evidential uncertainty, or transform ecological seriousness into a fixed legal rule. Rather, it is that structured, auditable, and contestable coding criteria may make the assumptions underlying threshold reasoning more visible. This need is particularly acute in intermediate cases. At the extremes, ecological seriousness may be comparatively clear: some cases involve negligible or technical infringements, whereas others involve extensive, irreversible, or otherwise manifestly serious damage. Between those poles lies a more difficult area in which the criminal relevance of the conduct is neither self-evidently absent nor self-evidently established. In that area, assessments may depend on the interaction of legal classification, culpability, aggravating factors, spatial extent, duration, reversibility, hazardousness, ecological sensitivity, biodiversity impact, and the quality of the available evidence. A legal-ecological coding framework cannot determine the correct legal outcome of such cases. It can, however, provide a disciplined way to record, compare, and scrutinise the factors that structure the assessment of material criminal seriousness.

### 2.1 Legal uncertainty and threshold clauses

In environmental criminal law, the notion of “substantial harm” fulfils a threshold function: it marks the point at which conduct that is already unlawful in administrative terms acquires the additional gravity required for criminal intervention. That function is neither incidental nor secondary. In systems such as the Spanish one, it is constitutive of the dual sanctioning model, because environmental offences frequently combine a prior breach of environmental regulation with a further clause of criminal significance indicating when the conduct becomes punishable as a crime. As Fuentes Osorio has argued, these clauses are intended to identify the harmful potential that transforms an administrative infringement into a criminal offence, but they do so through open-ended and indeterminate concepts that do not always provide the degree of certainty required by the principle of specificity in criminal law ( Fuentes Osorio, 2019). This has contributed to broader doubts about the practical effectiveness of the dual protection model and, in the Spanish debate, to the claim that criminal environmental protection may in some cases be less effective than administrative protection in remediating ecological damage ( Cuerda Arnau, 2023; Fuentes Osorio, 2023).

The difficulty is not simply lexical. “Substantial harm,” “substantial damage,” “serious harm,” and “serious risk” are threshold expressions that perform a normative sorting function. They are intended to prevent the excessive extension of criminal law, while also identifying the additional seriousness that may justify criminal intervention. Yet they create a zone of interpretative uncertainty in which cases with comparable material features may be classified differently. For empirical legal studies, this is not only a problem of legal doctrine; it is also a problem of structured observation. Unless the factors that move a case from administrative unlawfulness to criminal significance are identified and documented transparently, it is difficult to compare cases, assess consistency, or evaluate how courts operationalise ecological seriousness in practice. The relevant question is not whether discretion can be eliminated, but whether the exercise of discretion can be made sufficiently explicit to permit scrutiny, disagreement, and reasoned comparison.

The doctrinal importance of this problem has increased with the revision of the European framework. Directive 2008/99/EC referred to “substantial damage” without defining the concept, a feature that attracted sustained criticism for its vagueness and for the practical difficulties it created in ecological contexts, especially in aquatic environments ( Rebelo et al., 2024, p. 423). Directive (EU) 2024/1203 improves that position by identifying relevant assessment factors rather than by creating a quantified legal test. Article 3(6) requires Member States to take into account, where relevant, the baseline condition of the affected environment, the temporal profile of the damage, its extent, and its reversibility when assessing whether damage or likely damage is “substantial.” Article 3(7) adds factors relevant to the assessment of likely damage, including the hazardousness of the activity or substance and the extent to which regulatory thresholds have been exceeded; Article 3(8) does the same for negligible and non-negligible quantities. Recital 25 confirms that these are non-exhaustive elements intended to support coherent application, while Recital 46 stresses that Member States should define the scope of administrative and criminal enforcement clearly.

The post-2024 European framework should therefore be understood as an improvement in legal orientation rather than a full solution to the threshold problem. The Directive narrows the field of discretion by identifying factors that competent authorities must consider, and it strengthens the broader enforcement architecture through obligations relating to resources, training, national strategies, and statistical data. Yet it deliberately leaves room for national transposition and case-specific judicial assessment. This is not objectionable in itself. Comparative literature has rightly warned that highly prescriptive models can undermine judicial autonomy and distort penal proportionality ( Poppleton et al., 2021). The methodological challenge is therefore not to substitute a numerical device for legal judgment. It is to develop tools that disclose the legal, ecological, and evidential premises on which an assessment of seriousness rests. In their absence, discretion is not merely preserved; the grounds on which it has been exercised become difficult to observe, compare, or evaluate.

The Spanish doctrinal debate reinforces this concern. Morelle-Hungría argues that the current structure of environmental offences remains affected by the insufficient integration of scientific knowledge into the offence definition itself, a weakness already identified in earlier work on the difficulty of clarifying the protected legal interest and the level of gravity required by environmental offences ( Morelle-Hungría, 2024; Morelle-Hungría, 2022a). From the ecocentric perspective adopted here, the ecosystem should not be treated merely as a regulatory backdrop but as the substantive core of protection, allowing legal analysis to incorporate system-level ecological values. Eco-criminological arguments reinforce the point: anthropogenic pressures have reached levels that exceed quantifiable planetary boundaries, exposing the limits of legal models that assess ecological wrongdoing solely through traditional categories of individualised injury or abstract risk ( Morelle-Hungría, 2022b). The consequence is not that criminal law should become a technocratic exercise or that ecological seriousness should be treated as a self-executing numerical fact. Rather, evaluative legal clauses require more explicit methodological mediation if they are to remain compatible with legality, proportionality, evidential rationality, and the individualised assessment required in criminal adjudication.

From the standpoint of LEDAT, the implication is limited but concrete. The model is not presented as a substitute for legal interpretation, a measure of criminal liability, or a mechanism for converting threshold clauses into fixed numerical rules. It is proposed as a disciplined means of structuring the assessment that threshold clauses already require. Its usefulness depends on whether the criteria identified by Directive (EU) 2024/1203 can be translated into transparent legal and ecological variables capable of supporting reasoned, auditable, and contestable analysis. That translation must remain methodological rather than determinative. A structured index may assist in identifying which material features of a case warrant closer legal analysis, but it cannot establish the elements of an offence, prove causation or culpability, determine evidential sufficiency, predict a conviction, or fix a sentence. Those questions remain governed by the applicable law, the evidence, and judicial assessment in the individual case. Without transparent mapping, however, the empirical study of environmental criminal thresholds remains limited because the factors that structure judicial classification are insufficiently observable.

### 2.2 Empirical uncertainty and the scientific assessment of ecological harm

If legal uncertainty explains why the threshold is contestable, empirical and evidential uncertainty explain why it is difficult to establish and to code consistently. “Substantial harm” is an indeterminate legal concept, but it is also evidentially demanding. Ecological damage caused by anthropogenic pressures is multidimensional, often cumulative, and not reducible to a single scalar variable. It may involve spatial extent, duration, reversibility, hazardousness, biodiversity effects, ecosystem sensitivity, and interactions between these dimensions. It therefore requires structured methodologies capable of distinguishing levels and intensities of harm while remaining responsive to ecological context and to the limits of the information available in the record ( Morelle-Hungría, 2020).

Scientific research has made significant progress in this direction. Rebelo et al. develop a multi-criteria decision framework for surface waters that integrates chemical, ecological, and functional variables in order to assess “substantial damage” ( Rebelo et al., 2024). Zhang et al. distinguish between quantifiable and non-quantifiable damage in illegal fishing and show how the absence of standardised assessment criteria can undermine the fairness and consistency of judicial compensation ( Zhang et al., 2023). Auffhammer, in a different but relevant domain, shows that climate-damage estimation has evolved towards more sophisticated econometric techniques, while also warning that short-term adaptation effects can distort damage estimates if longer-term dynamics are not adequately modelled ( Auffhammer, 2018). Taken together, this literature supports a limited but important conclusion: ecological harm can be assessed more systematically than traditional legal practice often assumes, but ecological measurement does not by itself establish legal attribution, culpability, evidential sufficiency, normative grading, or the criminal threshold applicable in an individual case.

**Figure 2.**
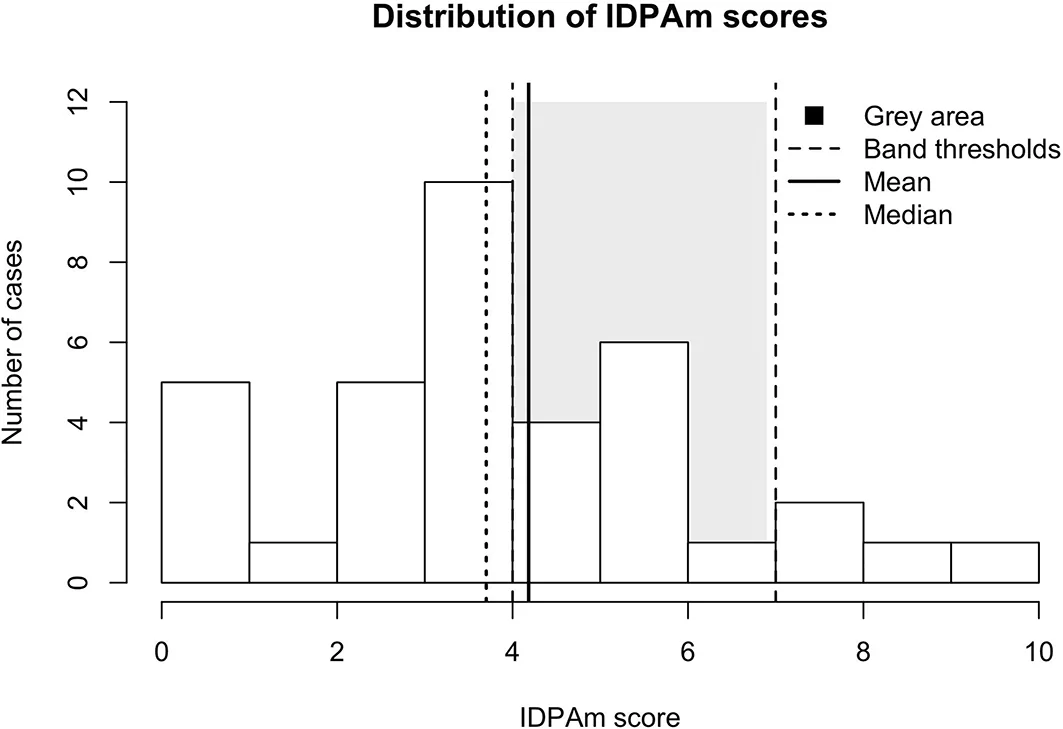
Distribution of IDPAm scores across the analysed sample (n = 36). The shaded area represents the interpretive grey zone proposed by the LEDAT model. Dashed vertical lines indicate the lower boundary of the grey zone and the high-significance threshold; solid and dotted vertical lines indicate the sample mean and median, respectively.

The revised architecture of the Directive makes this point more salient. By requiring Member States to consider baseline condition, duration, extent, reversibility, threshold exceedance, hazardousness, conservation status, and, where relevant, restoration cost, Directive (EU) 2024/1203 recognises that environmental criminal assessment must engage with ecologically granular considerations. At the same time, it requires Member States to strengthen the professional and institutional capacity needed to manage that complexity: national authorities must be provided with sufficient qualified staff and resources; specialised regular training must be available to judges, prosecutors, police, judicial staff, and competent authorities; national coordination mechanisms must promote a common understanding of the relationship between criminal and administrative enforcement; and systems for anonymised statistical data must be established, with a common Union format to follow. These provisions do not prescribe a single assessment model. They do, however, make transparent documentation, specialist capacity, and more comparable treatment of environmental-crime data increasingly relevant to effective implementation. For empirical legal studies, this shifts attention from abstract legal standards to the ways in which institutions identify, document, and assess ecological seriousness.

The Spanish experience illustrates the stakes of this evidential problem. The Prestige litigation provides a useful example. Caballero and Soto-Oñate explain that the Supreme Court’s 2016 judgment did not alter the international liability regime, but used domestic criminal liability to strengthen the practical reach of compensation and the “polluter pays” principle ( Caballero & Soto-Oñate, 2017). The Supreme Court ultimately quashed the previous acquittal, convicted the captain for a reckless environmental offence aggravated by catastrophic damage, and deferred the quantification of compensation to the enforcement phase of the judgment (Supreme Court, 2016, ECLI: ES: TS: 2016: 11). The later transnational litigation culminating in the judgment of the Court of Justice of the European Union in Case C-700/20 showed, in turn, that the legal determination of ecological damage can shape not only liability but also the enforceability of redress across jurisdictions ( Court of Justice of the European Union, 2022, Case C-700/20). These proceedings do not show that quantification solves causation, attribution, evidential sufficiency, or jurisdictional fragmentation. They show why complex ecological cases require reasoning that distinguishes carefully among material harm, legal responsibility, proof, and remediation.

For LEDAT, the methodological consequence is that ecological assessment must be separated from, but connected to, legal and procedural reasoning. The Ecological Component records indicators of material ecological gravity or risk. The Legal Component operates as a distinct normative filter within IDPAm, recording legally relevant aspects of the conduct and offence configuration. IDPAm is therefore not a measure of ecological damage alone, nor is it a measure of criminal liability or a prediction of judicial outcome. Evidential and procedural constraints—including causal uncertainty, weaknesses in expert evidence, attribution difficulties, procedural defects, or the form of case resolution—belong to the separate domain of IDPAe. This separation prevents two opposite analytical errors: treating IDPAm as a pure ecological metric, and treating evidential or procedural failure as if it necessarily demonstrated the absence of materially serious ecological harm.

This distinction also requires an explicit approach to comparability. The evidential relevance of individual ecological variables differs between offence sectors. Concentration or volume may be central to discharges, waste, or emission cases, whereas biodiversity impact, ecosystem sensitivity, or habitat integrity may be more informative in protected-species or protected-area cases. A standardised matrix can facilitate descriptive comparison across heterogeneous decisions only if the coding record identifies the evidential basis for each score and distinguishes four analytically different situations: a score of zero, missing information, insufficient or contested information, and a variable that is not applicable to the factual and ecological profile of the case. Those categories should not be conflated. A documented absence of an ecological feature may warrant a score of zero; lack of sufficiently reliable information does not establish that the feature is absent; and non-applicability requires a sector-sensitive coding rule rather than a mechanically assigned zero.

The model’s credibility therefore depends on a coding structure capable of making uncertainty visible rather than concealing it behind the numerical output. This requires a coding manual with closed anchors, identified evidential sources, explicit rules for missing, insufficient, contested, and non-applicable information, independent pre-reconciliation coding, formal inter-coder agreement procedures, sensitivity analysis for default weights and interpretive-band boundaries, and a strict anti-duplication protocol for triggers. The final dataset must also preserve a case-level audit trail, including the judgment identifier, variable-by-variable scores, evidential justification, trigger activation and its rationale, any application of the anti-duplication rule, and the resulting component and final scores. In empirical applications, these safeguards are not secondary technical details. They are necessary conditions for treating LEDAT as a transparent and auditable methodology rather than as an impressionistic scoring exercise.

### 2.3 A necessary but structurally fragile concept

The reference to “substantial harm” is normatively necessary because environmental criminal law requires a threshold capable of preventing the over-criminalisation of trivial, negligible, or merely technical infringements within a dual sanctioning system. The concept therefore performs an important guarantee function. At the same time, its practical operation remains under-specified for four related reasons.

First, clauses of criminal significance may raise concerns from the perspective of legality because they do not always provide sufficiently determinate criteria for distinguishing criminal offences from administrative infringements ( Fuentes Osorio, 2019). Secondly, the insufficient integration of scientific evidence into the formulation and application of environmental offences may weaken practical enforceability, particularly where ecological complexity is not translated into operationally clear and evidentially grounded criteria ( Morelle-Hungría, 2024). Thirdly, the absence of shared and replicable methodologies for assessing ecological seriousness creates uncertainty in identifying and comparing the relevant dimensions of ecological harm or risk, despite advances in environmental science and damage modelling ( Rebelo et al., 2024; Zhang et al., 2023). Fourthly, judicial interpretation remains decisive not only in the assessment of criminal relevance, but also in defining the practical scope of remediation and compensation, as comparative and transnational litigation illustrates ( Caballero & Soto-Oñate, 2017).

It does not follow that threshold clauses should be abandoned, nor that criminal law should surrender its evaluative structure to a formula. The more careful conclusion is that the problem lies less in the existence of open-textured language as such than in the absence of a shared methodology for identifying, documenting, and examining the considerations through which that language is applied. For empirical legal studies, this is the central point: threshold concepts are not only doctrinal categories. They also organise the classification of cases, and their practical operation can be studied only if the legal, ecological, and evidential factors informing their application are made observable.

Directive (EU) 2024/1203 strengthens the case for more explicit and transparent threshold assessment. It requires clearer national delineation between administrative and criminal enforcement, identifies non-exhaustive factors relevant to the assessment of harm, risk, and quantity, and establishes obligations concerning training, institutional capacity, coordination, and statistical data. Following its transposition deadline of 21 May 2026, the Directive provides an important legal context for examining how environmental criminal seriousness may be documented and compared. It does not, however, prescribe a common scoring system, convert ecological seriousness into a quantified legal test, or displace national legal interpretation and case-specific judicial assessment. LEDAT is accordingly proposed as a methodological framework that may support empirical analysis and structured legal-ecological reasoning within that context, not as an instrument of legal harmonisation or a binding European decision rule.

A restrained formulation is therefore preferable. LEDAT should be understood as a structured interpretative and analytical aid that may improve the traceability and comparability of threshold reasoning, particularly in the “grey area” between administrative illegality and criminal significance. In the empirical pilot, that grey area is an indicative analytical band, not a legally binding category or a statement about the institutional response that a case should receive. Its usefulness depends on whether the model makes the underlying legal and ecological assumptions, together with their evidential basis and remaining uncertainty, more transparent. Its authority does not derive from mathematical determinacy, but from methodological discipline: transparent coding rules; explicit evidential anchors; a documented distinction between zero scores, missing information, insufficient or contested information, and non-applicable variables; independent and reliability-sensitive coding; sensitivity analysis of default weights and interpretive-band boundaries; a strict anti-duplication rule for triggers; and a clear distinction between the Ecological Component, the Legal Component, IDPAm, and IDPAe.

Framed in those terms, LEDAT preserves the central claim of this article: environmental criminal law requires a transparent bridge between legal indeterminacy and ecological complexity, but that bridge must remain contestable, non-binding, and compatible with the constitutional safeguards governing criminal adjudication. In particular, it cannot establish the statutory elements of an offence, prove causation or culpability, determine evidential sufficiency, predict a conviction, or fix a sentence. The following section therefore turns from the threshold problem to the limitations of existing legal, administrative, scientific, and structural approaches when they operate in isolation. This provides the basis for the formal methodological proposal developed later in the article.

## 3. Inadequacy of existing models: normative, epistemological and structural limitations

The limitations of existing approaches to ecological damage in criminal law do not result from a complete absence of analytical tools. They arise primarily from the fragmentation of tools developed for different institutional and disciplinary purposes. Administrative thresholds provide relevant indicators of regulatory non-compliance, but they do not by themselves establish material criminal seriousness. Case-law assessment remains indispensable, particularly in legal systems that treat environmental offences as offences of danger, but it is often affected by recurring difficulties concerning causation, evidential heterogeneity, and judicial reliance on expert evidence. Scientific models show that ecological damage and risk can be described and, in some contexts, quantified with increasing sophistication. However, their outputs are not directly translatable into criminal-law questions concerning statutory thresholds, individual attribution, culpability, evidential sufficiency, or the proportionality of punishment. Finally, approaches drawn from green and Southern criminology show that ecological harm is distributed through unequal political, economic, and institutional arrangements—dimensions that technical assessment models may leave outside their frame of analysis.

The problem, therefore, is not that law, science, or criminology lack relevant concepts. It is that their respective insights are rarely integrated into a common empirical structure capable of making the grounds of threshold assessment visible across cases. Administrative law identifies regulatory breaches; judicial reasoning assesses their legal significance; ecological science examines environmental impact or risk; and critical criminology situates harm within broader structures of power, inequality, and differentiated vulnerability. Each perspective captures an important dimension of environmental wrongdoing, but none by itself provides a reproducible method for documenting and comparing material legal-ecological seriousness in environmental criminal adjudication. This fragmentation affects legal consistency, but it also limits empirical analysis. Where researchers cannot observe how courts and other actors weigh harm, risk, culpability, ecological vulnerability, evidence, and procedural constraints, they cannot systematically analyse the reasoning through which the criminal threshold is approached or contested.

Directive (EU) 2024/1203 strengthens the European framework by identifying relevant criteria for assessing harm, risk, and quantity, and by requiring improvements in resources, specialised training, coordination, national strategies, and statistical infrastructures. However, it does not itself resolve the methodological gap between ecological assessment and criminal adjudication. Against that background, LEDAT is framed as a bridging methodology. Its purpose is not to replace administrative thresholds, judicial interpretation, scientific modelling, or structural critique. Nor is it intended to transform ecological indicators into a legally binding determination of criminal liability. Rather, it seeks to organise selected legally relevant insights from those domains within a transparent, auditable, and contestable coding framework. Its usefulness depends on explicit scoring and evidential rules, sector-sensitive treatment of ecological variables, the documented handling of missing, insufficient, contested, and non-applicable information, reliability-sensitive implementation, sensitivity analysis, and a clear distinction between the Material Environmental Criminal Damage Index (IDPAm) and the Enforceability module (IDPAe).

For that reason, the empirical use of LEDAT in this article must be understood as a pilot exercise in structured case ordering and methodological feasibility, rather than as full statistical validation of a model. The database permits the calculation of IDPAm for 36 first-instance judgments, but it does not support strong inferential claims about judicial outcomes, sector-level patterns, causal relationships, or predictive performance. Nor can convictions and acquittals serve as independent validation criteria, because judicial outcomes may depend on evidential sufficiency, causal and attributional issues, procedural defects, prosecutorial strategy, or conformity-based case resolution, in addition to the material seriousness that IDPAm is designed to structure. The purpose of the empirical application is therefore narrower: to examine whether the framework can be applied coherently to real judgments, whether the resulting scores generate an internally plausible descriptive ordering, and whether the legal, ecological, and evidential assumptions underlying each score can remain traceable when the model is applied to heterogeneous environmental cases.

### 3.1 Administrative thresholds and the limits of regulatory objectivity

Administrative environmental law has long relied on quantitative limits—maximum emission values, discharge thresholds, catch quotas, concentration limits, and protected-species restrictions—to regulate activities capable of causing environmental harm and to support the protection of ecosystem functions. Those thresholds are indispensable within environmental regulation and may provide important evidential anchors in criminal proceedings. They were not, however, designed primarily to assess or grade criminal wrongdoing. Rather, they belong to a broader regulatory framework in which administrative, preventive, civil, restorative, and criminal instruments coexist and interact. As Wade et al. observe, environmental sanctions within such systems are often organised around deterrence, compliance, economic benefit, prior history, and restoration, rather than around a dogmatically elaborated criminal metric of ecological gravity ( Wade et al., 2002). For that reason, an administrative exceedance may be highly relevant to criminal assessment without being conclusive; conversely, materially serious ecological harm or risk may arise in circumstances not adequately represented by a pre-existing regulatory threshold.

For empirical legal analysis, this distinction matters because regulatory thresholds are attractive but potentially incomplete measurement inputs. They are attractive because they are formally defined, frequently quantitative, and comparatively easy to identify in judicial records. They are incomplete because an exceedance, considered alone, may not capture the duration, spatial extent, reversibility, ecological sensitivity, biodiversity effects, hazardousness, cumulative consequences, culpability, or wider ecosystemic significance of the harm. Treating regulatory breach as equivalent to criminal seriousness would collapse a multidimensional assessment into a single administrative indicator. It would also risk reproducing the substantive and evidential limits of the regulatory scheme itself, especially where its parameters do not adequately reflect cumulative, delayed, or ecosystem-level harm.

Directive (EU) 2024/1203 confirms this point at the level of Union law. The recast Directive strengthens the threshold structure of environmental criminal offences and clarifies that, when assessing “substantial damage,” likely damage, or non-negligible quantities, Member States must take into account, where relevant, factors such as the baseline condition of the affected environment; the duration, extent, and reversibility of the damage; the extent to which a regulatory threshold or mandatory parameter has been exceeded; the hazardousness of the activity or substance; the conservation status of the species concerned; and, where feasible, the cost of restoration. Threshold exceedance is therefore an important evidential and contextual factor, but not a self-sufficient measure of material criminal seriousness. The Directive narrows the interpretative field by identifying considerations that should inform the assessment, without equating criminal relevance with regulatory breach alone.

Illegal fishing illustrates this limitation. Morelle-Hungría notes that some forms of IUU fishing may account for a substantial proportion of catches of certain species, thereby affecting not only the target species, but also the food webs and ecological balances in which it is embedded ( Morelle-Hungría, 2017). Yet a quota breach or the taking of protected specimens may not, on its own, capture the full ecological significance of the conduct: relevant considerations may include the conservation status of the affected population, the scale and repetition of the activity, reproductive effects, the sensitivity of the habitat, and the wider ecological relationships affected. Zhang et al. likewise show that the absence of standardised rules for applying guidance documents creates uncertainty in the judicial assessment of ecological damage in illegal-fishing cases ( Zhang et al., 2023), whileValeije Álvarez observes that criminal law has been applied only marginally in this domain ( Valeije Álvarez, 2023). The broader lesson is limited but important: administrative metrics can identify regulated conduct and regulatory non-compliance, but they do not, without further legal, ecological, and evidential analysis, provide a measure of criminal relevance.

For LEDAT, the implication is methodological rather than rhetorical. Administrative thresholds are treated as structured evidential inputs, particularly within the ecological variables relating to concentration or volume, hazardousness, and protected status. They are not treated as proxies for criminal seriousness in themselves. The value of a legal-ecological coding framework lies in making explicit how regulatory exceedance interacts with duration, reversibility, ecosystem sensitivity, biodiversity impact, hazardousness, and legally relevant features of the conduct. In the empirical application, this is why variables such as CONC, SUS, ECO, BIO, and REV are coded separately rather than collapsed into a single indicator of regulatory breach. The coding record must also identify the evidential source of any regulatory threshold, the degree of exceedance where this is reported, and whether the information is sufficient for coding. Where the judgment does not establish a relevant threshold, degree of exceedance, or comparable quantitative information, that absence must not be treated as proof that no ecological feature was present. In this sense, administrative objectivity can inform and discipline criminal assessment, but it cannot replace the legally and evidentially grounded assessment required in the individual case.

### 3.2 Limits of case-law assessment: causation, expert evidence, and the tension between danger and injury

Unlike forms of loss more commonly addressed by private law, ecological damage in criminal law does not ordinarily converge on a clearly identifiable victim or an easily individualisable proprietary interest. It may affect collective and diffuse goods—ecosystems, biodiversity, and the quality of air, soil, and water—whose degradation can develop cumulatively, interact with natural background conditions, and become visible only after a temporal delay. As Silva Sánchez and Montaner Fernández point out, polluting activities frequently generate cumulative or synergistic effects that cannot be reduced to a simple linear chain of causation ( Silva Sánchez & Montaner Fernández, 2012). Bonorino and Leal similarly emphasise that ecological causation is often assessed under conditions of scientific uncertainty, dispersed sources, and difficulties in attributing both environmental effects and responsible conduct with precision ( Bonorino & Leal, 2010). The difficulty is therefore twofold: establishing that relevant ecological harm or risk exists, and establishing whether, and to what extent, a particular act or omission is causally relevant within a dynamic environmental system.

These difficulties are intensified by the partial character of valuation methods that have traditionally informed judicial reasoning. Some approaches privilege the market value of the affected resource; others focus on restoration costs or associated economic losses. Yet such approaches do not fully capture the ecological, functional, and systemic dimensions of environmental degradation. Abbad and Gutiérrez underline that no comprehensive expert methodology satisfactorily translates ecological impacts that may constitute a criminal offence into economic terms, precisely because ecological damage is not reducible to the loss of an individualisable asset ( Abbad & Gutiérrez, 2015). The same authors stress that valuation methods remain partial even where they quantify market value, restoration cost, or derivative losses ( Abbad & Gutiérrez, 2015). Rivera-Olarte reaches a similar conclusion in relation to restoration: the heterogeneity and dynamism of ecological processes resist simplification within conventional valuation frameworks ( Rivera-Olarte, 2017).

In this context, expert evidence becomes indispensable. As Górriz Royo argues, expert reports must not only address pollution and its ecological implications; they must also translate scientific information into legal categories that courts can examine and apply ( Górriz Royo, 2015). The result is a hybrid process in which ecological science, technical expertise, and criminal-law reasoning converge, often without a common methodological framework for documenting how factual findings are related to legally relevant assessments of seriousness. This does not mean that expert disagreement is arbitrary or that judicial reasoning lacks structure. It does mean that, where the methodological premises of expert and legal assessments are not made explicit, comparable cases may be assessed through different combinations of ecological, legal, and evidential considerations.

Spanish case law addresses part of this difficulty by treating environmental offences as offences of danger. Supreme Court Ruling 81/2008 states that the protected legal interest is not merely compliance with administrative regulations, but the environment itself as a collective interest of a supra-individual nature; criminal liability depends on whether the conduct is capable of creating a serious danger to that interest, which in turn requires technical assessment supported by expert evidence (Supreme Court, 2008, ECLI: ES: TS: 2008: 1028). Fuentes Osorio correctly notes that the danger-offence structure permits criminal intervention before irreversible ecological damage materialises, but also that the vagueness of the offence elements makes criminally relevant danger difficult to define with precision ( Fuentes Osorio, 2012). Górriz Royo adds that the gravity of danger must ultimately be related to the ecological disturbance caused or threatened, so criminal assessment remains substantially dependent on technical judgments regarding intensity, probability, geographical reach, duration, and reversibility ( Górriz Royo, 2015).

The distinction between injury and danger should not, however, obscure the separate evidential questions involved. A case based on completed ecological injury may require proof of the existence, extent, duration, and causal attribution of the damage. A case based on danger may require a technically grounded assessment of the seriousness, plausibility, and legally relevant probability of the threatened harm. In both situations, the court must apply the elements of the offence, evaluate the available evidence, and determine whether the burden and standard of proof have been met. A structured coding framework cannot discharge any of those functions. It can only record, in a transparent and comparable manner, the material features that the judgment identifies as relevant to ecological seriousness or risk.

From an eco-criminological perspective, White is right to insist that ecological harm cannot be addressed solely through a reactive punitive lens. Prevention, contextual analysis, situational controls, and structural understanding remain essential, particularly because ecological harm is frequently cumulative and partly irreversible ( White, 2022). hat insight is relevant to LEDAT, but it does not enlarge its legal function. IDPAm is not a pure measure of ecological harm; it is an integrated legal-ecological indicator of material seriousness as structured by the framework. IDPAe concerns the distinct question of procedural and evidential viability. This separation prevents the conflation of substantive material gravity with prosecutorial success or judicial outcome. It also allows future applications to distinguish, where the judicial record permits, between cases involving actual injury, technically substantiated danger, or both. Causal uncertainty, weaknesses in expert evidence, attribution difficulties, conformity dynamics, and procedural barriers should not be converted into lower IDPAm values; they belong, instead, to the enforceability analysis or should be recorded as evidential uncertainty where IDPAe cannot be coded.

The European relevance of this clarification is considerable. Directive (EU) 2024/1203 does not eliminate judicial discretion, but it requires Member States to ensure sufficient qualified staff and adequate financial, technical, and technological resources for authorities involved in the detection, investigation, prosecution, and adjudication of environmental crime. It also requires specialised regular training for judges, prosecutors, police, judicial staff, and competent authorities, as well as strategic and operational coordination between criminal and administrative enforcement bodies. These provisions reinforce the value of explainable and contestable methodologies over unstructured or undocumented assessment. In empirical terms, they also underline why convictions, acquittals, and conformity-based resolutions cannot operate as simple external validations of ecological seriousness. Judicial outcomes are relevant descriptive information, but they remain mediated by evidence, causation, attribution, procedure, legal strategy, and the particular form of case resolution. They must therefore be analysed separately from the material legal-ecological seriousness that IDPAm is designed to structure.

### 3.3 Scientific models: analytical progress and legal limits

Scientific and technical research has made substantial progress in the description, modelling, and quantification of ecological harm. Quesada-Ruiz et al. develop spatial models based on Random Forest techniques to identify geographical patterns of environmental crime in the Canary Islands and relate those patterns to land use and socio-economic variables bles ( Quesada-Ruiz et al., 2023). Cañizares Galarza et al. propose a multi-criteria approach that weights environmental indicators and aggregates them using ordered weighted average operators ( Cañizares Galarza et al., 2021). The Ministry for Ecological Transition has produced guidance for determining the significance of environmental damage under Law 26/2007, using variables including extent, intensity, temporal scale, baseline condition, and risk to human health ( Ministerio para la Transición Ecológica, 2019). These developments show that ecological damage is not analytically intractable: it can be described, modelled, and, within the limits of the available data and the purpose of each model, quantified in meaningful ways.

Analytical progress in environmental science does not, however, automatically translate into legal adequacy in criminal law. Castañón del Valle notes that the assessment of ecological damage continues to be affected by doctrinal, legal, jurisprudential, and scientific shortcomings, and observes that the traditional civil-liability system is “taking on water” when confronted with environmental damage ( Castañón del Valle, 2006, p. 91). Bonorino and Leal similarly argue that traditional liability regimes are structurally ill-equipped for ecological damage because it is diffuse, collective, ongoing, and not easily individualised. Even where legal systems reduce evidential burdens through presumptions or circumstantial reasoning, the underlying complexity of causal attribution remains ( Bonorino & Leal, 2010). The point is not that scientific metrics lack legal relevance. Rather, they generally assess damage, exposure, risk, restoration, or environmental management in terms that do not themselves determine the statutory elements of an offence, causal attribution, individual culpability, evidential sufficiency, or the legally appropriate penal response.

For empirical legal studies, this distinction is important. A scientifically sophisticated model may describe ecological degradation accurately while remaining unsuitable for coding the way criminal courts assess threshold seriousness in particular cases. Conversely, a legally useful coding framework need not claim to be a complete biophysical measure of ecological damage. Its task is different: to organise legally relevant ecological information, together with the legally relevant configuration of the conduct, into variables that can be documented transparently, compared across cases, and contested by the parties and the court. The relevant question is therefore not whether legal coding should replace scientific modelling, but how scientific information can be recorded and structured so that its role in legally mediated assessments of seriousness becomes observable and reproducible.

This is the limited contribution that LEDAT seeks to make. It does not transform environmental criminal adjudication into a scientific measurement exercise, nor does it convert a scientific finding into a legal conclusion. It organises selected legal and ecological considerations through defined variables, explicit evidential anchors, and a transparent integration structure. The Ecological Component records dimensions of ecological gravity or risk; the Legal Component records the normative configuration of the case, including offence classification, culpability-related factors, aggravating circumstances, and recidivism or continuity. The resulting IDPAm is therefore an integrated indicator of material legal-ecological seriousness, not a direct measure of ecological damage, criminal liability, likelihood of conviction, or sentence severity.

The default weighting scheme (Wj = 0.4; We = 0.6) should consequently be understood as a provisional and theory-informed modelling choice, rather than as a statistically discovered parameter. It gives greater weight to the Ecological Component because the framework is intended to structure ecological materiality, while retaining the Legal Component as a normative filter. The underlying variables are ordinal and heterogeneous, and the index should therefore be interpreted as a structured ordinal-comparative approximation, not as a metrically exact representation of ecological criminal seriousness. In the pilot database, plausible alternative weighting schemes produce highly similar ordinal case rankings. This supports the internal robustness of the descriptive ordering within the tested range, but it is only an exploratory sensitivity result. It does not amount to statistical calibration, predictive validation, or external validation of the weighting structure.

The same caution applies to the trigger system. Triggers should operate only where a qualitative feature of the case is not already fully captured by the ordinary variables. This limitation is necessary to prevent double counting between biodiversity impact, ecosystem protection, hazardousness, spatial extent, and comparable factors. The pilot application shows that trigger removal affects the classification of some cases, so triggers are not merely decorative additions to the index. Their substantive effect makes documentation especially important: each activation must be evidentially justified, the reason for satisfying the anti-duplication rule must be recorded, and the cumulative adjustment must remain capped. These safeguards do not make LEDAT deterministic. They make its assumptions visible, auditable, and open to methodological and legal challenge.

Directive (EU) 2024/1203 provides a further reason to formalise these methodological safeguards. The Directive requires Member States to record, publish, and transmit anonymised statistical data on reporting, investigation, prosecution, adjudication, dismissals, convictions, legal-person liability, and penalties, with a common Union format to follow. A structured methodology intended to support empirical environmental-crime analysis should therefore be designed from the outset to facilitate traceability, comparability, and subsequent review. In practical terms, this requires more than a final IDPAm score. The case-level record should preserve the underlying Legal and Ecological Component variables, the calculation of component and final scores, all trigger activations and the associated anti-duplication assessment, the factual and evidential source supporting each assigned value, any coding uncertainty or non-applicability, and the procedural outcome where available.

### 3.4 Structural perspective: ecological justice and global asymmetries

The assessment of ecological damage is not only a technical or doctrinal problem. Green criminology and Southern criminology have shown that ecological harm is also shaped by relations of power, asymmetries in regulation and enforcement, and the unequal distribution of environmental burdens across territories and populations. Walters and Fuentes Loureiro argue that ecological damage often follows patterns of structural inequality connected to the global economy. Their discussion of hazardous-waste transfers from industrialised countries to regions of the Global South illustrates how ecological costs may be externalised to territories with weaker institutional capacities, while the economic benefits remain elsewhere ( Walters & Fuentes Loureiro, 2020). In such contexts, legal visibility is not determined by the material characteristics of harm alone. It is also shaped by geopolitical position, regulatory capacity, institutional resources, and the ability of affected communities and ecosystems to become visible within legal processes.

For empirical legal analysis, this point has direct methodological consequences. Case datasets are not neutral mirrors of the incidence, distribution, or severity of ecological harm. They are shaped by enforcement priorities, inspection capacity, reporting practices, prosecutorial discretion, access to specialist expertise, institutional resources, procedural filters, and the kinds of harm that legal systems are able or willing to recognise. A dataset of judgments therefore records adjudicated and documented cases, rather than the full universe of environmental degradation or environmental offending. This does not make judicial datasets unusable. It does, however, require caution in interpreting what their distributions represent. A low number of cases in a sector, or a low average material-seriousness score, may reflect lower ecological gravity; it may equally reflect under-detection, under-reporting, under-prosecution, evidential barriers, unequal enforcement capacity, or the limited doctrinal recognition of particular forms of harm.

This structural dimension intersects with the distinction between damage affecting identifiable interests and “pure ecological damage.” Rivera-Olarte stresses that ecological damage may directly affect ecosystems or biodiversity even where no immediate injury to identifiable persons can be demonstrated ( Rivera-Olarte, 2017). Cafferatta similarly highlights the difficulty of fitting diffuse, collective, and transgenerational ecological harms into legal categories that privilege identifiable victims and patrimonial loss ( Cafferatta, 2004). As a result, some forms of ecologically serious degradation may remain only partially visible in legal systems, particularly where no direct victim anchors a complaint, where impacts emerge over long periods, or where causal pathways are diffuse and contested.

Noise pollution provides a useful example of that selective visibility. García Ruiz and South show that, although its effects on well-being, wildlife, and ecological balance are documented, noise is still predominantly addressed as an administrative or regulatory issue rather than as a central form of ecological criminal harm ( García Ruiz & South, 2019). The pilot database used in this article reinforces the need for caution when interpreting this field. Noise-related cases form the largest sectoral group in the corpus, but generally occupy the lower range of IDPAm values. This descriptive pattern should not be read as a general finding that noise pollution lacks ecological or criminal relevance, nor as evidence that it is inherently less serious than other environmental harms. It reflects the specific factual, legal, evidential, and procedural configuration of the judgments selected for this pilot. The point is not that every environmentally harmful practice that receives limited criminal attention should be brought within criminal law. Rather, it is that the legal salience of ecological harm is culturally, institutionally, and evidentially mediated. What appears less serious in a judicial dataset may in part reflect the manner in which harm has been detected, framed, documented, charged, and adjudicated rather than its ecological insignificance.

For LEDAT, the implication is one of interpretative caution rather than a proposal to convert structural inequality into an additional numerical variable. The index is designed to structure the material legal-ecological seriousness of cases contained in the available judicial record. It does not measure the prevalence of ecological harm, correct for differential detection or prosecution, rank environmental sectors by their real-world severity, or identify the harms absent from the dataset. Materially similar ecological harms may differ in legal visibility because of enforcement priorities, evidential infrastructures, institutional capacities, procedural routes, conformity-based resolutions, or the diffuse character of the affected good. LEDAT outputs must therefore be interpreted alongside a broader account of ecological justice, institutional recognition, and dataset construction, particularly where the protected good is diffuse, the harm is cumulative, or the relevant environmental burden has become socially normalised.

### 3.5 The need for a bridging methodology

The foregoing discussion suggests that the central problem is not the absence of legal concepts, scientific tools, or critical perspectives considered in isolation. It lies in the limited articulation between them when ecological harm must be assessed within criminal proceedings or studied through judicial decisions. Administrative law provides regulatory thresholds, but not a complete measure of material criminal seriousness. Case law provides doctrinal categories and case-specific reasoning, but often without a common framework for documenting how ecological evidence, legal classification, and assessments of seriousness are connected. Scientific models provide increasingly refined ways of describing environmental damage and risk, but their outputs do not, by themselves, determine criminal relevance, causal attribution, culpability, evidential sufficiency, or penalty. Structural approaches reveal the unequal production, distribution, and legal recognition of ecological harm, yet these dimensions are not always visible in trial-level judicial records. Existing approaches are therefore limited not because they are individually without value, but because they remain institutionally and epistemologically fragmented.

A bridging methodology is needed at that point of fragmentation. For empirical legal studies, such a methodology must do more than restate legal standards or import scientific metrics into legal analysis. It should provide a disciplined way to identify, document, and compare the legally relevant ecological information contained in the available record. It should organise selected ecological dimensions through defined legal-ecological variables; retain a clear distinction between material seriousness and procedural or evidential enforceability; make the assumptions behind scoring, weighting, and classification visible; and preserve judicial discretion by remaining non-binding and open to challenge. A framework of this kind cannot make ecological complexity disappear or produce a legally self-executing answer. It can, however, provide a common structure through which the factual, ecological, legal, and evidential premises of threshold reasoning become more observable and contestable. This is the limited explanatory space within which LEDAT is situated.

LEDAT is not presented as a completed solution, a sentencing grid, or a validated decision instrument. It is a structured methodological proposal whose present empirical support remains pilot in nature. Its contribution lies in making explicit assumptions, variables, evidential bases, trigger decisions, and weighting choices that may otherwise remain implicit in environmental criminal threshold reasoning. The framework does not replace the statutory elements of environmental offences, judicial evaluation of evidence, or the individualised assessment of causation, culpability, proportionality, and sentence. By making the considerations behind a material-seriousness assessment more transparent, LEDAT seeks to support descriptive comparison and reasoned scrutiny without converting judicial assessment into a mechanical exercise.

In the present article, the pilot application concerns 36 first-instance judgments issued by Criminal Courts and Provincial Courts acting as trial courts according to the applicable procedural rules. The empirical exercise supports only cautious claims. It shows that the framework can be applied to heterogeneous judgment material, that it generates a traceable descriptive ordering of IDPAm scores, and that it permits descriptive comparison across the judicial outcomes and sectoral labels represented in the corpus. It does not establish predictive validity, causal effects, sector-level regularities, population-level generalisability, or a validated classification threshold. Nor should the comparison between IDPAm and judicial outcomes be treated as an independent test of the model, since convictions, acquittals, and conformity-based resolutions also reflect evidential sufficiency, causal and attributional issues, procedural conditions, case strategy, and the form of case resolution.

The empirical analysis includes exploratory sensitivity checks of the default legal-ecological weighting structure and of trigger removal. Those checks assess whether plausible alternative modelling assumptions materially alter case ordering or band assignment within this specific pilot corpus; they do not constitute statistical calibration or external validation. Further development should expand the dataset across time, offence sectors, and jurisdictions; report formal inter-coder reliability measures from independent pre-reconciliation coding; test alternative treatments of missing, contested, and non-applicable information; document trigger activation and anti-duplication decisions at case level; distinguish conformity-based from contested convictions; and incorporate IDPAe systematically. The future inclusion of IDPAe is particularly important because it can help examine the difference between material legal-ecological seriousness, procedural and evidential viability, and judicial outcome without collapsing those dimensions into a single score.

## 4. A formal legal-ecological coding framework: The LEDAT model

LEDAT ^®^ (Legal-Ecological Damage Assessment Tool) is a structured coding framework designed to organise the assessment of criminally relevant ecological harm or risk in environmental cases. Its central contribution lies in combining two analytically distinct components—a Legal Component and an Ecological Component—into a composite legal-ecological indicator, the Material Environmental Criminal Damage Index (IDPAm). At the same time, the framework preserves a separate Enforceability module (IDPAe) for procedural and evidential viability.

The distinction between these dimensions is central to the model. The Ecological Component records structured indicators of ecological gravity or risk. The Legal Component records the normative criminal configuration of the case, including offence classification, culpability-related factors, aggravating circumstances, and recidivism or continuity. IDPAm therefore measures neither ecological damage in isolation nor criminal liability. It is an integrated indicator of material legal-ecological seriousness as structured by the model. A higher IDPAm score indicates that the case displays a higher level of such material seriousness within the coding framework; it does not establish that an offence has been proved, that causation or culpability has been demonstrated, that the available evidence is sufficient for conviction, or that a particular penalty should follow. Those issues remain governed by the applicable law, the evidence, and judicial assessment in the individual case. IDPAe addresses a different question: whether a criminal case is procedurally and evidentially viable. It must not be collapsed into IDPAm, because materially serious harm may be difficult to prove or attribute, while a procedurally robust case may concern only moderate material seriousness.

The model is not presented as a fully validated decision instrument, a predictive model, or a sentencing grid. It is a transparent and auditable framework for structuring threshold reasoning and for supporting empirical comparison of judicial decisions. The default weightings (Wj = 0.4; We = 0.6) and the capped trigger system (maximum adjustment of +2) are provisional, theory-informed modelling choices. The greater weighting assigned to the Ecological Component reflects the framework’s ecocentric orientation and its focus on material ecological seriousness; the Legal Component preserves a normative filter so that criminal relevance is not reduced to ecological measurement alone. The purpose of the trigger system is to register qualified forms of ecological escalation that may not be fully reflected in ordinary linear aggregation. It is not intended to multiply gravity mechanically or to duplicate features already captured by the ordinary variables. Because the variables are ordinal and heterogeneous, the resulting scores should be interpreted as structured ordinal-comparative approximations, not as metrically exact measurements.

The methodological credibility of LEDAT depends on several conditions. These include explicit scoring rules and defined evidential anchors; a documented distinction between zero scores, missing information, insufficient or contested information, and non-applicable variables; independent coding before reconciliation and formal assessment of inter-coder agreement; a strict anti-duplication protocol for triggers; case-level traceability of coding decisions; and sensitivity analysis of weighting, trigger, and interpretive-band assumptions. These conditions are particularly pertinent in light of Directive (EU) 2024/1203, which strengthens the European enforcement framework, required transposition by 21 May 2026, and obliges Member States to improve resources, training, coordination, national strategies, and statistical comparability, while leaving national authorities with the practical task of interpreting and applying ecological threshold concepts in individual cases. LEDAT does not supply a binding European standard for that task. It is proposed as a transparent and contestable methodology through which the legal, ecological, and evidential premises of material-seriousness assessment can be documented and examined.

**Figure 1.**
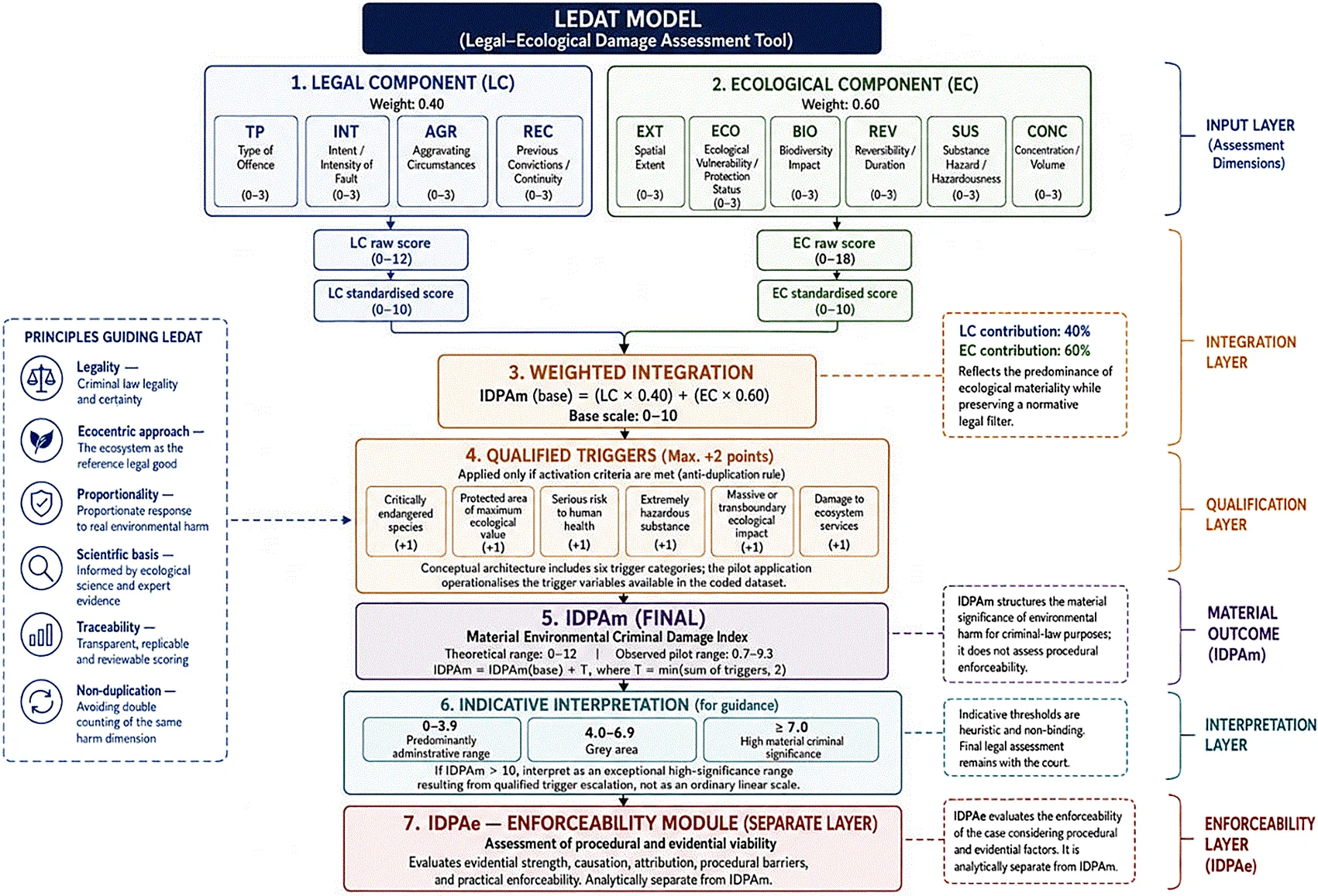
Conceptual architecture of the LEDAT model and integration process for the Material Environmental Criminal Damage Index (IDPAm). The figure distinguishes the Legal Component (LC), the Ecological Component (EC), weighted integration, the capped trigger adjustment, the final IDPAm score, indicative interpretive bands, and the separate Enforceability module (IDPAe). The IDPAm theoretical range is 0–12, while the observed pilot range is 0.7–9.3.

Figure 1 summarises the conceptual architecture of LEDAT and illustrates the interaction between the Legal Component (LC), the Ecological Component (EC), the qualified trigger system, the final IDPAm score, and the separate Enforceability module (IDPAe).

The figure distinguishes the Legal Component (LC), the Ecological Component (EC), weighted integration, the capped trigger adjustment, the final IDPAm score, indicative interpretive bands, and the separate Enforceability module (IDPAe). The theoretical IDPAm range is 0–12: an ordinary weighted base scale ranging from 0 to 10, plus a capped trigger adjustment of up to 2 points for qualified ecological escalation. In the present pilot, observed IDPAm values range from 0.7 to 9.3. The interpretive bands shown in the figure are heuristic and non-binding. They support descriptive comparison but do not establish statutory criminal thresholds, determine liability, classify the required institutional response, or fix sentencing outcomes.

### 4.1 Purpose, scope, and conceptual structure

To address the enduring indeterminacy of clauses such as “substantial damage,” “serious harm,” and “serious risk” in environmental criminal law, LEDAT is proposed as a formalised legal-ecological coding framework. Its purpose is not to replace legal classification, evidential assessment, or judicial discretion, but to structure the analysis of the material seriousness relevant to the distinction between administrative unlawfulness and criminal significance. In that sense, LEDAT is designed to make the factors underlying threshold reasoning more transparent, auditable, and comparable across cases. It does not create a new legal threshold, determine criminal liability, predict judicial outcomes, or prescribe a sentence.

Methodologically, LEDAT should be understood as a formative composite index rather than as a reflective latent-variable scale. The variables included in the model are not assumed to be interchangeable manifestations of a single hidden construct. Instead, they represent distinct legal and ecological dimensions that jointly contribute to the structured assessment of material legal-ecological seriousness. The purpose of the model is therefore not to maximise internal consistency among variables, but to organise heterogeneous yet legally relevant indicators within a transparent and auditable analytical framework. Classical psychometric assumptions associated with reflective scales—such as high inter-item correlation or unidimensionality—are not, by themselves, appropriate criteria for evaluating a framework of this kind. Its methodological assessment should instead focus on the conceptual justification of variables and scores, the transparency of coding decisions, the traceability of evidential sources, inter-coder agreement, sensitivity to plausible modelling choices, and coherence across expanded applications.

Conceptually, LEDAT has four connected elements. The first is the Legal Component (LC), which records the normative criminal configuration of the case within the framework of Articles 325–327 of the Spanish Criminal Code and associated criminal-law doctrine. It includes offence classification, culpability-related factors, aggravating circumstances, and recidivism or continuity. The second is the Ecological Component (EC), which records structured dimensions of ecological gravity or risk, including extent, ecosystem sensitivity, biodiversity impact, reversibility or duration, hazardousness, and concentration or volume where relevant. The third is the structured integration of LC and EC into the Material Environmental Criminal Damage Index (IDPAm), with a capped qualitative trigger adjustment where an exceptional ecological feature is not already fully captured by the ordinary variables. The fourth is the separate Enforceability module (IDPAe), which concerns procedural and evidential viability and is analytically distinct from the material-seriousness assessment.

This architecture requires a precise interpretation of IDPAm. The index is neither a direct biophysical measure of environmental damage nor an ecological measure considered independently of legal context. It is an integrated indicator of material legal-ecological seriousness as structured by the framework. Accordingly, cases involving similar ecological harm or risk may receive different IDPAm scores when their legally relevant features differ. Conversely, a case with a high IDPAm score may still result in an acquittal or other non-conviction outcome because causation, attribution, evidential sufficiency, procedural validity, or the form of case resolution are separate matters. Those considerations belong to judicial assessment and, where sufficient information is available, to the distinct domain of IDPAe. In empirical applications, IDPAm should therefore be interpreted as an ordinal and comparative structuring device, rather than as a metrically exact measure of ecological damage or a proxy for criminal responsibility.

This architecture remains consistent with the revised European framework. Directive (EU) 2024/1203 identifies harm-related factors including baseline condition, duration, extent, reversibility, hazardousness, conservation status, and threshold exceedance, but leaves Member States and courts with the task of applying them in concrete enforcement settings. LEDAT is intended to contribute to the transparent organisation and empirical observation of those considerations; it is not a legally binding method for their application. Its credibility therefore depends less on internal psychometric consistency than on clear coding rules; the distinction between a zero score, missing information, insufficient or contested information, and non-applicability; traceable evidential anchors; formal reliability assessment of independent coding; documented trigger activation and anti-duplication decisions; and the stability of outputs under plausible alternative modelling assumptions.

### 4.2 The legal component

The Legal Component (LC) is a structured coding component of normative criminal seriousness. It is not a measure of ecological harm, a proxy for ecological damage, or an independent measure of criminal liability. It comprises four closed variables, each coded on an ordinal scale from 0 to 3: offence type (TP), intent (INT), aggravating circumstances (AGR), and recidivism or continuity (REC).

The raw legal score ranges from 0 to 12 and is standardised to a 0–10 scale:

LCraw=TP+INT+AGR+REC

$$\mathrm{LC}=\left(\frac{\text{LCraw}}{12}\right)\times 10$$

LC records the normative criminal configuration of a case as represented in the judicial material, including the legal structure of the offence, culpability-related elements, aggravating circumstances, and continuity or repetition of conduct. It does not measure ecological impact as such. This distinction is essential because IDPAm is not a biophysical damage metric: it is an integrated indicator of material legal-ecological seriousness, combining the Legal Component with the Ecological Component and, where justified, the capped trigger adjustment.

The use of consecutive 0–3 scores does not assume that the distance between categories is metrically equal, nor that individual legal variables are interchangeable. The scores are ordinal coding anchors intended to make the legal configuration of cases comparable and transparent. Their aggregation is a formative modelling choice: it provides a structured summary of legally relevant features, rather than a claim that criminal seriousness can be measured with mathematical precision. The standardisation of LC to a 0–10 scale serves only to place it on a common range with the Ecological Component before weighted integration. It does not convert ordinal legal categories into interval-level measurements or establish a legally binding hierarchy of seriousness.

The ordering of the TP variable follows the statutory and doctrinal gradation reflected in Articles 325–327 of the Spanish Criminal Code, as specified in the coding matrix. This ordering is not intended to resolve contested doctrinal questions or to create an autonomous ranking of all environmental offences. It records the legal framework through which the judgment addresses the conduct. In cases involving cumulative, overlapping, or alternative legal provisions, coding should follow the provision that forms the operative basis of the court’s merits assessment. Where the judgment retains more than one legally relevant classification, the coding record should identify the principal provision used for TP, record concurrent or alternative provisions in the case notes, and state the reason for the selected score. Where classification remains legally doubtful or the judgment does not provide a sufficiently clear basis for assigning a criminal category, the relevant uncertainty must be documented rather than obscured by an apparently precise score. The coding manual should apply these rules consistently to convictions, acquittals, and other procedural outcomes.

The AGR variable is likewise a structured summary and not a sentencing calculation. A score of 0–3 records the number or pattern of legally relevant aggravating circumstances identified in the coding protocol; it does not imply that every aggravating factor has identical legal weight, that all factors are cumulative in the same way, or that aggravation alone determines the appropriate penalty. This limitation matters because aggravating features may concern different dimensions of conduct, including deliberate action, commercial motivation, organised activity, concealment, repetition, protected status, or the seriousness of the resulting or threatened harm. Their legal relevance may differ across offence sectors and national sentencing frameworks. Within LEDAT, AGR is included only to capture a limited part of the normative configuration relevant to material criminal seriousness. Individualised sentencing remains governed by the applicable statutory framework, aggravating and mitigating circumstances, participation, culpability, proportionality, reparation, and the judicial assessment of the individual case.

For empirical purposes, LC should be understood as one component of the index rather than as an independent validation criterion. Some of its variables are closely connected to judicial reasoning on liability, particularly offence classification, aggravation, and subjective elements. Consequently, when IDPAm is compared descriptively with convictions or acquittals, that comparison cannot be treated as an external test of predictive validity. The Legal Component partly reconstructs the normative legal configuration of the case, while judicial outcomes also depend on evidential sufficiency, causal attribution, procedural validity, prosecutorial strategy, and the form of case resolution. The presence of conformity-based convictions reinforces this caution. Outcome comparisons are therefore descriptive examinations of the relationship between IDPAm distributions and adjudicated results; they are not validation tests of the model and do not show that a higher IDPAm score causes, predicts, or requires conviction.

The clarification is also important from the perspective of legality and proportionality. Directive (EU) 2024/1203 distinguishes between offence definitions, aggravating circumstances, sanctions, restoration-related measures, and broader enforcement obligations. LEDAT follows that separation. LC records selected legal features for the limited purpose of structured comparative analysis; it neither adds to the statutory elements of an offence nor converts aggravating circumstances into an automated determination of liability or sentence.

### 4.3 The ecological component

The Ecological Component (EC) is the model’s structured coding component for material ecological gravity or risk. It is analytically independent of culpability and procedural enforceability, although its coding necessarily depends on the factual and evidential material available in the judicial record. EC consists of six ordinal variables, each coded from 0 to 3: spatial extent (EXT), ecosystem sensitivity or protection status (ECO), biodiversity impact (BIO), reversibility or duration (REV), substance type or intrinsic hazardousness (SUS), and concentration or volume (CONC).

The ordinary raw ecological score ranges from 0 to 18 and is standardised to a 0–10 scale:

ECraw=EXT+ECO+BIO+REV+SUS+CONC.

EC=(ECraw/18)×10.

This formulation applies where all six variables are applicable and can be coded from sufficiently identified evidence. It should not be read as requiring that every environmental offence display the same ecological profile or generate the same type of evidential information. In particular, concentration or volume may be central to discharges, waste, and emission cases, while biodiversity impact, ecosystem sensitivity, habitat integrity, or duration may be more informative in protected-species, protected-area, extraction, or land-use cases. EC is therefore not a direct biophysical measurement of environmental damage. It is a structured ordinal-comparative coding of selected dimensions of ecological gravity or risk for the purpose of legal-ecological comparison.

This structure aligns with the ecological considerations identified in the revised European framework. Directive (EU) 2024/1203 requires competent authorities, where relevant, to consider the baseline condition of the affected environment, the duration, extent, and reversibility of the damage, the extent to which legal thresholds have been exceeded, the hazardousness of the activity or substance, and the conservation status of affected species when assessing seriousness. These considerations map onto ECO, REV, EXT, CONC, SUS, and BIO. The mapping is neither exhaustive nor legally determinative. It provides a transparent way to organise ecological features that may be relevant to the assessment of material criminal seriousness, while leaving the legal evaluation of the offence, the proof of causation, and the sufficiency of evidence to the competent legal authorities.

The value of LEDAT depends on turning that conceptual correspondence into operational coding rules. Each variable therefore requires closed score anchors, identified admissible evidential sources, and explicit rules for uncertainty and non-comparability. The coding manual is an integral component of the method, not an optional supplement. For every variable, the case-level record should specify the source relied upon, such as a judicial factual finding, expert report, administrative inspection, laboratory result, official map, protected-area classification, species-conservation listing, or other documentary evidence. It should also provide a short justification for the score assigned.

Four analytically distinct coding situations must be separated. First, a score of 0 may be assigned only where the available material supports the absence, minimal level, or legally non-significant manifestation of the relevant ecological feature, as defined in the score anchor. Secondly, missing information means that the judgment or available record contains no information sufficient to assess the variable. Thirdly, insufficient or contested information means that the variable is addressed but the available material does not permit a reliable assignment under the coding rules, whether because evidence is incomplete, contradictory, or expressly disputed. Fourthly, non-applicability means that the variable does not meaningfully correspond to the ecological and factual profile of the case. These situations cannot be treated as interchangeable. In particular, a lack of information on pollutant concentration does not establish that the concentration was within legal limits, and the absence of a concentration measure in a protected-species case does not establish that no relevant biodiversity impact occurred.

In this pilot, the final EC calculation should be made only from variables that are both applicable and supported by sufficiently identified evidence. Where one or more variables are coded as non-applicable, the EC denominator must be adjusted to reflect the number of applicable variables:

$$\text{EC}=((\sum_{i=1}{\text{EC}}_{i})/3k)\times 10.$$

where k is the number of applicable and codable ecological variables. This rule avoids mechanically lowering a case score because a variable is inapplicable to the offence sector. Where information is missing, insufficient, or contested for an otherwise applicable variable, the case should not receive a final EC or IDPAm score unless the coding manual provides a separately documented and justified imputation rule. In the present pilot, the preferred approach is to preserve that uncertainty explicitly rather than to impute a zero value. Any case for which a complete final score cannot be generated under this rule should be reported separately as not fully codable, together with the affected variables and the reason for non-codability.

This approach requires a minimum evidential coverage rule. A final EC score should be calculated only where at least four applicable ecological variables can be coded with sufficient evidential support and where the variables most central to the factual profile of the case are not missing or unresolved. The coding annex should identify the number of applicable variables, the number of variables coded, all missing, contested, or non-applicable entries, and any implications for the interpretation of the final score. This does not eliminate uncertainty, but it prevents uncertainty from being concealed within a mechanically low score and permits readers to distinguish variation in ecological seriousness from variation in the quality or completeness of judicial documentation.

Table 1 maps the relevant criteria in Directive (EU) 2024/1203 onto the LEDAT variables. The table identifies a conceptual correspondence, rather than an exhaustive or one-to-one translation of the Directive into a scoring system.

**Table 1.** Mapping Directive (EU) 2024/1203 criteria onto LEDAT variables.

Directive (EU) 2024/1203 criterion	LEDAT variable	Operational logic
Baseline condition of the affected environment	ECO/REV	Establishes the ecological context against which degradation or recovery must be assessed
Duration of damage or likely damage	REV	Captures temporal persistence and long-term ecological alteration
Extent of damage or likely damage	EXT	Measures spatial reach or territorial scale of the affected area
Reversibility of damage	REV	Distinguishes short-term, long-term, and irreversible ecological effects
Exceedance of regulatory thresholds or mandatory parameters	CONC	Uses legal or technical limits as evidential inputs without treating them as conclusive of criminal seriousness
Hazardousness of the activity or substance	SUS	Captures toxicological, persistence, bioaccumulative, carcinogenic, or equivalent risk properties
Conservation status of affected species	BIO	Reflects biodiversity vulnerability and protected-species relevance
Protected status or ecological sensitivity of affected area	ECO	Captures Natura 2000 areas, National Parks, SPA/SAC, or equivalent protection status
Restoration cost, where feasible	REV/ECO/IDPAe	May inform reversibility, ecological seriousness, and enforceability, but does not replace ecological assessment
Aggravating circumstances under Directive logic	AGR/triggers	Captures legally relevant aggravation while avoiding double counting with ordinary variables

Table 2 summarises the harmonised scoring structure of the LEDAT model. It integrates Legal and Ecological Component variables into a single coding matrix while preserving their analytical distinction. The score anchors are intended as transparent rules for structured assessment. They do not create autonomous legal thresholds, resolve factual disputes, or determine criminal liability. Every assignment should be made with explicit reference to the evidential material available in the case and to the applicable rule for missing, contested, or non-applicable information.

**Table 2.** Harmonised LEDAT variables and scoring matrix.

Component	Variable	Score 0	Score 1	Score 2	Score 3
Legal Component (LC)	TP — Offence classification	Administrative offence or doubtful criminality	Article 325 CC	Article 326 CC	Article 327 CC
Legal Component (LC)	INT — Intent	Minor negligence	Gross negligence	Recklessness/indirect intent	Direct intent
Legal Component (LC)	AGR — Aggravating circumstances	None	One aggravating factor	Two aggravating factors	Three or more/continuing offence
Legal Component (LC)	REC — Recidivism/continuity	No previous conviction or pattern	Isolated previous conviction	Multiple recidivism	Systematic or structural conduct
Ecological Component (EC)	EXT — Spatial extent	<0.5 ha or minimal impact	0.5–1 ha	1–10 ha	>10 ha or widespread impact
Ecological Component (EC)	ECO — Ecosystem sensitivity	Common or urban area	Transformed or agricultural area	Sensitive ecosystem	Protected area: Natura 2000, National Park, SPA/SAC
Ecological Component (EC)	BIO — Biodiversity impact	Common species	Regionally protected species	Vulnerable species	Endangered, CITES-listed or critically endangered species
Ecological Component (EC)	REV — Reversibility/duration	<1 year	1–5 years	>5 years	Irreversible
Ecological Component (EC)	SUS — Substance hazard	Non-toxic	Toxic	Hazardous	Persistent, bioaccumulative or extremely hazardous
Ecological Component (EC)	CONC — Concentration/volume	Within legal limits	Slight excess	High excess	Critical or massive excess

*Note:* A score of 0 is not a default code for absent, insufficient, contested, or non-applicable information. Those situations must be recorded separately in the coding dataset and handled under the EC coverage and calculation rules described above.

Because the pilot database applies these variables to heterogeneous judicial material, Table 2 should be read as a harmonised coding matrix rather than as a claim that every sector generates the same evidential profile. CONC may be central to discharge, waste, or emission cases, whereas BIO, ECO, and REV may be more informative in protected-species, protected-area, extraction, or habitat cases. The standardised EC score permits qualified descriptive comparison across cases only where the case-level record preserves the factual and evidential justification for every assigned value, the distinction between coded and non-coded variables, and the resulting limits on comparability.

### 4.4 Structured integration and default weighting of the model

The weighted integration of the Legal Component (LC) and the Ecological Component (EC) generates the ordinary 0–10 base scale of the model. The trigger system then operates as a capped exceptional adjustment intended to capture qualified forms of ecological escalation that may not be fully represented by ordinary linear aggregation. The trigger mechanism is not a separate measure of liability or sentence severity. It is a limited qualitative adjustment within the assessment of material legal-ecological seriousness.

Once standardised, the two components are combined as follows:

$${\text{IDPAm}}_{\text{base}}=\left(W_{j}\times \mathrm{LC}\right)+\left(W_{e}\times \mathrm{EC}\right)$$

$$W_{j}+W_{e}=1$$

T=min(∑triggers,2)

$$\text{IDPAm}={\text{IDPAm}}_{\text{base}}+T$$

where

$${\text{IDPAm}}_{\text{base}}\;\in \;\left[0,10\right]$$

T∈[0,2]

IDPAm∈[0,12]

In this formulation, LC represents the Legal Component, EC the Ecological Component, $W_{j}$ the legal weighting coefficient, $W_{e}$ the ecological weighting coefficient, and T the capped qualified trigger adjustment. Mathematically, the final IDPAm may range from 0 to 12. Conceptually, however, the model should be understood as an ordinary 0–10 base scale with a capped exceptional increment of up to 2 points for qualified ecological features. The trigger adjustment is available only where the relevant feature has explicit evidential support and is not already fully represented in the ordinary LC or EC scores. Its purpose is to make exceptional qualitative circumstances visible without allowing them to overwhelm the ordinary weighted structure or produce mechanically inflated scores.

Because LEDAT is a formative composite methodology, the weighting structure does not seek to estimate a latent construct, infer covariance among items, or establish psychometric unidimensionality. The weights instead express a transparent modelling assumption about the relative contribution of legal and ecological dimensions to the structured assessment of material legal-ecological seriousness. The proposed weighting scheme must therefore be understood as theory-informed, explicit, and contestable. It is open to empirical refinement, but it is not a statistically discovered parameter or a claim of metric precision.

The default values— $W_{j}=0.4$ and $W_{e}=0.6$ —are proposed as provisional defaults. The greater weight assigned to EC reflects the ecocentric orientation of the framework and its primary concern with the material ecological gravity or risk associated with the case. The inclusion of LC recognises, however, that environmental criminal seriousness cannot be represented through ecological information alone. Offence structure, culpability-related factors, aggravating circumstances, and recidivism or continuity are legally relevant to the normative configuration of the case. The default weighting therefore reflects the model’s dual purpose: to prioritise ecological materiality while retaining a limited normative legal filter.

The weights do not imply that ecological considerations are universally more important than legal considerations in every criminal-law decision. Nor do they assign a fixed proportion of legal responsibility, proof, or sentence severity to either component. They operate only within the methodological construction of IDPAm. The relative 0.4/0.6 allocation is intended to make the model’s ecological orientation visible and open to scrutiny; it does not establish a hierarchy among constitutional guarantees, statutory elements, evidential requirements, or judicial considerations.

The empirical rationale for the default configuration is necessarily exploratory. In the pilot application, the 0.4/0.6 structure is assessed through comparison with plausible alternative weighting scenarios, including balanced weighting ( $W_{j}=0.5;W_{e}=0.5$ ) and a more ecology-prioritised structure ( $W_{j}=0.3;W_{e}=0.7$ ). The purpose of those scenarios is not to identify a statistically optimal set of parameters. It is to examine whether modest and theoretically plausible changes in component weights materially alter the descriptive ordering of cases or their allocation to the proposed interpretive bands. The weighting scheme and the trigger system must be examined separately: alternative weight scenarios test the ordinary integration of LC and EC, whereas the no-trigger scenario tests the contribution of qualified ecological escalation.

In the present pilot, the reported results suggest substantial ordinal stability under the tested alternative legal-ecological weightings, while trigger removal has a more visible effect on some band assignments. These results support a limited conclusion: within the range of assumptions examined, the descriptive ordering of the available cases is not highly dependent on modest variations in the LC/EC weighting structure. They do not validate the default weights, establish an optimal configuration, prove the external robustness of the index, or show that the resulting bands correspond to legally or ecologically validated thresholds. The effect of trigger removal also confirms that trigger activation cannot be treated as an ancillary feature of the framework. It requires case-level documentation of the trigger concerned, the evidential basis for activation, the application of the anti-duplication rule, and the effect of the cap.

The numerical structure of LEDAT should not be interpreted as expressing mathematical precision in the strict scientific sense. Quantification serves an organisational and comparative purpose: it makes selected legal and ecological considerations explicit, records their contribution to a composite assessment, and enables their variation across cases to be examined. Because the underlying variables are ordinal, heterogeneous, and partly theory-informed, the resulting values should be interpreted as structured ordinal-comparative approximations rather than as metrically exact measurements of ecological criminal seriousness. Similarly, the interpretive bands applied to final IDPAm values are heuristic and provisional devices for descriptive comparison. They do not create statutory criminal thresholds, determine liability, predict conviction, prescribe a penalty, or identify the institutional response that a case should receive.

### 4.5 Trigger activation, anti-duplication rules, and interpretive bands

The trigger system operates as a capped qualitative adjustment designed to capture exceptional ecological features that may not be fully represented through the ordinary linear aggregation of the Legal Component (LC) and Ecological Component (EC). Its purpose is not to multiply severity mechanically, compensate for evidential weakness, or create an additional route to criminal liability. Rather, it introduces a controlled, transparent, and limited correction for qualified circumstances in which ordinary linear integration may understate forms of ecological seriousness that are treated as particularly significant within environmental criminal-law reasoning.

Trigger activation is rule-based. Once the relevant factual and evidential circumstances have been coded and the anti-duplication review has been completed, the corresponding trigger value is automatically assigned. This does not mean that triggers are activated automatically merely because a case concerns a protected species, a protected area, a harmful substance, or a regulatory exceedance. Each trigger requires an identified evidential basis and a documented finding that the relevant qualitative feature is not already fully captured by the ordinary LC or EC scores.

The cumulative trigger adjustment is capped according to the following rule:

T=min(∑triggers,2)

In the present pilot, the raw trigger sum is calculated as:

Traw=T1+T2+T3+T4

and the final trigger adjustment is:

T=min(T1+T2+T3+T4,2)

Accordingly, regardless of the number of qualifying trigger conditions identified, the final adjustment cannot exceed 2 points. The cap is intended to preserve proportionality, prevent exceptional qualitative features from overwhelming the ordinary weighted structure of the model, and avoid treating multiple descriptions of the same ecological circumstance as separate cumulative increments.

Table 3 sets out the conceptual trigger categories, their operationalisation in the present pilot, and the applicable anti-duplication safeguards. Four categories were operationalised through the binary variables T1–T4. Two additional categories—massive or transboundary ecological impact and damage to ecosystem services—are retained as conceptual extensions of the model but were not independently operationalised in the present pilot. They therefore do not contribute to the recorded trigger sum or to the final IDPAm values reported in this article.

**Table 3.** Qualified trigger activation protocol and anti-duplication safeguards.

Conceptual trigger category	Pilot variable	Operational status in the present pilot	Activation condition	Additional score	Anti-duplication safeguard
Critically endangered species	T1	Operationalised	Impact affecting a species classified as critically endangered, listed in CITES Appendix I, or subject to an equivalent category of exceptional conservation concern	1	May be activated only where the exceptional conservation significance is not already fully captured by BIO; the case-level record must identify the distinct qualitative basis
Protected area of maximum ecological value	T2	Operationalised	Impact occurring within a National Park, Natura 2000 core area, SPA/SAC, or equivalent area of maximum ecological protection	1	May be activated only where the exceptional ecological significance of the site is not already fully captured by ECO; the case-level record must identify the distinct qualitative basis
Serious risk to human health	T3	Operationalised	Scientifically or technically supported evidence of severe or widespread exposure creating a serious risk to human health	1	Requires an evidential basis independent of concentration or volume alone and must not duplicate a feature already fully recorded elsewhere
Extremely hazardous substance	T4	Operationalised	Presence of persistent, bioaccumulative, carcinogenic, mutagenic, highly toxic, or equivalently hazardous substances generating exceptional ecological risk	1	May be activated only where the exceptional risk profile is not already fully captured by SUS; the case-level record must identify the distinct qualitative basis
Massive or transboundary ecological impact	Not operationalised	Proposed future extension	Ecological effects extending beyond the immediate affected area or producing large-scale or transboundary ecosystemic disruption	1	Would require demonstrable ecological amplification beyond EXT and a separately specified coding protocol
Damage to ecosystem services	Not operationalised	Proposed future extension	Significant disruption of essential ecosystem functions, including water purification, habitat integrity, trophic balance, or equivalent functions	1	Would require a distinct ecosystemic effect not already captured through EXT, ECO, BIO, REV, SUS, or CONC

**Note:** In the present pilot, only T1–T4 enter the trigger calculation. The raw trigger sum is therefore T1+T2+T3+T4 , and the final adjustment is T=min(T1+T2+T3+T42) . A score of 1 is assigned to each qualifying trigger before the cap is applied. The final trigger adjustment cannot exceed 2 points.

The anti-duplication rule is central to the model. A feature cannot be counted once through an ordinary variable and again through a trigger merely because the trigger employs a more emphatic description of the same circumstance. The relevant question is whether the evidence identifies an additional qualitative feature whose significance remains insufficiently represented after the ordinary variable has been coded. Thus, a maximum BIO score based solely on the conservation classification of an affected species does not automatically justify T1. A maximum ECO score based solely on the protected status of an affected area does not automatically justify T2. A high SUS score based solely on the intrinsic hazardousness of a substance does not automatically justify T4. Likewise, T3 requires technically or scientifically supported evidence of serious risk to human health that is independent of concentration or volume alone.

The anti-duplication rule does not prevent an ordinary score and a trigger from coexisting in the same case. Coexistence is justified only where the trigger captures a distinct, documented, and qualitatively exceptional dimension. For example, a substance may receive a high SUS score because of its intrinsic hazardousness and support T4 only where the record additionally establishes an exceptional toxicological, persistent, bioaccumulative, carcinogenic, mutagenic, or equivalent ecological-risk profile not fully exhausted by the ordinary score. Similarly, a protected site may receive a high ECO score and support T2 only where the evidence identifies a specific ecological significance or impact that extends beyond its formal protection status. In every case, the coding record should identify the factual and evidential basis for activation, the ordinary variable reviewed for possible duplication, and the reason why the qualitative trigger was retained.

This methodological logic is consistent with the structure of Directive (EU) 2024/1203, which distinguishes between offence definitions and aggravating circumstances and provides that aggravating circumstances should apply only insofar as they do not already form part of the constituent elements of the offence. LEDAT adopts an analogous methodological safeguard. Triggers are not cumulative severity multipliers, but capped qualitative adjustments intended to identify exceptional features not fully represented in ordinary coding. They do not determine criminal liability, and their use must remain explicit, evidence-based, auditable, and open to legal and methodological challenge.

The proposed interpretive bands—0–3.9 as predominantly administrative, 4–6.9 as a grey area, and ≥ 7 as high material criminal significance—are heuristic, indicative, and non-binding. Their function is to support descriptive comparison among cases; they do not create statutory criminal thresholds, determine whether the elements of an offence are fulfilled, establish criminal liability, predict conviction, prescribe a penalty, or identify the institutional response that a case should receive. In particular, the expression “predominantly administrative” refers only to a lower material-criminal-significance profile generated by the index. It does not mean that a case was, or should have been, resolved through administrative rather than criminal proceedings.

The bands apply to the final IDPAm score, including any capped trigger adjustment. Because ecological harm and material legal-ecological seriousness are continuous rather than naturally divided into discrete classes, cases close to a band boundary require particular interpretative caution. Band allocations should therefore be read as prompts for examination of the underlying coding profile, rather than as self-executing classifications. Sensitivity analysis should test not only the effect of alternative LC/EC weighting structures and trigger removal, but also the effect of plausible alternative band boundaries.

Where qualifying triggers increase IDPAm above 10, the case should be understood as falling within an exceptional high-significance range. That result reflects capped qualitative ecological escalation rather than ordinary linear accumulation. In the present pilot, the observed IDPAm range is 0.7–9.3; no case therefore exceeds the ordinary 0–10 range after application of the trigger adjustment. This descriptive result must be verified if recoding modifies EC values, trigger assignments, or the treatment of incomplete and non-applicable information.

For empirical legal analysis, the trigger system has three implications. First, each activation must be documented at case level, including the activated trigger variable, conceptual category, factual and evidential basis, anti-duplication review, raw trigger sum, and capped final value of T . Secondly, sensitivity checks must assess how rankings and interpretive-band allocations change when triggers are removed or modified. Thirdly, the effects of triggers must be interpreted separately from the effects of alternative LC/EC weighting structures. In the present pilot, removal of triggers has a more visible descriptive effect on band assignment than the tested alternative legal-ecological weighting configurations. This finding does not validate the trigger protocol. It demonstrates that trigger operationalisation is substantively consequential and that its documentation, reliability, and performance require fuller testing in future applications.

### 4.6 IDPAm and IDPAe: substance and enforceability

A central feature of LEDAT is the distinction between the Material Environmental Criminal Damage Index (IDPAm) and the Enforceability module (IDPAe). The distinction separates two analytically connected but non-equivalent questions. IDPAm concerns the material legal-ecological seriousness of harm or risk as structured through the combined Legal and Ecological Components and, where justified, the capped trigger adjustment. IDPAe concerns the procedural and evidential viability of sustaining a criminal case. It addresses whether the available legal and factual record permits the material seriousness identified through IDPAm to be investigated, charged, proved, attributed, and adjudicated in accordance with the applicable criminal-law requirements.

IDPAe may include factors such as limitation periods; procedural validity and admissibility of key evidence; the robustness, consistency, and availability of expert evidence; causal uncertainty; objective attribution; individual or organisational attribution; chain-of-custody reliability; and the procedural form of case resolution, including conformity-based resolutions. These factors do not necessarily reduce the ecological or material seriousness of the underlying conduct. They affect the capacity of legal institutions to establish and act upon that seriousness through criminal proceedings.

This separation prevents a recurrent analytical error: equating acquittal, dismissal, evidential failure, or another procedural outcome with the absence of material criminal seriousness. A case may involve ecologically grave harm or risk but remain difficult to prove, causally attribute, or prosecute. Conversely, a procedurally robust case may concern only moderate material seriousness. IDPAm and IDPAe should therefore not be collapsed into a single indicator, summed to create an overall score, or treated as opposite poles of the same continuum. They answer different questions and may diverge in either direction.

The distinction is also essential to the interpretation of judicial outcomes. Convictions, acquittals, and conformity-based resolutions are observable, but they are not clean external measures of material ecological seriousness. A conviction may reflect strong and admissible evidence, a readily provable causal link, a legally straightforward offence configuration, prosecutorial strategy, or negotiated conformity. An acquittal may reflect evidential insufficiency, uncertainty concerning causation or attribution, procedural defects, exclusion of evidence, or another feature of procedural viability. Judicial outcome may therefore be descriptively compared with IDPAm, but it cannot validate the index, establish its predictive performance, or demonstrate that higher material seriousness causes, predicts, or requires conviction.

The distinction has a practical implication for the possible use of LEDAT in legal reasoning. IDPAm may help prosecutors, experts, courts, and defence counsel identify and discuss the legal-ecological features that bear on material seriousness. It cannot establish the statutory elements of an offence, prove causation, demonstrate culpability, discharge the burden of proof, or determine the legal result of a case. IDPAe, in turn, may provide a separate framework for identifying the procedural and evidential issues that condition enforceability. Neither module substitutes for judicial assessment; both must remain transparent, source-based, and open to contradiction by the parties.

The minimum operationalisation of IDPAe should include structural obstacles, procedural validity of key evidence, expert robustness, causal link and objective attribution, perpetration or organisational attribution, chain-of-custody reliability, and the procedural form of case resolution. However, the present empirical application is limited to IDPAm. IDPAe is not systematically applied in the pilot because publicly available judgments do not provide sufficiently uniform, detailed, or comparable access to the complete evidential and procedural record. In particular, published decisions do not always disclose the full content of expert disagreement, investigative limitations, potential nullity issues, chain-of-custody questions, informal prosecutorial decisions, or the dynamics underlying conformity-based resolutions.

This limitation must be kept distinct from the conceptual value of the separation. The present pilot can describe whether IDPAm values differ across observed judicial outcomes, but it cannot determine why a particular case resulted in conviction or acquittal, and it cannot empirically attribute any divergence between material seriousness and outcome to specific IDPAe factors. Future research should operationalise IDPAe through a separate, transparent, and independently coded set of procedural and evidential variables, preferably using fuller case files or other sources that provide systematic access to the relevant information. That development is necessary if LEDAT is to examine, rather than merely theorise, the relationship between material legal-ecological seriousness, enforceability, and judicial outcome.

### 4.7 Coding protocol, implementation formats, and methodological transparency

EDAT may be implemented through different formats, including a structured expert matrix, a spreadsheet-based tool, or an open computational module. The choice of format is secondary to the conditions that make the framework methodologically transparent and legally accountable. Whatever format is used, the model must remain explainable, traceable, and contestable. It cannot operate as a black box, and its final numerical output cannot be separated from the factual, evidential, legal, and methodological assumptions on which it depends.

These requirements are not merely matters of good research practice. They are consistent with Directive (EU) 2024/1203’s broader emphasis on resources, specialised training, coordination, risk-based national strategies, and statistical comparability. Any structured methodology used to inform expert, prosecutorial, judicial, or defence reasoning must remain open to contradiction by the parties and to judicial scrutiny. In empirical legal research, the same principle requires reproducibility at two distinct levels. Computational reproducibility requires that other researchers can reproduce the reported calculations, descriptive statistics, figures, and sensitivity analyses from the published coding dataset and scripts. Coding reproducibility requires that they can understand, review, and, where appropriate, independently reassess the factual and evidential basis on which each individual variable and trigger was assigned.

A fully transparent implementation should therefore preserve an auditable coding record. At a minimum, that record should identify the coder responsible for each entry; retain coder-specific values before reconciliation; preserve version history and all subsequent modifications; identify the factual and evidential source supporting each score; provide a short justification for each assignment; record the handling of conflicting evidence; and state explicitly whether a variable was scored as zero, missing, insufficient or contested, or non-applicable. The coding manual must contain more than score anchors. It must also specify admissible evidential sources, rules for resolving conflicts between documentary and expert evidence, the treatment of incomplete information, the procedure for alternative or disputed legal classifications, and the anti-duplication protocol governing trigger activation.

For trigger variables, the audit trail must additionally identify the conceptual trigger category, the relevant pilot field where applicable, the factual basis for activation, the source supporting that finding, the ordinary variable reviewed for possible duplication, the reason why the trigger captures a distinct qualitative feature, the raw trigger sum, and the capped final value of T . These requirements are particularly important because a trigger may affect interpretive-band assignment. A final trigger value without a documented evidential and anti-duplication rationale is computationally reproducible but not fully reproducible as a coding decision.

In empirical applications, the dataset should preserve the information required to reproduce and evaluate the final score. This includes the individual LC variables; the individual EC variables; the status of each ecological variable as scored, missing, insufficient or contested, or non-applicable; the coder-specific and reconciled values; each trigger activation; the raw trigger sum; the capped trigger value; the calculated LC, EC, and IDPAm base score; final IDPAm; interpretive band; court; year; sector; judicial outcome; and procedural disposition where relevant. It should also preserve a concise case-level coding note, cross-referenced to the relevant passage of the judgment or other admissible evidential source. This is particularly important where the framework is applied to heterogeneous judicial material, because the final numerical value is meaningful only if its underlying coding trail is available for audit, replication, and challenge.

For the pilot application reported in this article, the derived case-level dataset, methodological appendix, and replication scripts are made available as part of the empirical contribution. These materials permit computational replication of the reported descriptive statistics, figures, consistency checks, and sensitivity analyses from the reconciled dataset. They also disclose the variables used in the final calculation, including LC/EC inputs, T1–T4 trigger fields, the capped trigger value, final IDPAm, interpretive band, judicial outcome, and outcome type. However, the present pilot materials do not yet preserve complete coder-specific pre-reconciliation records, formal inter-coder reliability coefficients, or a full case-level evidential justification for every variable and trigger assignment. The pilot therefore provides partial coding reproducibility rather than full independent replication of all original coding judgments.

This limitation qualifies, but does not negate, the contribution of the pilot. The present materials allow readers to inspect the structure of the model, reproduce the numerical calculations, verify the reported aggregate results, and assess the explicit coding anchors contained in the methodological appendix. They do not yet allow a new research team to reproduce every original coding decision from a fully preserved source-by-variable audit trail. Future applications should address this limitation by publishing a case-level coding annex with source references, uncertainty codes, trigger rationales, coder-specific pre-reconciliation values, reconciliation notes, and formal reliability statistics. LEDAT’s claim to transparency is strongest when computational reproducibility and coding reproducibility are both available and clearly distinguished.

### 4.8 Inter-coder reliability and pilot application

Because LEDAT is proposed as a replicable coding methodology, its application must be treated as reliability-sensitive. In the present exploratory study, the 36 cases were independently coded by two trained junior researchers using a shared coding manual and an independent parallel coding protocol. Each coder applied the legal variables, ecological variables, and trigger fields separately and without access to the other coder’s classifications before the reconciliation stage. Divergences were subsequently identified, reviewed against the coding manual and the evidential material available in the judgments, and resolved through consensus. The principal researcher supervised protocol design, discrepancy review, and final harmonisation of the reconciled dataset.

This procedure was intended to reduce the influence of individual interpretation and to assess whether the framework could be applied coherently to heterogeneous judicial material. It should not, however, be described as a fully blind design. Judicial decisions ordinarily disclose factual findings, legal classification, evidential reasoning, procedural posture, and outcome. The relevant methodological safeguard lies not in blinding coders to information inherently contained in the judgment, but in independent initial coding, the use of defined coding anchors, documented reconciliation, and the preservation of coder-specific pre-reconciliation records in future applications.

The present pilot does not report formal inter-coder reliability coefficients. Accordingly, any reported coding convergence must be understood as descriptive and provisional, not as a formal estimate of agreement. The reconciled dataset currently permits reproduction of the final scores and reported analyses, but it does not preserve a complete set of coder-specific pre-reconciliation values from which agreement statistics can be calculated retrospectively. The pilot therefore does not establish that independent coders would generate the same variable scores, component scores, trigger activations, or interpretive-band assignments across larger samples, different offence sectors, or other jurisdictions.

Future applications should calculate and report formal agreement measures before reconciliation. For ordinal variables such as TP, INT, AGR, REC, EXT, ECO, BIO, REV, SUS, CONC, and the interpretive band, weighted Cohen’s kappa should normally be reported where there are two coders and complete paired observations. Krippendorff’s alpha with an ordinal distance function may be preferable where there are more than two coders, missing values, or a coding design requiring a more flexible measure of agreement. For the standardised component and composite outputs—LC, EC, IDPAm base score, and final IDPAm—future studies should report an intraclass correlation coefficient based on absolute agreement, together with the relevant confidence intervals and the precise model specification. Agreement should also be reported separately for the binary trigger variables T1–T4, since trigger activation may have a substantive effect on the final band assignment.

Formal reliability assessment should not be understood as a purely technical addition. It is necessary to determine whether the coding anchors, evidential rules, sector-sensitive procedures, treatment of incomplete information, and anti-duplication safeguards can be applied consistently by independent coders. Where agreement is low for a variable, the appropriate response is not simply to retain the reconciled score. The coding manual, evidential anchors, score definitions, and training procedure should be reviewed and refined before the framework is applied more broadly.

The absence of formally reported reliability coefficients qualifies the empirical claims advanced in this article. The present application should therefore be interpreted as a feasibility-oriented coding exercise, rather than as a full reliability validation of LEDAT. It shows that the framework can be applied to real judicial material, that the reconciled data can generate a structured descriptive ordering of cases, and that the implications of selected modelling assumptions can be examined through sensitivity analysis. It does not establish coder-independent reproducibility, statistical calibration of the model, predictive validity, causal effects, or external validation against an independent legal or ecological criterion.

The same caution applies to the wider interpretation of the pilot. The study is based on 36 first-instance judgments issued by Criminal Courts and Provincial Courts acting as trial courts under the applicable procedural rules. It provides an initial examination of operational feasibility, conceptual coherence, and descriptive comparability, but it does not support population-level inference about Spanish environmental criminal enforcement. Nor does the pilot systematically apply IDPAe, which means that it cannot explain the relationship between material legal-ecological seriousness, evidential or procedural viability, and judicial outcome. This limitation is especially important because the corpus includes conformity-based convictions, and those outcomes cannot be treated as methodologically equivalent to fully contested convictions. The empirical contribution of the article therefore lies in structured case ordering, methodological transparency, and the identification of questions for more rigorous future validation—not in prediction, causal inference, or generalisation.

### 4.9 Structural safeguards and legal nature of the model

The use of structured matrices and integration formulas in criminal-law analysis requires a clear account of their legal nature, procedural safeguards, and constitutional limits. LEDAT should therefore be characterised as a structured expert-support framework, an argument-structuring methodology, and an empirical legal-research instrument. It is neither a sentencing grid nor an automated decision-making system. It does not create new offences, redefine the statutory content of Articles 325–327 of the Spanish Criminal Code, replace the burden or standard of proof, or displace the court’s freedom to assess the evidence and apply the law in the individual case.

Rather, LEDAT organises selected factors that may be relevant to the assessment of material legal-ecological seriousness in environmental criminal cases. These include the seriousness of ecological harm or risk, the legal configuration of the offence, culpability-related factors, aggravating circumstances, recidivism or continuity, the protected status or ecological sensitivity of affected ecosystems and species, and the factual characteristics of the ecological impact. The framework does not determine whether those factors satisfy the statutory elements of an offence. Nor does it prove causation, establish attribution, resolve factual disputes, determine evidential sufficiency, predict conviction, or prescribe a sentence. These matters remain governed by the applicable law, the evidence, the adversarial process, and judicial assessment.

For empirical legal studies, this legal characterisation is methodologically important. If LEDAT were treated as a decision rule, its scores could appear to dictate legal outcomes. If it were treated as a purely ecological metric, its Legal Component and normative assumptions would be obscured. If it were treated as a predictive model, the pilot would be assessed against standards it does not claim to satisfy. The model is instead best understood as a framework for coding, documenting, and comparing material legal-ecological seriousness across judicial decisions. Its outputs are interpretive and comparative, not dispositive. They should prompt examination of the underlying variables, evidential sources, uncertainty, and points of disagreement; they should never be treated as a substitute for reasoned legal judgment.

On that basis, LEDAT is designed to operate in a manner consistent with the principles of legality, minimum criminal intervention, culpability, proportionality, and judicial autonomy. It does not add offence elements or create autonomous legal thresholds. It seeks to make more explicit the distinction between regulatory unlawfulness and the material seriousness that may warrant criminal-law analysis, without treating that distinction as mechanically decidable. It does not quantify moral blame in the abstract, but records selected legally relevant aspects of intent, aggravation, and continuity. It does not claim mathematical certainty, but provides an ordinal-comparative structure for examining heterogeneous legal and ecological considerations. These safeguards remain meaningful only if the framework is non-binding, transparent, evidence-based, open to contradiction by the parties, and subject to full judicial scrutiny ( Fuentes Osorio, 2019; Wade et al., 2002; White, 2022; Zhang et al., 2023; Rebelo et al., 2024; Auffhammer, 2018).

The model’s safeguards are both substantive and procedural. Substantively, LC and EC remain analytically distinguishable, IDPAm remains separate from IDPAe, and trigger activation is limited by explicit evidential requirements, an anti-duplication review, and a cap of 2 points. Procedurally, each score must be linked to an identifiable factual or evidential basis; missing, insufficient, contested, and non-applicable information must not be treated as interchangeable; and the coding trail must allow the parties, courts, and independent researchers to identify the assumptions underlying the final score. In a legal setting, any use of LEDAT must preserve adversarial challenge: parties must be able to examine the coding record, contest individual variable assignments, challenge trigger activation, dispute the factual sources relied upon, and argue against the legal relevance of the resulting profile.

LEDAT is therefore most persuasive when described as a structured methodology that may improve the traceability, transparency, and comparability of reasoning about environmental criminal thresholds, particularly in the grey area between administrative unlawfulness and criminal significance. It becomes less persuasive if it is presented as though the present pilot had already established full validation, a universal scoring system, or a scientifically exact measure of ecological criminal seriousness. Its next stage should consist not in stronger claims, but in stricter empirical testing: larger and more diverse datasets; formal inter-coder reliability reporting from independent pre-reconciliation coding; fuller case-level documentation of evidential sources and coding decisions; explicit distinctions between zero values, missing information, contested or insufficient evidence, and non-applicability; documented trigger activation and anti-duplication assessments; sensitivity analysis of weighting, trigger, and band-boundary assumptions; separate treatment of conformity-based and contested convictions; and systematic operationalisation of IDPAe.

LEDAT does not assume that ecological criminal seriousness can be exhaustively reduced to numerical representation. It proceeds from the narrower premise that structured and transparent approximation may make relevant legal-ecological considerations more visible, comparable, and open to challenge under conditions of ecological and evidential complexity. The empirical pilot should therefore be read as an initial feasibility and coherence exercise. It shows that the framework can be applied to a heterogeneous set of 36 first-instance judgments and that the reconciled coding dataset can produce a structured descriptive ordering and computationally reproducible aggregate analyses. It does not establish coder-independent reproducibility of every underlying coding decision, predictive validity, causal explanation, statistically calibrated thresholds, or definitive external validation.

## 5. Pilot empirical application to Spanish environmental crime judgments

This section reports a pilot empirical application of LEDAT to Spanish first-instance environmental crime judgments. Its purpose is limited and methodological: to examine whether the framework can be operationalised in heterogeneous judicial material, whether the reconciled coding dataset can generate a structured descriptive ordering of cases according to the Material Environmental Criminal Damage Index (IDPAm), and whether the implications of selected modelling assumptions can be examined transparently. The empirical exercise is exploratory rather than definitive. It is not designed to estimate the prevalence of environmental crime, describe the full population of Spanish environmental criminal litigation, predict judicial outcomes, establish causal relationships, or validate LEDAT as a decision instrument.

The search strategy covers the period from 1 January 2020 to 31 December 2025 and yields a final valid sample of 36 judgments issued by Criminal Courts and Provincial Courts acting as trial courts under the applicable procedural rules. The corpus is a filtered jurisprudential sample rather than a probabilistic sample or a complete census of Spanish environmental criminal litigation. Cases were independently coded by two trained junior researchers using a shared coding manual and an independent parallel coding protocol, under which each coder worked separately and without access to the other coder’s classifications before reconciliation. Divergences were subsequently reviewed and resolved by consensus according to the coding protocol and the evidential material available in the judgments. Because formal inter-coder reliability coefficients have not yet been calculated from coder-specific pre-reconciliation records, the coding convergence reported in this pilot remains descriptive and provisional. The reconciled dataset permits computational reproduction of the reported calculations and aggregate analyses, but it does not yet provide full independent replication of every original coding decision.

The pilot applies the Legal Component (LC), the Ecological Component (EC), and the capped trigger system in order to calculate IDPAm. The Enforceability module (IDPAe) is not systematically applied at this stage because publicly available judgments do not provide sufficiently uniform, detailed, and comparable access to the full evidential and procedural record. Accordingly, IDPAm must be interpreted as an integrated indicator of material legal-ecological seriousness, not as a measure of criminal liability, evidential sufficiency, procedural viability, probability of conviction, or sentence severity.

The descriptive findings indicate that most cases fall within the predominantly administrative or intermediate grey-area bands of the index, while four cases reach the high material criminal significance band. These bands remain heuristic and non-binding categories for descriptive comparison; they do not establish statutory criminal thresholds or determine the appropriate legal outcome of a case. IDPAm values are descriptively higher among convictions than among acquittals in the pilot corpus, but the distributions overlap substantially. This pattern cannot be treated as an independent validation of the index because judicial outcome is influenced by causation, attribution, evidential sufficiency, procedural conditions, legal strategy, and the form of case resolution, in addition to material seriousness. The distinction between contested and conformity-based convictions is therefore reported separately wherever the available information permits. No predictive, causal, inferential, or external-validation claim is advanced.

### 5.1 Sample selection and search strategy

The empirical basis for the pilot study was drawn from the Tirant Prime jurisprudential database, accessed through the institutional portal of the Universitat Jaume I. The search covered the period from 1 January 2020 to 31 December 2025. It was designed to identify criminal proceedings potentially relevant to environmental offences under Article 325 of the Spanish Criminal Code and used the Boolean search string: “artículo 325” OR “art. 325” OR “delito contra el medio ambiente”. Jurisdictional filters were limited to criminal courts, including High Courts of Justice, Provincial Courts, and Criminal Courts with competence to adjudicate first-instance environmental criminal proceedings.

The search strategy was purposive and jurisprudential rather than probabilistic. Its purpose was not to construct a statistically representative sample of all environmental harm, all environmental offending, or all Spanish environmental criminal litigation. Nor were cases selected on the basis of judicial outcome, ecological seriousness, sector, sentence, or anticipated IDPAm score. The objective was to identify a bounded corpus of first-instance judicial decisions in which environmental criminal relevance was sufficiently central to permit systematic application of the legal-ecological coding framework.

The initial search yielded 1,145 documents. An internal filter for first-instance decisions reduced the working set to 157 judgments. The remaining decisions underwent qualitative screening against predefined inclusion and exclusion criteria. To be retained, a judgment had to: concern a criminal proceeding; be issued at trial level in the procedural sense; contain a substantively relevant environmental-crime question connected to Article 325 or the associated environmental criminal-law framework; and provide sufficient legal, factual, and ecological information to permit structured coding from the publicly available decision. Cases were excluded where the reference to Article 325 was merely tangential; where the provision had been formally pleaded but was legally irrelevant to the final judicial reasoning; where the environmental issue was subsumed within an unrelated offence without a sufficiently developed environmental assessment; or where the available text did not permit meaningful application of the coding protocol.

On that basis, 121 judgments were excluded and 36 decisions were retained for systematic coding. The final number of 36 cases was therefore not fixed in advance as a target sample size. It emerged from the application of the search strategy, the procedural filter, and the substantive eligibility criteria to the material available in the database during the defined period. The corpus should consequently be understood as a filtered sample of codable first-instance environmental criminal judgments, rather than as a sample selected to represent a predetermined distribution of offence types, geographical areas, courts, judicial outcomes, or levels of ecological seriousness.

The final corpus consists of first-instance judgments in the procedural sense: that is, judgments issued by the court competent to adjudicate the case at trial level, regardless of whether that body was a Criminal Court or a Provincial Court. In the final coded sample, 26 judgments were issued by Provincial Courts and 10 by Criminal Courts. No High Court of Justice decision was retained in the final valid corpus. The annual distribution was uneven: 12 judgments correspond to 2020, 10 to 2021, 4 to 2022, 4 to 2023, 4 to 2024, and 2 to 2025. The sectoral composition was likewise heterogeneous, including noise pollution, discharges, waste, emissions, extraction-related cases, atmospheric pollution, protected-area damage, natural-resource cases, and urban-planning matters. Given the limited number of observations in several sectors, subsequent sectoral comparisons are descriptive only and do not support sector-level inference.

The final corpus should therefore be interpreted as a filtered jurisprudential dataset rather than as a complete census of Spanish environmental criminal litigation between 2020 and 2025. The search protocol was designed to maximise recall within the selected database and legal search terms, but it remains dependent on database indexing practices, search terminology, procedural classification, the availability of written judgments, and the limits of the selected source. It may over-include documents in which Article 325 appears incidentally and under-include relevant environmental cases not indexed under the chosen terms, adjudicated under other provisions, or not available in the database in searchable form.

The sample is appropriate for the limited purpose of this pilot: examining whether LEDAT can be applied to heterogeneous judicial material, whether the coding structure can produce a documented descriptive ordering of cases, and whether selected modelling assumptions can be examined through sensitivity analysis. It is not appropriate for estimating the population-level prevalence, distribution, detection, prosecution, conviction, or severity of environmental crime in Spain. Nor does the composition of the corpus permit conclusions about the comparative seriousness of different environmental sectors, court types, or judicial outcomes beyond the cases included in the dataset.

### 5.2 Coding protocol and calculation of the index

Each of the 36 judgments was coded using the LEDAT framework. The coding process was conducted independently by two trained junior researchers on the basis of a shared coding manual defining the legal and ecological variables, score anchors, evidential criteria, and trigger activation rules. The principal researcher designed the protocol, supervised the calibration process, reviewed discrepancies following independent coding, and conducted final harmonisation of the reconciled dataset.

To reduce the influence of individual interpretation, all cases were coded independently before the reconciliation phase. The procedure should therefore be understood as independent parallel coding rather than as a fully blind design in the strict experimental sense. Judicial decisions normally disclose procedural outcome, factual reasoning, legal classification, and evidential assessment; coders could not therefore be blinded to information contained in the judgments themselves. The principal safeguard was the separate initial application of a shared coding protocol, followed by a structured review of divergent assignments.

Discrepancies in variable assignment were subsequently reviewed jointly and resolved through consensus-based discussion grounded in the predefined coding criteria and the documentary and evidential material available in the judgments (see Table 4). The reconciliation stage was not intended to replace independent coding or to demonstrate formal agreement. Its purpose was to produce a single transparent pilot dataset while identifying coding dimensions requiring greater clarification or refinement in future applications.

**Table 4.** Coding protocol used in the pilot application of LEDAT.

Phase	Researcher(s) involved	Function
Protocol design and calibration	Principal researcher	Preparation of the coding manual, variable definitions, score anchors, evidential criteria, and calculation rules
Independent parallel coding	Researcher A	Independent application of the LEDAT coding matrix to the 36 judgments
Independent parallel coding	Researcher B	Independent application of the LEDAT coding matrix to the 36 judgments
Discrepancy review	Principal researcher and coders	Identification and comparative review of divergent variable assignments and trigger activations
Reconciliation phase	Joint review	Consensus-based final assignment according to the coding manual and available evidential material
Final harmonisation	Principal researcher	Review of internal consistency, calculation checks, and preparation of the reconciled dataset

The pilot does not report formal inter-coder reliability coefficients. Table 5 therefore presents qualitative observations from the reconciliation process, not quantitative estimates of inter-coder agreement. These observations should not be interpreted as Cohen’s kappa, Krippendorff’s alpha, intraclass correlation coefficients, or any other formal reliability measure. The coder-specific pre-reconciliation values have not been preserved in the currently available dataset; consequently, formal agreement statistics cannot be calculated retrospectively from the reconciled pilot file.

**Table 5.** Qualitative observations from the pilot reconciliation process.

Coding Dimension	Qualitative observation during reconciliation	Main area requiring further clarification
Legal Component (LC)	Divergences were limited in several cases, but required review of legal classification and aggravating circumstances	Rules for overlapping, alternative, or legally disputed classifications; distinction between aggravation and continuity
Ecological Component (EC)	Divergences were more frequent where ecological information was incomplete, qualitative, or unevenly documented	Evidential anchors for reversibility, duration, ecosystem sensitivity, and sector-specific relevance of variables
Trigger activation	Trigger assignments required case-specific review, particularly where ordinary scores and qualified circumstances overlapped	Evidential basis, T1–T4 category label, anti-duplication assessment, and justification for coexistence with ordinary scores
Overall IDPAm band	Most discussion concerned cases near adjacent interpretive-band boundaries	Sensitivity to weighting assumptions, trigger treatment, and alternative band boundaries interpretive bands

The calculation of IDPAm follows the structure presented in Section 4. The Legal Component (LC) records normative criminal configuration through offence type (TP), intent (INT), aggravating circumstances (AGR), and recidivism or continuity (REC). The Ecological Component (EC) records material ecological gravity or risk through spatial extent (EXT), ecosystem sensitivity or protection status (ECO), biodiversity impact (BIO), reversibility or duration (REV), substance type or intrinsic hazardousness (SUS), and concentration or volume (CONC). The final index is calculated as follows:

$${\text{IDPAm}}_{\text{base}}=\left(W_{j}\times \mathrm{LC}\right)+\left(W_{e}\times \mathrm{EC}\right)$$

T=min(T1+T2+T3+T4,2)

$$\text{IDPAm}={\text{IDPAm}}_{\text{base}}+T$$

where the default weights are $W_{j}=0.4$ and $W_{e}=0.6$ . The ordinary weighted base scale ranges from 0 to 10, while the capped trigger adjustment may add up to 2 points in qualified cases. In the pilot, T1 represents critically endangered species, T2 maximum-protection areas, T3 serious risk to human health, and T4 extremely hazardous substances. The resulting calculation assesses material legal-ecological seriousness as structured by the model. It does not establish criminal liability, prove the statutory elements of an offence, determine causation or culpability, demonstrate evidential sufficiency, predict conviction, or prescribe a sentence.

The pilot’s EC values were calculated from the six-variable matrix using the original conservative coding protocol. In the reconciled dataset, all ecological fields are recorded numerically on the 0–3 scale; the dataset does not yet preserve separate codes for missing information, insufficient or contested evidence, or non-applicability. A score of zero in the existing pilot should therefore be interpreted cautiously. It records the final reconciled application of the original coding anchors, but it does not permit readers to determine systematically whether a low score reflects a documented absence or minimal level of the relevant feature, incomplete information in the published judgment, evidential uncertainty, or limited sectoral applicability of the variable. This limitation is particularly relevant in heterogeneous cases where, for example, CONC may be less informative than BIO, ECO, or REV.

For reproducibility, the reconciled case-level dataset preserves the final raw variable scores, standardised LC and EC values, T1–T4 trigger activations, the capped trigger value, final IDPAm score, interpretive band, judicial outcome, court, year, sector, and procedural disposition where available. IDPAm was calculated using unrounded standardised LC and EC values derived from the raw component scores. Rounded values are reported only for presentation purposes. These materials permit reproduction of the reported calculations, descriptive statistics, figures, consistency checks, and sensitivity analyses. They do not yet permit full independent replication of every original coding decision, because coder-specific pre-reconciliation values, source-by-variable evidential references, systematic uncertainty codes, and detailed trigger rationales are not retained in the currently available pilot dataset.

The Enforceability module (IDPAe) is not applied in this pilot phase. This omission is methodologically justified because publicly available judgments rarely provide sufficiently uniform, detailed, and comparable access to limitation issues, evidential nullities, expert disagreement, chain-of-custody questions, causal and attributional problems, conformity dynamics, or other procedural elements necessary for reliable IDPAe coding. The present empirical application therefore concerns IDPAm alone. It can describe the distribution of material legal-ecological seriousness in the selected corpus, but it cannot explain why individual cases resulted in conviction, acquittal, or conformity-based resolution. Future validation should operationalise IDPAe through a distinct set of transparently defined procedural and evidential variables, preferably using fuller case files or sources that provide more systematic access to the relevant information.

### 5.3 Descriptive results

The 36 coded cases generated IDPAm values ranging from 0.7 to 9.3, with a concentration in the lower and intermediate ranges of the scale. The mean IDPAm was 4.18, the median was 3.70, the standard deviation was 2.14, and the interquartile range extended from 3.00 to 5.78. These values describe the distribution of material legal-ecological seriousness generated by the reconciled coding of the selected pilot corpus. They should not be interpreted as estimates of the distribution, prevalence, or severity of environmental crime in Spain more generally.

Using the indicative interpretive bands proposed by LEDAT, 19 cases (52.8%) fall within the 0–3.9 range, corresponding to a predominantly administrative profile; 13 cases (36.1%) fall within the 4.0–6.9 range, corresponding to the grey area of debatable criminal significance; and 4 cases (11.1%) reach the ≥7.0 range, corresponding to high material criminal significance. Thus, 32 of the 36 cases (88.9%) fall below the high-significance band. This concentration should be interpreted descriptively rather than inferentially. It indicates that, in this selected pilot corpus, the model produces most scores in low or intermediate material-seriousness ranges rather than in the exceptional high-significance range. It does not demonstrate that most environmental offences, judicial decisions, or ecological harms fall within those ranges.

Figure 2 illustrates the overall distribution of IDPAm scores across the analysed sample. The histogram incorporates the indicative interpretive bands proposed by LEDAT and highlights the concentration of cases within the predominantly administrative and intermediate ranges of the index. Given the small sample size and the non-normal distribution of IDPAm values, the figure should be interpreted together with the median and interquartile range rather than through the mean alone.

The shaded area represents the interpretive grey zone proposed by the LEDAT model. Dashed vertical lines indicate the lower boundary of the grey zone and the high-significance threshold; solid and dotted vertical lines indicate the sample mean and median, respectively. The bands shown are heuristic descriptive categories and do not establish legal thresholds, criminal liability, evidential sufficiency, or sentencing outcomes.

The distribution also illustrates why the interpretive bands require caution, especially in cases close to a boundary. A score in the grey area does not establish that criminal liability is uncertain, just as a score in the predominantly administrative range does not establish that the case should have been handled administratively. The bands organise the material legal-ecological profile generated by the framework. They are intended to facilitate scrutiny of the underlying variables, evidential sources, and modelling assumptions, rather than to substitute for the legal assessment required in the individual case.

Within the pilot corpus, cases ending in conviction displayed higher average IDPAm values than acquittals. The 20 convictions had a mean IDPAm of 4.83 and a median of 4.50, compared with a mean of 3.38 and a median of 3.50 among the 16 acquittals. This descriptive difference should not be interpreted as evidence of predictive capacity, causal influence, statistical classification performance, or external validation. IDPAm incorporates a Legal Component that partly records the normative configuration of the case, while judicial outcomes are also shaped by causation, attribution, evidential sufficiency, procedural validity, prosecutorial strategy, and the form of case resolution.

The distinction between contested and conformity-based convictions is particularly important. Of the 20 convictions, 9 were contested convictions and 11 were conformity-based convictions. Contested convictions had a mean IDPAm of 4.66 and a median of 4.00. Conformity-based convictions had a mean IDPAm of 4.96 and a median of 4.70. The corresponding ranges were 1.7–8.0 for contested convictions and 3.0–9.3 for conformity-based convictions, while acquittals ranged from 0.7 to 8.3. The distributions therefore overlap substantially: some acquittals display higher IDPAm values than some contested or conformity-based convictions.

These comparisons are descriptive features of a small and procedurally heterogeneous corpus. They do not demonstrate that IDPAm predicts judicial outcome. The slightly higher mean and median among conformity-based convictions reinforce the need to retain that group as analytically distinct. A conformity-based conviction may reflect acceptance of responsibility, procedural strategy, evidential negotiation, or sentencing considerations that cannot be reconstructed fully from the publicly available judgment. Conversely, an acquittal may coexist with a materially serious legal-ecological profile where causation, attribution, procedural validity, or evidential sufficiency is not established to the criminal standard.

**Figure 3.**
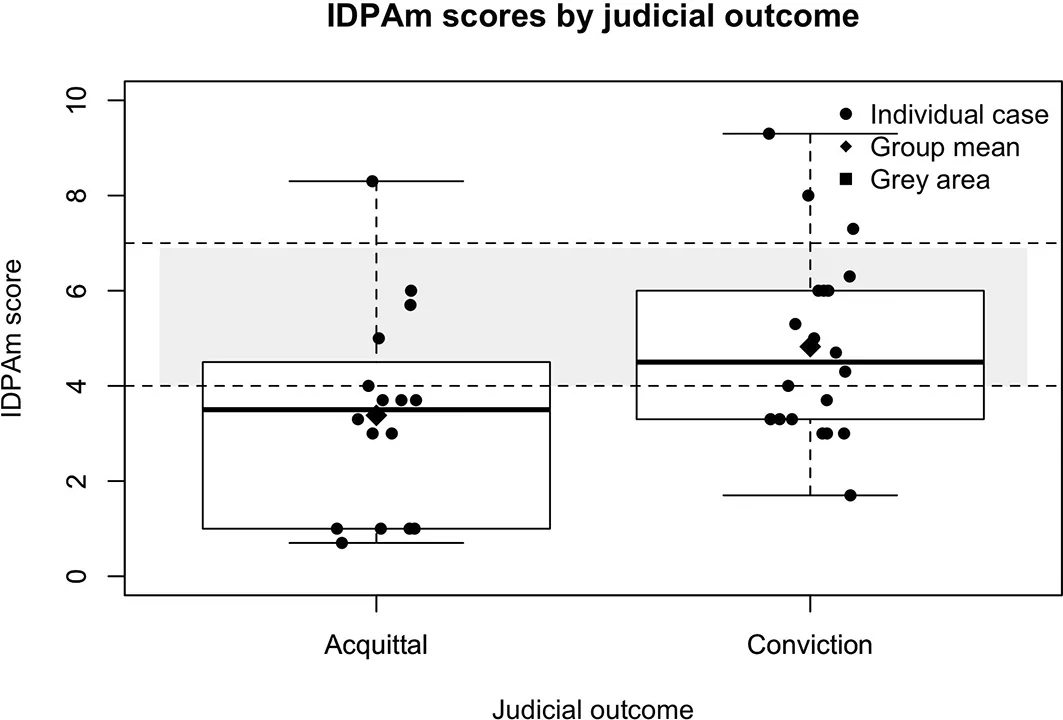
IDPAm scores by judicial outcome type. The figure compares the distribution of IDPAm values in acquittals and convictions and illustrates the substantial overlap between both groups. The shaded area represents the interpretive grey zone. Points represent individual cases; diamonds indicate group means.

Figure 3 compares IDPAm values across acquittals, contested convictions, and conformity-based convictions. Presenting three outcome categories, rather than a simple conviction/acquittal dichotomy, makes the procedural composition of the corpus visible and reduces the risk of treating all convictions as equivalent adjudicative outcomes.

The figure compares the distribution of IDPAm values across acquittals, contested convictions, and conformity-based convictions. The shaded area represents the interpretive grey zone. Points represent individual cases, and diamonds indicate group means. The figure is descriptive only: it illustrates overlap and variation across observed outcome types and does not test prediction, causation, statistical significance, or external validity.

The findings must also be read in light of the scope and current limitations of the pilot coding. The reconciled dataset permits computational reproduction of the descriptive statistics, figures, and sensitivity analyses. It does not yet preserve coder-specific pre-reconciliation values, source-by-variable evidential references, systematic uncertainty codes, or complete case-level trigger rationales. In addition, IDPAe is not systematically applied because the published judgments do not provide sufficiently uniform access to the complete evidential and procedural record. The present analysis can therefore describe the relationship between IDPAm profiles and observed judicial outcomes, but it cannot explain why an individual case resulted in acquittal, contested conviction, or conformity-based conviction.

Future applications should preserve coder-specific values before reconciliation, report formal inter-coder agreement measures, distinguish documented low scores from missing, insufficient or contested, and non-applicable ecological information, document trigger activation and anti-duplication assessments at case level, test plausible alternative band boundaries, and apply IDPAe systematically. These steps are necessary before stronger claims can be made about the relationship between material seriousness, enforceability, and judicial outcome.

### 5.4 Sectoral breakdown

The 36 valid cases are distributed across 11 sectoral labels in the coding database (see Table 6): noise pollution ( *contaminación acústica*), noise pollution with injuries ( *contaminación acústica + lesiones*), water discharges ( *vertidos*), waste ( *residuos*), waste and emissions ( *residuos + emisiones*), extractions ( *extracciones*), emissions ( *emisiones*), atmospheric pollution ( *contaminación atmosférica*), damage to a protected area ( *daños a espacio protegido*), natural resources ( *recursos naturales*), and urban planning ( *urbanismo*). These labels are retained to preserve fidelity to the coding dataset. They are descriptive categories rather than mutually exclusive legal classifications or standardised ecological sectors.

**Table 6.** Sectoral distribution of the pilot sample and descriptive IDPAm values.

Material sector	n	Convictions	Mean IDPAm	SD	Range	Conviction rate (%)
Noise Pollution	12	6	2.63	1.38	0.7–5.0	50.0
Water discharges	6	3	5.40	1.80	3.0–8.0	50.0
Waste	4	4	5.65	1.54	3.7–7.3	100.0
Extractions	4	1	4.43	3.02	1.0–8.3	25.0
Air Pollution	2	1	7.65	2.33	6.0–9.3	50.0
Emissions	2	2	4.65	0.49	4.3–5.0	100.0
Noise Pollution + Injuries	2	2	2.35	0.92	1.7–3.0	100.0
Waste + Emissions	1	1	6.00	—	6.0–6.0	100.0
Urban Planning	1	0	4.00	—	4.0–4.0	0.0
Natural Resources	1	0	3.70	—	3.7–3.7	0.0
Damage to a protected area	1	0	3.30	—	3.3–3.3	0.0

The sectoral breakdown should be interpreted with particular caution. Several categories contain only one or two judgments, and the corpus was not designed or stratified to provide representative coverage of particular offence sectors. The figures reported below are therefore descriptive properties of the selected pilot dataset. They do not support statistical inference, estimates of sector-level prevalence, comparisons of conviction rates, or conclusions about the relative real-world seriousness of different forms of environmental offending.

In Table 6, sectoral means, standard deviations, ranges, and conviction rates are reported descriptively only. Standard deviations are not reported for sectors represented by a single observation. Conviction rates should be interpreted as procedural descriptors of the cases retained in each category, not as measures of enforcement effectiveness, evidential strength, sectoral criminality, or the likelihood that a similar case would result in conviction.

The largest sectoral group in the pilot corpus is noise pollution, with 12 cases and a mean IDPAm of 2.63. If the two cases labelled noise pollution with injuries are considered alongside that group, noise-related cases account for 14 of the 36 judgments. This indicates that noise-related litigation is prominent within the selected corpus, but its prominence cannot be extrapolated to Spanish environmental criminal litigation generally. The relatively low IDPAm values in these cases should not be interpreted as showing that noise pollution is inherently less serious, lacks ecological relevance, or is appropriately treated only through administrative law. They reflect the specific factual, legal, ecological, evidential, and procedural profiles of the judgments included in the pilot.

Water-discharge cases constitute the second largest category, with six judgments and a mean IDPAm of 5.40. Waste cases have a mean IDPAm of 5.65, while extraction-related cases display substantial internal variation, ranging from 1.0 to 8.3. Atmospheric pollution has the highest sectoral mean, 7.65, but this value is based on only two observations. These figures illustrate variation within the pilot corpus; they do not establish a hierarchy of sectoral seriousness. In particular, an arithmetic comparison of sectoral means cannot determine whether one sector creates more severe ecological harm in practice, because the cases differ in legal configuration, ecological setting, evidential record, procedural history, and the relative relevance of individual LEDAT variables.

Sectoral heterogeneity also affects coding comparability. CONC and SUS may be particularly informative in discharge, waste, emission, or atmospheric-pollution cases. By contrast, ECO, BIO, and REV may carry greater interpretative relevance in cases concerning protected areas, natural resources, species, habitat degradation, or extraction. The common EC scale supports structured descriptive comparison only to the extent that the coding record identifies the evidential basis of each variable and distinguishes a documented low score from missing, insufficient or contested information, and non-applicability. The present pilot does not yet preserve those distinctions systematically for every ecological variable. Sectoral comparisons must therefore be read as exploratory descriptions of the reconciled coding dataset, not as fully standardised intersectoral measurements.

The sectoral breakdown remains useful for the limited purpose of the pilot. It demonstrates that the LEDAT matrix can be applied across a heterogeneous group of environmental criminal judgments and makes visible the range of legal-ecological profiles represented in the corpus. It also identifies areas requiring more rigorous future testing: larger sector-specific samples, clearer rules for variable relevance and non-applicability, fuller evidential documentation, formal inter-coder reliability analysis, separate consideration of contested and conformity-based outcomes, and systematic inclusion of IDPAe. Those steps are necessary before the framework can support stronger claims about consistency across offence sectors or the relationship between material seriousness, enforceability, and judicial outcome.

### 5.5 Interpretation of the pilot findings

The empirical application suggests three provisional findings.

The empirical application supports three provisional findings concerning the pilot corpus and the present stage of LEDAT’s development.

First, the pilot indicates that the framework can be operationalised in heterogeneous first-instance environmental-crime judgments. The reconciled coding dataset generates IDPAm values ranging from 0.7 to 9.3 and produces a structured descriptive ordering of cases across lower, intermediate, and higher material legal-ecological profiles. This result supports a limited claim of operational feasibility: the framework can be applied to real judicial material and can organise selected legal and ecological features in a common analytical structure. It does not establish that all variables can be coded with equal completeness, certainty, or sectoral relevance from publicly available judgments. In particular, the pilot dataset does not yet distinguish systematically between a documented low value, missing information, insufficient or contested evidence, and non-applicability. The feasibility finding must therefore be interpreted alongside the limits of the available judicial record and the current coding protocol.

Secondly, the pilot produces a concentration of scores below the high material criminal significance band. Nineteen cases fall within the predominantly administrative profile, 13 within the grey area, and 4 within the high-significance range. This distribution shows how the proposed model orders the 36 selected judgments under its current scoring, weighting, trigger, and band assumptions. It does not demonstrate that Spanish environmental criminal litigation generally operates within a broad threshold zone, nor that the interpretive bands correspond to independently validated legal or ecological thresholds. The contribution of LEDAT is not to eliminate the indeterminacy of environmental criminal thresholds. It is to make the legal-ecological features, evidential assumptions, and modelling decisions underlying the descriptive placement of cases more visible, auditable, and open to challenge.

Thirdly, the outcome comparison confirms that material legal-ecological seriousness and judicial result do not correspond in a one-to-one manner. Convictions display a higher mean and median IDPAm than acquittals in the pilot corpus, but the distributions overlap substantially. The more detailed distinction among 16 acquittals, 9 contested convictions, and 11 conformity-based convictions reinforces that point. Some acquittals display higher IDPAm values than some convictions, and the mean IDPAm of conformity-based convictions is slightly higher than that of contested convictions. These observations are descriptive only. They do not demonstrate that a higher IDPAm score predicts conviction, that material seriousness causes a particular judicial outcome, or that judicial outcome validates the index.

This result illustrates why the separation between IDPAm and IDPAe is necessary. The pilot records material legal-ecological seriousness through the Legal Component, Ecological Component, and capped trigger adjustment. It does not systematically code the procedural and evidential conditions that may explain divergence between material seriousness and outcome. A materially serious case may fail because causation, attribution, expert evidence, procedural validity, or another requirement of criminal adjudication is not established. Conversely, a case with moderate material seriousness may result in conviction where the factual and legal issues are comparatively straightforward or where the case is resolved by conformity. The current pilot cannot determine which of those mechanisms explains any individual outcome because IDPAe is not yet operationalised and publicly available judgments do not provide a sufficiently complete and uniform procedural record.

The present findings should therefore be interpreted in terms of descriptive coherence, operational feasibility, and structured case ordering. The pilot does not establish predictive validity, causal relationships, statistical classification performance, coder-independent reproducibility, inferential generalisability, or external validation against an independent legal or ecological benchmark. Its results are also conditional on the current coding architecture, including the treatment of ecological information, the default 0.4/0.6 weighting structure, the T1–T4 trigger operationalisation, the trigger cap, and the proposed interpretive bands.

In this respect, the future development of the model is also relevant to the broader European context. Directive (EU) 2024/1203 requires Member States to strengthen specialised training, coordination, national strategies, and statistical systems, and to transmit comparable data in a common format after the Commission establishes the standardised reporting framework. A structured methodology such as LEDAT may be useful for empirical research and for organising transparent legal-ecological reasoning within that wider context. It cannot, however, be treated as a harmonised European scoring system or as a substitute for the legal standards, evidential safeguards, and judicial assessment applicable in each Member State and individual case.

The methodological ambition of LEDAT is therefore limited. It is not to transform environmental criminal adjudication into a purely quantitative exercise, but to provide a structured and explainable composite framework through which selected legal and ecological considerations can be documented, compared, audited, and contested under conditions of legal, ecological, and evidential complexity. Its value depends on the continued refinement of coding rules, case-level evidential traceability, formal inter-coder reliability assessment, transparent trigger documentation, sensitivity analysis, sector-sensitive application, and the separate operationalisation of IDPAe.

### 5.6 Sensitivity analysis of weighting structure and trigger adjustment

Given the formative and composite nature of LEDAT, its weighting structure, trigger adjustment, and interpretive bands should not be understood as statistically fixed parameters. They are transparent modelling choices requiring exploratory assessment. A preliminary sensitivity analysis was therefore conducted to examine whether plausible alternative assumptions produced substantial alterations in the relative ordering of cases or in their allocation to the proposed interpretive bands.

The analysis considered three distinct sources of potential variation. First, it examined alternative legal-ecological weighting structures. Secondly, it assessed the effect of removing the capped trigger adjustment. Thirdly, it tested the sensitivity of case classifications to plausible alternative boundaries for the interpretive bands. These analyses serve different purposes and should not be conflated. Weighting scenarios test the ordinary integration of LC and EC; the no-trigger scenario tests the contribution of qualified ecological escalation; and band-boundary scenarios test the descriptive classification consequences of the proposed thresholds without altering the underlying IDPAm values.

In addition to the default configuration ( $W_{j}=0.4;W_{e}=0.6$ ), two alternative weighting scenarios were examined: balanced weighting ( $W_{j}=0.5;W_{e}=0.5$ ) and a more ecology-prioritised configuration ( $W_{j}=0.3;W_{e}=0.7$ ). Alternative weighting scenarios were calculated using the same standardised LC and EC values and the same capped trigger adjustment. The trigger system was then examined in a restricted no-trigger scenario in which T was set to zero for every case.

In the present pilot, the capped trigger adjustment was derived from the four operational trigger fields: T1, critically endangered species; T2, area of maximum ecological protection; T3, serious risk to human health; and T4, extremely hazardous substance. The raw trigger sum was 0 in 12 cases, 1 in 14 cases, 2 in 5 cases, and 3 in 5 cases. The cap therefore affected 5 of the 36 cases. These results demonstrate the practical relevance of the cap, but they do not by themselves validate individual trigger activations or the anti-duplication protocol. Full future applications should preserve the evidential basis, qualitative rationale, and anti-duplication assessment for every trigger recorded at case level.

The exploratory comparison suggests that the ordinal ordering of cases remains highly stable under the two alternative weighting configurations. Compared with the default 0.4/0.6 structure, the balanced 0.5/0.5 configuration produced a Spearman rank correlation of 0.981 and a Kendall rank correlation of 0.936, with two cases changing interpretive band. The more ecology-prioritised 0.3/0.7 configuration produced a Spearman rank correlation of 0.989 and a Kendall rank correlation of 0.950, also with two cases changing interpretive band. In both alternative weighting scenarios, the aggregate band distribution remained unchanged: 19 cases in the predominantly administrative range, 13 in the grey area, and 4 in the high material criminal significance band. The observed band changes therefore concerned movement by individual borderline cases between adjacent categories, rather than a change in the aggregate distribution.

The removal of triggers produced a more visible descriptive effect. Although the ordinal association with the default configuration remained high, with a Spearman rank correlation of 0.940 and a Kendall rank correlation of 0.861, 8 cases changed interpretive band. The aggregate distribution shifted from 19/13/4 under the default model to 24/11/1 under the no-trigger scenario. This result indicates that the trigger mechanism contributes materially to classification in some cases involving qualified ecological features. At the same time, the capped design limits that contribution: no case can receive more than 2 additional points, regardless of the number of qualifying trigger conditions.

The proposed band boundaries were also tested through two symmetrical alternative scenarios. The default boundaries classify scores below 4.0 as predominantly administrative, scores from 4.0 to below 7.0 as grey-area cases, and scores of 7.0 or above as high material criminal significance. The first alternative lowers both thresholds by 0.5 points, producing boundaries of 3.5 and 6.5. The second raises both thresholds by 0.5 points, producing boundaries of 4.5 and 7.5. These scenarios do not claim to identify optimal thresholds. They provide a transparent illustration of how modest and plausible changes in the proposed heuristic boundaries affect descriptive case classification.

Under the lower-boundary scenario (3.5/6.5), four cases moved from the predominantly administrative band to the grey area. The distribution changed from 19/13/4 to 15/17/4. Under the higher-boundary scenario (4.5/7.5), four cases changed band: three cases moved from the grey area to the predominantly administrative range, while one case moved from the high-significance band to the grey area. The resulting distribution was 22/11/3. As expected, these scenarios do not alter the underlying case ranking because they do not modify the IDPAm score itself. They show, however, that the classification of a limited number of cases depends on the location of the chosen band boundaries, particularly near the default cut-points.

The pilot therefore supports three limited exploratory conclusions. First, the relative weighting of the Legal and Ecological Components appears reasonably stable within the narrow range of alternative assumptions tested. Secondly, the trigger mechanism is substantively consequential for the assignment of some cases to interpretive bands and should not be treated as an auxiliary or merely illustrative feature of the model. Thirdly, the proposed bands are sensitive to modest shifts in their boundaries for a small group of borderline cases. This confirms that the bands should remain heuristic, non-binding, and subordinate to examination of the underlying legal, ecological, and evidential profile of each case.

For empirical purposes, the sensitivity analysis improves the transparency of the pilot by showing how much of the resulting descriptive classification depends on weighting assumptions, trigger treatment, and band boundaries (see Table 7). It does not amount to statistical calibration, external validation, proof of optimal weights, validation of the trigger protocol, or confirmation that the proposed interpretive bands correspond to legal or ecological thresholds recognised independently of the model.

**Table 7.** Exploratory sensitivity check of weighting structure and trigger adjustment.

Configuration	Spearman correlation with default	Kendall correlation with default	Band changes	Band distribution	Main observed variation
Default weights and bands: 0.4/0.6	1.000	1.000	0	19/13/4	Baseline
Balance weights: 0.5/0.5	0.981	0.936	2	19/13/4	Minor movement in borderline cases
Ecology-prioritised weights: 0.3/0.7	0.989	0.950	2	19/13/4	Minor movement in cases with divergent legal and ecological scores
No triggers: 0.4/0.6; T = 0	0.940	0.861	8	24/11/1	Reduced qualitative escalation in trigger-activated cases
Lower band boundaries: 3.5/6.5	Not applicable: scores unchanged	Not applicable: scores unchanged	4	15/17/4	Four cases move from predominantly administrative to grey area
Higher band boundaries: 4.5/7.5	Not applicable: scores unchanged	Not applicable: scores unchanged	4	22/11/3	Three cases move from grey area to predominantly administrative; one moves from high significance to grey area

In Table 7 band distribution is reported in the order predominantly administrative/grey area/high material criminal significance. “Band changes” refers to the number of individual cases assigned to a different interpretive band relative to the default configuration. For the two band-boundary scenarios, rank correlations are not reported because the underlying IDPAm values and ordinal ranking remain unchanged; only the descriptive classification cut-points vary.

Because the present empirical application is exploratory and based on a limited sample ( n=36 ), these observations should not be interpreted as definitive statistical validation. Their function is more modest: to assess the transparency and descriptive robustness of LEDAT under a limited set of plausible alternative parameter configurations. Future research should evaluate a broader range of specifications using larger and more diverse datasets, formally assess coding reliability, document all trigger decisions at case level, and test whether any interpretive-band configuration displays acceptable coherence when assessed against independently specified legal and ecological benchmarks.

### 5.7 Limitations and next steps

The pilot nature of the empirical exercise should be stated clearly. The contribution of this study lies in methodological feasibility, structured descriptive case ordering, and the transparent examination of selected modelling assumptions. It does not lie in statistical generalisation, prediction of judicial outcomes, causal explanation, or definitive validation of LEDAT as a decision instrument. The following limitations qualify the interpretation of all pilot findings.

The first limitation concerns sample size. The final corpus of 36 judgments is sufficient for an exploratory application of the coding framework, but not for robust inferential modelling, statistical calibration of weights, validation of interpretive thresholds, or reliable estimation of sectoral patterns. The descriptive distributions and sensitivity results reported in this article are useful for methodological appraisal, but they cannot support strong generalisations about Spanish environmental criminal enforcement as a whole. Larger datasets will be necessary to examine the stability of the model across different years, courts, offence types, territorial contexts, and legal systems.

The second limitation concerns case selection and representativeness. The Tirant Prime search strategy was systematic within the selected database, period, legal search terms, and procedural filters, but it remains dependent on indexing practices, search terminology, procedural classification, and the availability of written judgments. The final corpus is therefore shaped both by the database architecture and by judicial and enforcement practices. It should be understood as a filtered jurisprudential sample of codable first-instance judgments, rather than as a complete census of Spanish environmental criminal litigation between 2020 and 2025. It cannot be used to estimate the prevalence, detection, prosecution, conviction, or real-world distribution of environmental harm or environmental offending.

The third limitation concerns the procedural composition of the sample. The 36 decisions are first-instance judgments in the procedural sense, but they were issued by different trial courts under the applicable jurisdictional rules: 26 by Provincial Courts and 10 by Criminal Courts. No High Court of Justice decision was retained in the final corpus. This composition is compatible with the research design, but it should not be assumed that the cases are procedurally homogeneous. Court type, procedural route, available evidence, legal complexity, and the form of resolution may affect the content and level of detail of the published judgment.

The fourth limitation concerns the coding process and the absence of formal inter-coder reliability coefficients. The cases were coded independently before reconciliation, but the currently available pilot materials do not preserve a complete set of coder-specific pre-reconciliation scores. Formal agreement statistics therefore cannot be calculated retrospectively from the reconciled dataset. Qualitative observations made during reconciliation should not be treated as formal estimates of reliability. Future applications should retain the complete coder-specific records and report weighted Cohen’s kappa for ordinal variables where appropriate, Krippendorff’s alpha where missingness or coding design makes it preferable, and intraclass correlation coefficients based on absolute agreement for LC, EC, IDPAm base score, and final IDPAm. Agreement should also be assessed separately for trigger variables and interpretive-band assignment.

The fifth limitation concerns the treatment of incomplete, uncertain, and non-applicable ecological information. The reconciled pilot dataset records EC variables numerically from 0 to 3, but it does not systematically distinguish among a documented low or absent ecological feature, missing information, insufficient or contested evidence, and non-applicability of a variable to a particular offence profile. This is particularly relevant in a heterogeneous corpus. For example, CONC may be central to a discharge, waste, or emission case but less informative in a protected-species, habitat, or land-use case; BIO or ECO may have the opposite pattern. The original conservative coding approach made it possible to generate final scores from the public judgments, but it may also obscure whether lower values reflect documented ecological conditions, limited judicial information, evidential uncertainty, or sectoral non-applicability. Future versions of the coding protocol should preserve these categories separately and specify the calculation rule applicable when a variable is non-applicable or cannot be reliably coded.

The sixth limitation concerns the non-inclusion of IDPAe. Publicly available judgments rarely provide sufficiently uniform, complete, and comparable information on limitation periods, evidential nullities, investigative constraints, expert disagreement, chain-of-custody issues, causal and attributional difficulties, prosecutorial strategy, or the dynamics of conformity-based resolutions. As a result, the present pilot can describe the relationship between IDPAm profiles and observed judicial outcomes only imperfectly. It cannot explain why a particular materially serious case resulted in acquittal, why a moderate case ended in conviction, or which procedural or evidential factors account for divergence between material seriousness and the final outcome.

This limitation is particularly important because 11 of the 20 convictions in the corpus were conformity-based, while 9 were contested convictions. These two forms of conviction should not be treated as methodologically equivalent. A conformity-based resolution may reflect acceptance of responsibility, procedural strategy, evidential negotiation, sentence-related considerations, or factors not fully visible in the published judgment. The distinction between acquittals, contested convictions, and conformity-based convictions is therefore reported descriptively in this pilot, but it cannot serve as an external validation test for IDPAm.

The seventh limitation concerns offence and sectoral heterogeneity. The corpus includes noise-related cases, water discharges, waste, waste-emission combinations, extractions, emissions, atmospheric pollution, protected-area damage, natural-resource cases, and urban-planning matters. These categories differ in ecological dynamics, legal framing, availability of technical evidence, procedural architecture, and the relevance of particular LEDAT variables. Such heterogeneity is useful for testing whether the matrix can be applied to diverse judicial material. It does not, however, permit strong cross-sectoral conclusions, particularly where several labels contain only one or two observations. Sectoral means and conviction rates are descriptive characteristics of the pilot corpus, not indicators of the comparative real-world seriousness, frequency, or enforceability of different forms of environmental offending.

An eighth limitation concerns the formative composite structure of the model. LEDAT intentionally integrates heterogeneous legal and ecological dimensions rather than treating them as reflective indicators of a single latent construct. It does not therefore seek to demonstrate internal consistency, latent unidimensionality, or psychometric reliability in the classical sense. Its methodological appraisal should focus instead on conceptual justification of the selected variables, transparency of ordinal score anchors, coder agreement, evidential traceability, sensitivity to alternative modelling assumptions, and external coherence across larger and more diverse datasets. The 0.4/0.6 weighting structure, trigger cap, trigger operationalisation, and interpretive bands remain provisional and theory-informed choices rather than statistically calibrated parameters.

A ninth limitation concerns trigger documentation and implementation. The pilot operationalises four trigger fields: T1 for critically endangered species, T2 for areas of maximum ecological protection, T3 for serious risk to human health, and T4 for extremely hazardous substances. The cumulative adjustment is capped at 2 points. Trigger removal changes the interpretive band of eight cases and reduces the number of high-significance cases from four to one. This confirms that triggers are methodologically consequential. Yet the currently available pilot dataset does not retain a complete case-level record of the factual source, evidential basis, distinct qualitative rationale, and anti-duplication review for every activation. The trigger-related sensitivity findings are therefore computationally reproducible from the reconciled values, but not fully reproducible as original coding decisions. Future applications should preserve labelled trigger categories, source references, anti-duplication assessments, raw trigger sums, capped values, and coder-specific trigger assignments before reconciliation.

These limitations do not undermine the limited contribution of the pilot. They define the conditions under which it should be interpreted. The study demonstrates that a reconciled LEDAT coding matrix can be applied to a bounded corpus of real judicial decisions, generate a structured descriptive ordering of material legal-ecological seriousness, and make the consequences of alternative weighting, trigger, and band-boundary assumptions visible. It does not demonstrate that LEDAT predicts judicial outcomes, establishes criminal liability, replaces discretionary legal reasoning, provides a biophysically exact measure of environmental damage, or supplies a universally applicable criminal threshold.

The next stage of research should therefore focus on replication and refinement rather than stronger substantive claims. Future work should expand the corpus temporally, geographically, and sectorally; apply the protocol across additional jurisdictions; preserve full coder-specific pre-reconciliation records; report formal inter-coder agreement; implement separate codes for zero, missing, insufficient or contested, and non-applicable information; create a source-by-variable coding annex; document trigger activation and anti-duplication decisions at case level; test broader ranges of weighting, trigger, and band-boundary assumptions; distinguish acquittals, contested convictions, and conformity-based convictions; and operationalise IDPAe through a separate set of procedural and evidential variables. Only after those steps can the framework be evaluated more rigorously for coding reliability, cross-sectoral coherence, external consistency, and the relationship between material seriousness, enforceability, and observed judicial outcomes.

Cross-jurisdictional application will be particularly relevant in the post-transposition context of Directive (EU) 2024/1203. The Directive requires Member States to strengthen capacity, training, coordination, and statistical reporting in environmental criminal enforcement. LEDAT may contribute to empirical research and transparent legal-ecological reasoning within that context, but it should not be treated as a harmonised European scoring system or a substitute for national criminal-law rules, evidential safeguards, or judicial assessment.

For computational reproducibility, the reconciled case-level dataset, methodological appendix, and analysis scripts will be made available as supplementary material, subject to the limitations described in the Data Availability Statement and Code Availability Statement. The available materials should, at minimum, preserve case identifier; court; date or year; sectoral label; judicial outcome; procedural disposition, including conformity where available; TP, INT, AGR, REC, EXT, ECO, BIO, REV, SUS, and CONC; raw and standardised component scores; T1–T4 values; raw trigger sum; capped trigger value; final IDPAm; interpretive band; and the scripts required to reproduce the reported calculations and descriptive analyses. To support full coding reproducibility in future studies, the supplementary materials should additionally provide source-by-variable evidential references, uncertainty and non-applicability codes, trigger rationales, anti-duplication reviews, coder-specific pre-reconciliation values, reconciliation notes, and formal reliability statistics. If licensing, confidentiality, or editorial restrictions prevent disclosure of any component, the materials should preserve the maximum level of derived coding information compatible with those constraints.

## 6. Implications for enforcement, remedies, and empirical monitoring

LEDAT is designed as a transparent argument-structuring and empirical coding framework, not as a binding metric or automated decision system. Its purpose is to organise the assessment of material legal-ecological seriousness in a manner that can be explained, reviewed, challenged, and, subject to the availability of the underlying coding record, reproduced. In line with Directive (EU) 2024/1203, which requires adequate resources, specialised training, coordination, national strategies, and comparable statistics, LEDAT may be relevant to prosecutors, experts, courts, defence counsel, and empirical researchers only if its operation remains transparent, evidence-based, and open to contradiction.

The practical use of LEDAT must respect three basic limits. First, the output of the model cannot replace legal classification, factual assessment, evidential evaluation, or judicial reasoning. It does not establish the statutory elements of an offence, prove causation, demonstrate culpability, discharge the burden of proof, determine evidential sufficiency, or predict the outcome of proceedings. Secondly, IDPAm and IDPAe must remain analytically separate. IDPAm concerns material legal-ecological seriousness as structured through the Legal Component, Ecological Component, and capped trigger adjustment. IDPAe concerns procedural and evidential viability. A high IDPAm score therefore cannot, by itself, justify prosecution, conviction, a particular remedial measure, or a more severe sentence. Thirdly, any practical use of LEDAT must be accompanied by a source-by-variable explanation identifying the factual and evidential basis for each score, any uncertainty affecting the coding, every trigger activation, and the application of the anti-duplication rule.

A final IDPAm value without an auditable coding trail would be methodologically inadequate and difficult to reconcile with the procedural safeguards of criminal adjudication. Traceability is particularly important because environmental criminal cases often depend on complex technical and expert evidence. LEDAT may be used, where appropriate, as a structured format for organising and making explicit the reasoning contained in expert assessments. It cannot substitute for expert judgment, scientific explanation, or adversarial examination of evidence. Each assigned value should be linked to an identifiable source, such as an expert report, environmental inspection, administrative file, laboratory result, judicial finding of fact, official map, protected-area classification, species-conservation listing, toxicological assessment, or other admissible material. Where the evidence is incomplete, uncertain, or contested, the record should state that limitation rather than conceal it behind a numerical output.

The same logic applies to prosecutorial assessment. LEDAT may assist in identifying and documenting the ecological and legal features that warrant closer threshold analysis, in structuring requests for further technical evidence, or in clarifying the factual basis of an expert instruction. It cannot determine whether charges must be filed, whether the elements of an offence are satisfied, whether available evidence reaches the required standard, or whether prosecution is in the public interest. Its appropriate role is to make the relevant considerations visible and contestable, not to replace the legal and evidential assessment required before an accusation is brought.

In judicial reasoning, LEDAT may provide a transparent framework through which the parties and the court can identify the dimensions of material legal-ecological seriousness that are being relied upon or disputed. Its use must remain subordinate to the court’s individualised assessment of the facts, the applicable criminal provision, causation, culpability, proof, proportionality, and procedural guarantees. An IDPAm score may support explanation of why certain ecological features merit legal attention, but it cannot be treated as an autonomous basis for conviction, acquittal, or a finding that the criminal threshold has been met. The court must remain able to accept, reject, or qualify the relevance of every coded factor through reasoned judicial decision.

The distinction is equally relevant to defence practice. A transparent coding matrix may allow defence counsel to identify and contest the factual basis, evidential source, legal relevance, score anchor, trigger activation, or anti-duplication assessment applied to a particular variable. This potential for variable-by-variable contradiction is one reason why LEDAT should remain fully explainable and accessible to the parties. It does not reverse the burden of proof, require the defence to disprove an index, or alter the presumption of innocence. The prosecution remains responsible for establishing the elements of the offence according to the applicable legal standard.

The same limits apply to remedies and sentencing. LEDAT may help structure reasoning about restoration, compensation, remediation, or ancillary measures by identifying the ecological dimensions that may be relevant to their scope. Where damage is reversible, the ecological variables may assist in describing the intensity, duration, and territorial reach of restoration needs. Where damage is irreversible or only partially reversible, they may support a more explicit discussion of compensation, substitute restoration, or ecosystem-service loss. However, remedial measures must remain governed by the applicable legal framework, the evidence in the individual case, and proportionality. They should never be imposed mechanically on the basis of the final IDPAm value.

Nor should LEDAT be understood as a sentencing grid. A high IDPAm score may indicate a higher level of material legal-ecological seriousness within the framework, but it does not determine the appropriate penalty. Sentencing requires additional and individualised legal reasoning concerning culpability, participation, aggravating and mitigating circumstances, proportionality, deterrence, reparation, the statutory penalty framework, and any legally relevant personal or organisational circumstances. LEDAT may make some ecological and legal features more explicit within that analysis, but it cannot replace the court’s sentencing discretion or establish a numerical correspondence between IDPAm and punishment.

The model may also be relevant to ancillary measures such as permit withdrawal, disqualification, compliance obligations, remediation linked to confiscation, corporate environmental monitoring, or obligations to restore affected ecosystems. In those contexts, LEDAT may help identify which dimensions of material seriousness require particular attention and which forms of ecological impact may be relevant to a proportional institutional response. Yet the legal basis, necessity, proportionality, and design of any such measure must be established independently. The index may structure the discussion; it cannot supply the legal authority for the measure.

For these reasons, any use of LEDAT in judicial decisions, prosecutorial files, expert reports, defence submissions, or empirical monitoring should comply with minimum procedural and methodological safeguards. These include a documented coding manual; identification of the factual and evidential source for each variable; a concise justification for every score; explicit identification of missing, insufficient, contested, and non-applicable information; a separate record of each trigger activation; application and explanation of the anti-duplication rule; preservation of an audit trail; and access for the parties to examine and challenge the coding. Where the methodology is used in research, the dataset should additionally preserve coder-specific pre-reconciliation values, reconciliation notes, and formal reliability measures where available.

These safeguards are especially important because the pilot application shows that trigger treatment can affect interpretive-band classification and because conformity-based convictions cannot be treated as equivalent to fully contested adjudication. Black-box use, undisclosed imputation of missing information, or reliance on a final score without access to its coding basis would be inconsistent with the intended legal and methodological character of LEDAT.

The point of LEDAT is not to automate environmental criminal enforcement or to claim that numerical coding can resolve ecological and legal uncertainty. Its more limited purpose is to make selected legal, ecological, and evidential considerations visible in a common structure, so that their factual basis, relative contribution, uncertainty, and points of disagreement can be examined. Used in that way, the framework may support more transparent empirical comparison and more structured legal-ecological argument. Its legitimacy depends on remaining non-binding, evidence-based, contestable, and subject to judicial review, as shown in Table 8.

**Table 8.** Potential uses and limits of LEDAT in enforcement, remedies, sentencing, and empirical monitoring.

Procedural use	LEDAT contribution	Limit
Prosecutorial assessment	Organises ecological and legal features relevant to preliminary threshold analysis and identification of evidential needs	Does not determine whether charges must be filed or whether proof is sufficient
Expert report	Structures legally relevant ecological variables, evidential sources, and uncertainty	Does not replace scientific expertise, expert explanation, or examination of expert evidence
Judicial reasoning	Makes explicit the factors relied upon in an assessment of material legal-ecological seriousness	Does not bind judicial discretion or determine guilt, acquittal, or legal classification
Defence challenge	Enables variable-by-variable examination of factual sources, scores, triggers, and anti-duplication decisions	Does not reverse the burden of proof or alter the presumption of innocence
Remedies	Supports structured discussion of restoration, compensation, remediation, and ecological impact	Does not mechanically determine reparation or provide an independent legal basis for a remedy
Sentencing	May inform explanation of seriousness and proportionality-related ecological features	Is not a sentencing grid and does not determine penalty
Ancillary measures	May assist in identifying ecological factors relevant to permits, disqualification, restoration, compliance, or monitoring	Does not establish necessity, legal authority, or proportionality of the measure
Empirical monitoring	Supports descriptive comparison of cases, sectors, coding profiles, and observed outcomes	Does not establish prevalence, predictive validity, causal effects, or inferential generalisability

## 7. Conclusion

Environmental criminal law increasingly depends on the ability to distinguish administrative unlawfulness from criminally relevant ecological harm or risk. Yet that distinction continues to be mediated through open-textured threshold concepts such as “substantial damage,” “serious harm,” and “serious risk.” Directive (EU) 2024/1203 strengthens the European framework by expanding offences, sanctions, enforcement obligations, and harm-related assessment criteria. It does not, however, remove the practical difficulty of identifying, documenting, and assessing ecological seriousness in individual cases. The central problem is therefore not judicial discretion as such, but the need for transparent and contestable ways of making the legal, ecological, and evidential considerations underlying threshold assessment more observable.

This article has proposed LEDAT as a structured legal-ecological coding framework designed to address that methodological problem. Its contribution lies in organising selected legal and ecological variables within a transparent composite structure capable of supporting empirical comparison and structured threshold reasoning without replacing legal judgment. The Material Environmental Criminal Damage Index (IDPAm) records material legal-ecological seriousness through the integration of a Legal Component, an Ecological Component, and a capped trigger adjustment for qualified ecological features. The Enforceability module (IDPAe) addresses a distinct question: the procedural and evidential viability of sustaining a criminal case. The two dimensions must remain analytically separate. A case may display a materially serious legal-ecological profile while failing because causation, attribution, evidential sufficiency, or procedural validity has not been established; conversely, a procedurally robust case may concern moderate material seriousness.

The pilot application to 36 Spanish first-instance environmental crime judgments issued by Criminal Courts and Provincial Courts acting as trial courts shows that the reconciled LEDAT matrix can be applied to heterogeneous judicial material and can generate a structured descriptive ordering of cases. Under the current coding protocol, IDPAm values range from 0.7 to 9.3, with a mean of 4.18 and a median of 3.70. Nineteen cases fall within the predominantly administrative profile, 13 within the grey area, and 4 within the high material criminal significance band. These figures describe the distribution produced by the framework in the selected pilot corpus. They do not establish the distribution, prevalence, or severity of environmental crime or environmental litigation in Spain more generally, nor do they validate the proposed interpretive bands as independent legal or ecological thresholds.

The descriptive comparison of judicial outcomes reinforces the need for this caution. Convictions display higher mean and median IDPAm values than acquittals in the pilot corpus, but the distributions overlap substantially. The procedural composition of the conviction group also matters: 9 convictions were contested and 11 were conformity-based. The comparison between outcome types is therefore not an external validation test of IDPAm. Judicial outcomes are shaped not only by material seriousness, but also by legal classification, causation, attribution, evidential sufficiency, procedural validity, prosecutorial strategy, and the particular form of case resolution. The pilot can describe the relationship between the reconciled IDPAm profiles and observed outcomes; it cannot establish that IDPAm predicts conviction, explains a particular decision, or substitutes for a separate analysis of enforceability.

The empirical claims advanced here are necessarily limited. The pilot does not establish predictive validity, causal relationships, statistical classification performance, population-level generalisability, statistically calibrated weights, or definitive external validation of the model. It also does not yet establish coder-independent reproducibility of every underlying coding decision. Although the reconciled dataset and scripts permit computational reproduction of the reported calculations and aggregate analyses, the currently available materials do not preserve full coder-specific pre-reconciliation records, systematic source-by-variable evidential references, complete uncertainty codes, or detailed trigger rationales for every case. The limited sample size, filtered jurisprudential selection process, heterogeneous offence sectors, absence of systematic IDPAe coding, and presence of conformity-based convictions require further caution.

The current pilot also identifies specific methodological priorities for the next stage of development. Formal inter-coder reliability measures should be calculated from coder-specific pre-reconciliation data. Future datasets should distinguish documented low scores from missing information, insufficient or contested evidence, and non-applicable variables. Trigger activation should preserve the category concerned, factual and evidential basis, anti-duplication review, raw trigger sum, and capped final value. The preliminary sensitivity analysis should be replicated in larger and more diverse datasets, including alternative weighting structures, trigger specifications, and band boundaries. Further work should also apply LEDAT across additional offence sectors and jurisdictions, distinguish acquittals from contested and conformity-based convictions, and operationalise IDPAe through a separate set of procedural and evidential variables.

The broader contribution of the article is methodological rather than predictive or dispositive. LEDAT does not assume that ecological criminal seriousness can be exhaustively reduced to numerical representation. It proceeds from the narrower premise that structured, transparent, and evidence-based approximation may make selected legal-ecological considerations more visible, comparable, and open to challenge under conditions of ecological and evidential complexity. Properly understood, the framework does not create offences, determine criminal liability, prove causation or culpability, fix penalties, or displace judicial discretion. It provides a means of documenting how legally relevant ecological and normative factors are identified and combined in an empirical analysis of environmental criminal thresholds.

For empirical legal studies, the potential value of LEDAT lies in making the reasoning structures associated with environmental criminal thresholds more observable. The framework can support the coding, comparison, audit, and contestation of selected legal and ecological variables across judicial decisions, provided that its assumptions and limitations remain visible. Within the post-2024 European context, Directive (EU) 2024/1203 reinforces the relevance of specialised capacity, coordination, training, and more comparable environmental-crime data. LEDAT may contribute to empirical research and transparent legal-ecological argument within that context. It is not, however, a harmonised European scoring system, a legally binding threshold, or a substitute for national criminal-law rules, evidential safeguards, adversarial procedure, and judicial assessment.

Its legitimacy depends on remaining non-binding, methodologically restrained, evidence-based, transparent, and open to legal and methodological challenge. Framed in those terms, LEDAT offers not a mechanical solution to environmental criminal thresholds, but a structured framework through which the relationship between legal indeterminacy, ecological complexity, evidential limitations, and empirical observation can be examined more explicitly.

## Code availability statement

The R scripts used to generate the descriptive statistics, figures, consistency checks, and sensitivity analyses are available as Supporting Information. The scripts reproduce the analyses reported in the article from the derived case-level coding dataset. They are provided for transparency, verification, and scholarly replication of the pilot results.

## Ethics statement

This study does not involve interviews, surveys, experiments, animal subjects, or interaction with human participants. The empirical material consists of judicial decisions and derived coding variables. No personal data beyond information contained in judicial decisions was independently collected. The analysis is reported at aggregate and case-coded level, and the supplementary dataset avoids unnecessary personal identifiers. For these reasons, formal ethics committee approval was not required.

## Artificial intelligence and language editing disclosure

The author used DeepL Write/Translator and Perplexity AI (GPT-5.6 Terra) to assist with English-language translation, grammar, and stylistic refinement, as English is not the author’s native language. The tools were not used to generate research data, conduct analysis, produce results, or determine the article’s arguments or conclusions. All AI-assisted language suggestions were reviewed, revised, and approved by the author, who takes full responsibility for the content of the manuscript.

## Data Availability

The coded case-level dataset and all replication materials underlying the analyses reported in this article are openly available in Zenodo under a Creative Commons Attribution 4.0 International licence (CC-BY 4.0): Morelle-Hungría, E. (2026). *Dataset and replication materials: Ecological harm and criminal thresholds – A pilot legal-ecological coding framework for environmental crime judgments (LEDAT, Spain 2020–2025).* Zenodo. https://doi.org/10.5281/zenodo.20524581 The deposit includes the full case-level coding dataset (n = 36) with all legal and ecological variables, derived IDPAm scores, band classifications, judicial outcomes, summary tables underlying all reported statistics and figures, and the R analysis script. The full text of the judgments is not included due to database licensing restrictions (Tirant Prime); all judgments are publicly accessible through official Spanish legal databases. No datasets are under embargo.

## Footnotes

^1^
This article forms part of the research results obtained within the project *“Análisis criminológico de la eficacia de la política criminal medioambiental frente a la emergencia climática – CRIMICLIMA”* (Reference: UJI-2023-02), hosted by the Department of Public Law at Universitat Jaume I and developed during the period 1 January 2024–31 December 2026. This article forms part of the research results obtained within the project ‘Análisis criminológico de la eficacia de la política criminal medioambiental frente a la emergencia climática – CRIMICLIMA’ (Reference: UJI-2023-02), hosted by the Department of Public Law at Universitat Jaume I (1 January 2024–31 December 2026). It also forms part of the project ‘Ocean Crime Narratives: A Polyhedral Assessment of Hegemonic Discourse on Environmental Crime and Harm at Sea (1982-present) – OCN’, funded by the European Commission under Horizon Europe (Grant Agreement No. 101043711), Principal Investigator: Dra. Marta Puxan-Oliva (1 October 2022–30 September 2027). The author participates in the OCN project as a collaborating researcher.
