# Supplementary material for: Ecological harm and criminal thresholds: A pilot legal-ecological coding framework for environmental crime judgments
Source: Open Res Eur. 2026 Sep 17;6:209. Originally published 2026 Jun 17. [Version 2] doi: 10.12688/openreseurope.24170.2 (PMC13612999; doi:10.12688/openreseurope.24170.2)
Supplement: Peer review history

# Reviewer report: Reviewer response for version 2

**DOI:** 10.21956/openreseurope.27254.r81177

**Published:** 2026-09-25

**Authors:** Anna Rita Germani, Demetrio Villani

**Affiliations:** 1 Department of Legal and Economic Studies, University of Rome La Sapienza, Rome, Italy; 2 Dipartimento di Scienze dell'Ambiente e della Prevenzione, University of Ferrara, Ferrara, Italy

We thank the author for the careful and substantial revision of the manuscript. The revised version addresses the principal concerns raised in our previous report. In particular, it clarifies the scope of IDPAm, the operationalisation of the trigger categories, the sample-selection process, and the distinction between contested and conformity-based convictions. The additional sensitivity analysis and the more cautious discussion of the study’s methodological limitations also considerably strengthen the manuscript.

 We have only one remaining point of clarification. Section 4.3 introduces an improved coding protocol distinguishing documented zero values, missing information, insufficient or contested evidence, and non-applicability. However, as acknowledged in Section 5.7, these distinctions were not applied retrospectively to the 36 pilot cases. Section 4.3 should therefore distinguish more explicitly between the protocol proposed for future applications and the calculation actually used in the present pilot.

 Subject to this minor clarification, we have no further substantive comments. The revised manuscript presents the contribution and limitations of the pilot study in an appropriately cautious and transparent manner.

Are sufficient details provided to allow replication of the method development and its use by others?

Partly

Is the description of the method technically sound?

Partly

Are the conclusions about the method and its performance adequately supported by the findings presented in the article?

Partly

Is the rationale for developing the new method (or application) clearly explained

Yes

If any results are presented, are all the source data underlying the results available to ensure full reproducibility?

Partly

Reviewer Expertise:

Environmental law and economics

We confirm that we have read this submission and believe that we have an appropriate level of expertise to confirm that it is of an acceptable scientific standard.

# Reviewer report: Reviewer response for version 2

**DOI:** 10.21956/openreseurope.27254.r81178

**Published:** 2026-09-24

**Authors:** Willy Naresta Hanum

**Affiliations:** 1 Faculty of Law, Universitas Diponegoro, Semarang, Indonesia

The authors have adequately addressed and clarified all of my previous questions and comments. I have no further comments to make on the revised manuscript.

Are sufficient details provided to allow replication of the method development and its use by others?

Partly

Is the description of the method technically sound?

Yes

Are the conclusions about the method and its performance adequately supported by the findings presented in the article?

Partly

Is the rationale for developing the new method (or application) clearly explained

Yes

If any results are presented, are all the source data underlying the results available to ensure full reproducibility?

Yes

Reviewer Expertise:

Constitutional Law; Legislative Drafting; Environmental and Energy Law

I confirm that I have read this submission and believe that I have an appropriate level of expertise to confirm that it is of an acceptable scientific standard.

# Reviewer report: Reviewer response for version 1

**DOI:** 10.21956/openreseurope.26168.r79240

**Published:** 2026-09-04

**Authors:** Willy Naresta Hanum

**Affiliations:** 1 Faculty of Law, Universitas Diponegoro, Semarang, Indonesia

The article presents a novel approach that could be highly useful for assessing and quantifying cases of ecological harm by classifying or systematically measuring the seriousness of ecological harm in the context of criminal thresholds.

 After reviewing the article, I have some questions:

1. We understand that the sample was drawn from a pilot project. However, could the authors provide a clearer explanation of why 36 cases were selected? What considerations or judgment informed the selection of these 36 cases? To what extent is the sample representative? Could the authors provide a more comprehensive explanation of the sample selection criteria? For example, were the cases selected based on similarities in the judgments, similarities in the cases handled, judicial considerations, or other relevant factors?
2. Does the article sufficiently clarify the scope and limitations of the IDPAm so that readers do not interpret the score as a measure of criminal liability or economic damage? Readers might assume that a higher IDPAm score would automatically imply a higher punishment for the offender. However, a high IDPAm score indicates high material seriousness, while evidential enforceability may still be low.
3. Could the authors further clarify whether a higher IDPAm score actually indicates greater “criminal significance,” or whether it primarily indicates greater ecological/material seriousness?
4. The article finds that cases resulting in conviction tend to have higher IDPAm scores than cases resulting in acquittal. However, if LEDAT is intended to help determine or understand criminal thresholds, a high IDPAm score does not necessarily mean that the defendant will automatically be found guilty. How, therefore, is LEDAT intended to be implemented in practice? Does the article provide sufficient justification for this relationship? In particular, could the authors clarify whether IDPAm is primarily intended to measure the material seriousness of ecological harm, rather than to indicate criminal liability or the likelihood of conviction?
5. Could the authors clarify the intended role of LEDAT in judicial decision-making? In particular, to what extent is the IDPAm score intended to inform or assist judges in assessing whether ecological harm reaches a criminal threshold, considering that criminal liability must also be established through the applicable elements of the offence, evidentiary sufficiency, causation, and other relevant legal considerations?

Are sufficient details provided to allow replication of the method development and its use by others?

Partly

Is the description of the method technically sound?

Yes

Are the conclusions about the method and its performance adequately supported by the findings presented in the article?

Partly

Is the rationale for developing the new method (or application) clearly explained

Yes

If any results are presented, are all the source data underlying the results available to ensure full reproducibility?

Yes

Reviewer Expertise:

Constitutional Law; Legislative Drafting; Environmental and Energy Law

I confirm that I have read this submission and believe that I have an appropriate level of expertise to confirm that it is of an acceptable scientific standard, however I have significant reservations, as outlined above.

# Response

**Published:** 2026-09-07

**Authors:** Esteban Morelle-Hungría

**Affiliations:** Public Law, Universitat Jaume I, Castellón de la Plana, Valencian Community, Spain

We thank the reviewer for raising practical questions that were essential to sharpening the legal and methodological boundaries of LEDAT. The revised manuscript (V2) addresses these points as follows.

**1. Practical scope of use in judicial, prosecutorial, and defence practice.** This was the central concern of the report, and it is now addressed comprehensively in Section 6 and Table 8. We have specified, use by use, what LEDAT can and cannot do: in prosecutorial assessment, it may structure preliminary threshold analysis but cannot determine whether charges should be filed; in expert reports, it may organise ecological variables and sources but cannot replace expert judgment; in judicial reasoning, it may make the basis of a material-seriousness assessment explicit but cannot bind judicial discretion or determine guilt; in defence practice, it enables variable-by-variable contradiction but does not reverse the burden of proof. We have also added ancillary measures (permit withdrawal, disqualification, compliance obligations, confiscation-linked remediation) as a distinct row in Table 8, clarifying that LEDAT may help identify relevant ecological dimensions but cannot supply the legal basis, necessity, or proportionality of any such measure.

**2. LEDAT and sentencing.** Section 6 now states explicitly that a high IDPAm score indicates a higher level of material legal-ecological seriousness within the framework but does not determine the appropriate penalty. Sentencing requires additional, individualised legal reasoning on culpability, participation, aggravating and mitigating circumstances, proportionality, and the statutory penalty framework, which LEDAT cannot replace.

**3. Justification for the final sample size (n=36n=36).** Section 5.1 has been rewritten to make clear that 36 was not fixed in advance as a target sample size. It resulted from a documented three-stage filtering process (1,145 records retrieved → 157 first-instance judgments identified → 36 retained after qualitative screening against predefined eligibility criteria). We now specify those eligibility criteria explicitly (procedural stage, substantive connection to Article 325, sufficiency of published information for coding) and state that cases were not selected by outcome, sector, or anticipated IDPAm score.

**4. Legal nature and constitutional safeguards of the model.** Section 4.9 has been substantially expanded to clarify that LEDAT is designed to operate in a manner consistent with legality, minimal intervention, culpability, and proportionality, without claiming to resolve these constitutional questions definitively. We specify that the model does not create offence elements, does not displace the burden or standard of proof, and remains subordinate to judicial assessment in every application.

**5. Risk of "black-box" or mechanical use.** Section 4.7 and Section 6 now require, as a condition of any legitimate use of LEDAT, a documented coding manual, identification of the evidential source for each variable, explicit trigger and anti-duplication documentation, and access for the parties to examine and challenge the coding. We state explicitly that black-box use, undisclosed imputation of missing information, or reliance on a final score without access to its coding basis is incompatible with the intended character of the model. We believe these clarifications directly respond to the reviewer's practical concerns and make the boundaries of legitimate use of LEDAT considerably more explicit than in the original submission.

# Reviewer report: Reviewer response for version 1

**DOI:** 10.21956/openreseurope.26168.r76187

**Published:** 2026-08-18

**Authors:** Angus Nurse

**Affiliations:** 1 Anglia Ruskin University, Chelmsford, England, UK

The paper indicates a potential issue in respect of judicial discretion being exercised in an arbitrary way in environmental crime cases. Central to this problem is the perceived lack of a consistent threshold for assessing issues such as environmental harm. The strength of the paper is in its use of empirical data (a sample of 36 judgments) to apply a model for decision-making that appropriately considers environmental impacts and harms in assessing criminal liability.

 As the paper notes, case datasets are not neutral mirrors but are shaped by a range of factors, including the nature of the cases, the jurisdiction in which they operate, and the behaviours involved in crimes. The paper effectively explains the basics of the LEDAT model although in reading the legal component some aspects of the scoring arguably required further clarification. Sentencing guidelines in some environmental crimes (e.g. wildlife crimes) place some weight on aggravating factors such as deliberate action, use of violence, engagement with organised crime or commercial activity.  The scoring model here appears to 'flatten' these potentially multiple issues into a simple 0-3 matrix with seemingly more weight placed on the species involved (ecological component). This may reflect the model's intended focus on ecological harm and the logic of compatibility with the revised European Framework Directive but in terms of addressing both the criminality involved and the harm involved the paper should clarify this logic, drawing on relevant theoretical perspectives and sentencing literature to clarify its basis.  This is not to negate the value of the model but in terms of applying the model to a wider range of cases and assisting in how to apply the matrix in respect of issues like aggravating factors.

 The paper explains the importance of inter-coder reliability and explains application of the model within its pilot. It notes that the reported coding convergence should be understood as descriptive and provisional and that future applications should report formal agreement measures before reconciliation. The paper makes some suggestions for these but as the paper notes, its pilot application should not be read as a full reliability validation of the model.  It would be helpful for readers to understand clearly what would be required to establish this level of validation. Given the possibility that different coders with different/larger samples might produce different results (or at least not reproduce the same results) that might lead to different conclusions regarding evaluation of seriousness, application of aggravating factors and thus judicial outcomes there is scope to consider the need for trials of different sample sizes, notwithstanding the value of this pilot exercise. The paper helpfully explains and reflects on limitations in the sample size as well as selection bias.  There is a consideration here concerning factors to take into account in jurisdictions where the available case data on environmental criminal litigation might be selectively or inconsistently recorded, or where cases of fully contested convictions may be significant within the dataset.

 These points do not undermine the paper albeit future research suggestions could be developed.

Are sufficient details provided to allow replication of the method development and its use by others?

Partly

Is the description of the method technically sound?

Yes

Are the conclusions about the method and its performance adequately supported by the findings presented in the article?

Yes

Is the rationale for developing the new method (or application) clearly explained

Yes

If any results are presented, are all the source data underlying the results available to ensure full reproducibility?

Partly

Reviewer Expertise:

Environmental law, criminal law, Green Criminology, Wildlife Crime

I confirm that I have read this submission and believe that I have an appropriate level of expertise to confirm that it is of an acceptable scientific standard, however I have significant reservations, as outlined above.

# Response

**Published:** 2026-09-07

**Authors:** Esteban Morelle-Hungría

**Affiliations:** Public Law, Universitat Jaume I, Castellón de la Plana, Valencian Community, Spain

We thank the reviewer for a thorough and constructive report. The revised manuscript (V2) addresses each of the concerns raised, as summarised below.

**1. Judicial datasets as non-neutral mirrors of environmental crime incidence.** This concern has been incorporated explicitly into Section 1 and Section 5.1. We now state clearly that a dataset of judgments records adjudicated and documented cases rather than the full universe of environmental degradation or offending, and that case distributions are shaped by enforcement priorities, inspection capacity, reporting practices, prosecutorial discretion, and unequal institutional resources across sectors and regions. This caveat is repeated at each point where descriptive patterns could otherwise be misread as measures of real-world prevalence or severity (Sections 5.3, 5.4, 5.7).

**2. Selection bias and representativeness of the corpus.** Section 5.1 has been substantially expanded to describe the search and filtering process in full (1,145 records → 157 first-instance judgments → 36 retained after qualitative screening), and to state explicitly that the resulting corpus is a filtered jurisprudential sample dependent on database indexing practices, search terminology, and the availability of written judgments—not a probabilistic sample or census of Spanish environmental criminal litigation. We now state directly that the sample cannot be used to estimate prevalence, detection, or enforcement patterns at the population level.

**3. Sectoral heterogeneity, illustrated by the treatment of noise pollution.** Section 1 (Introduction) and Section 5.4 now discuss this issue directly. Noise-related cases form the largest sectoral group in the pilot corpus but generally occupy the lower range of IDPAm values; we explicitly caution that this pattern should not be read as evidence that noise pollution is inherently less serious or ecologically irrelevant. It reflects the specific evidential, legal, and procedural configuration of the judgments selected, and the broader point that the legal salience of ecological harm is culturally, institutionally, and evidentially mediated—what appears "less serious" in a judicial dataset may instead reflect how harm was detected, framed, and adjudicated.

**4. Relationship between administrative thresholds and criminal-law assessment.** This has been clarified in the discussion of the CONC, SUS, ECO, BIO, and REV variables (Section 4, throughout). Administrative thresholds are now explicitly treated as structured evidential inputs, not as proxies for criminal seriousness in themselves. The coding record must identify the evidential source of any regulatory threshold and the degree of exceedance where reported; where a judgment does not establish this information, that absence is not treated as proof that no ecological feature was present.

**5. Connection to Directive (EU) 2024/1203's statistical and reporting requirements.** We have strengthened this link throughout Sections 4.7, 4.9, and 6, specifying that a case-level record intended to support empirical environmental-crime analysis should preserve not only the final IDPAm score but the underlying Legal and Ecological Component variables, all trigger activations with their anti-duplication assessment, the evidential source for each value, and any coding uncertainty or non-applicability—conditions necessary for the traceability and comparability the Directive anticipates at Union level.

**6. Future research priorities.** Section 5.7 and the Conclusion (Section 7) now specify a concrete research agenda responding to this and other reviewers' observations: expansion of the dataset across time, sectors, and jurisdictions; formal inter-coder reliability reporting from independent pre-reconciliation coding; explicit treatment of missing, contested, and non-applicable information; case-level documentation of trigger and anti-duplication decisions; separation of conformity-based from contested convictions; and systematic incorporation of IDPAe. We are grateful for the reviewer's attention to the risks of treating judicial data as representative of underlying ecological harm, which has materially improved the caution and precision with which the pilot's findings are now framed.

# Reviewer report: Reviewer response for version 1

**DOI:** 10.21956/openreseurope.26168.r76185

**Published:** 2026-08-10

**Authors:** Anna Rita Germani, Demetrio Villani

**Affiliations:** 1 Department of Legal and Economic Studies, University of Rome La Sapienza, Rome, Italy; 2 Dipartimento di Scienze dell'Ambiente e della Prevenzione, University of Ferrara, Ferrara, Italy

**Ecological harm and criminal thresholds: A pilot legal-ecological coding framework for environmental crime judgments**

**Summary and overall assessment**

 The manuscript develops LEDAT (Legal-Ecological Damage Assessment Tool), a structured framework for assessing environmental seriousness in criminal cases. The framework combines a Legal Component and an Ecological Component into a composite Material Environmental Criminal Damage Index (IDPAm), with additional points assigned when particularly serious factors are present. It also distinguishes material seriousness from a separate Enforceability module (IDPAe), although the latter is not implemented in the pilot application.

 LEDAT is applied to 36 Spanish first-instance environmental crime judgments issued between 2020 and 2025. The cases were independently coded by two researchers, who then compared their assessments and agreed on the final coding. The article presents descriptive results, comparisons across judicial outcomes and environmental sectors, and sensitivity analyses based on alternative legal and ecological weights and on the exclusion of the additional seriousness factors.

 The article addresses an important and still insufficiently studied problem. Environmental criminal law relies on concepts such as “substantial damage”, “serious harm”, and “serious risk”, while ecological harm is multidimensional, cumulative, and often difficult to establish on the basis of the available evidence. A transparent framework that makes explicit the factors underlying threshold assessments could therefore be valuable for empirical legal research and, potentially, for supporting prosecutorial, expert, and judicial reasoning.

 The interdisciplinary ambition of the article is particularly valuable. The distinction between material seriousness and procedural enforceability is conceptually promising, and the author appropriately clarifies that LEDAT is not an automated adjudication system, a sentencing grid, or a predictive model. The current pilot study provides a useful first application of the method. Addressing the points below would help clarify some key measurement choices and further strengthen the evidence supporting the transparency, reproducibility, and potential wider application of the framework.

**i) Sections 4.1 - 4.2 (pp. 13 - 15): Clarification of what IDPAm is intended to measure**

 The manuscript makes clear that IDPAm is not a purely ecological measure, but an integrated legal-ecological indicator. This clarification is helpful. However, the interpretation of the index could still be made more explicit. The Legal Component includes offence classification, intent, aggravating circumstances, and recidivism or continuity, while the Ecological Component measures dimensions of ecological harm or risk. As a result, the same ecological harm may receive a different final IDPAm score depending on legal characteristics of the case.

 It would therefore be useful to state even more clearly throughout the paper that IDPAm measures an integrated concept of material criminal seriousness rather than environmental damage alone. This is particularly important given the name “Material Environmental Criminal Damage Index”, which may suggest a more direct measure of ecological damage than the index actually provides. From a legal perspective, the work demonstrates a thorough understanding of the conceptual implications of Directive (EU) 2024/1203 and of the longstanding difficulties associated with the distinction between administrative unlawfulness and criminal relevance. The author successfully situates the proposal within the broader framework of the principles of legality, proportionality, and judicial autonomy, while explicitly acknowledging that the model is intended as a structured interpretative aid rather than a decision-making instrument.

**ii) Section 4.2 and Table 2 (pp. 14 - 16): Measurement properties of the Legal Component**

 From a measurement perspective, the scoring of the legal variables on consecutive 0-3 scales would benefit from further justification. In particular, assigning Articles 325-327 increasing numerical values implies that the categories can be ordered in terms of seriousness and combined with other numerical contributions such as intent, aggravating circumstances, and recidivism.

 I do not seek to assess the doctrinal correctness of this classification. However, the assumptions behind the numerical ordering and aggregation should be explained more clearly. It would also be helpful to clarify how cases involving overlapping provisions, alternative or disputed classifications, or multiple potentially applicable legal categories should be coded. This would make the measurement structure more transparent without implying greater numerical precision than the underlying categories support.

**iii) Sections 4.4 - 4.5 (pp. 17 - 19) and Section 5.6 (pp. 27 - 28): Weighting, interpretive bands, and triggers**

 The paper appropriately presents the 0.4/0.6 legal-ecological weights as provisional and provides useful sensitivity checks using 0.5/0.5 and 0.3/0.7 alternatives. The analysis could be strengthened further by testing whether the results are also sensitive to alternative boundaries for the three interpretive bands. The current thresholds of 0-3.9, 4-6.9, and 7 or above should be clearly presented as provisional categories that have not yet been validated against independent ecological or legal standards.

 From an economic perspective, environmental harm is generally understood as continuous rather than naturally divided into discrete categories. This makes the choice of thresholds particularly important, since relatively small differences in the underlying score may place otherwise similar cases in different bands. Some discussion of this issue would further clarify the interpretation of the proposed categories.

 The trigger system also deserves some additional clarification. Section 4.5 identifies six conceptual trigger categories, while the pilot uses variables T1-T4. Although this difference is acknowledged, the exact mapping is not reported. This is important because removing the triggers changes the band assignment of eight cases and reduces the high-significance group from four cases to one. A clearer explanation of how the six conceptual categories relate to T1-T4, together with case-level information on trigger activation and application of the anti-duplication rule, would make this part of the method easier to evaluate and replicate.

**iv) Sections 4.3 and 4.7 (pp. 15 - 20) and Section 5.7 (pp. 27 - 29): Missing information and comparability across environmental offences**

 The manuscript recognises that the ecological variables may have different relevance across environmental sectors and that explicit rules are needed for incomplete or contested information. However, it is not yet sufficiently clear how these situations are treated in the pilot coding. A value of zero should be clearly distinguished from missing information, insufficient or disputed evidence, and cases in which a variable is not applicable.

 This matters because the 36 judgments cover heterogeneous offences. For example, concentration may be central to a pollution or discharge case but much less meaningful in a case involving protected species or habitats. If missing or non-applicable information is effectively treated as zero, some cases may receive systematically lower scores simply because the relevant judgment contains less ecological information. The coding rules should therefore explain how these different situations affect the Ecological Component and the final IDPAm score.

**v) Sections 4.7 - 4.8 (p. 20), Section 5.2 (pp. 22 - 23), and Section 5.7 (pp. 27 - 29): Inter-coder reliability and coding transparency**

 The manuscript is transparent in acknowledging that formal inter-coder reliability statistics are not yet reported. Since the two researchers coded the cases independently before reconciliation, this pilot would be considerably strengthened if agreement could be calculated from those original pre-reconciliation codings rather than postponed to future applications. Cohen’s kappa or Krippendorff’s alpha could be considered for the coded variables and interpretive bands, with an appropriate agreement measure for LC, EC, and IDPAm. If the original independent records are no longer available, this should simply be stated and the reproducibility claims qualified accordingly.

 Relatedly, the dataset and scripts allow the numerical calculations to be reproduced, but replication of the coding decisions also requires readers to understand why each score was assigned. For each judgment, a clear identifier, a brief explanation of the scores, references to the relevant parts of the judgment, and the reasons for activating any additional seriousness factors would substantially improve transparency. This is particularly important because the manuscript itself presents traceability and reproducibility as central features of LEDAT.

**vi) Sections 5.3 and 5.5 (pp. 24 - 27) and Section 7 (pp. 30 - 31): Interpretation of judicial outcomes and scope of the conclusions**

 The manuscript is appropriately cautious in treating the comparison between IDPAm and judicial outcomes as descriptive. This caution should be maintained consistently, since some variables in the Legal Component are also related to the judicial decision and convictions or acquittals therefore cannot provide an independent test of the index.

 The distinction between contested and conformity-based convictions is also important, given that 11 of the 20 convictions fall into the latter category. If the sample size permits, reporting the score distributions separately for acquittals, contested convictions, and conformity-based convictions would be informative.

 The conclusion already recognises many of the limitations of the pilot. I would only suggest slightly qualifying statements that imply demonstrated improvements in consistency or reproducibility. At this stage, the results show that LEDAT can be applied descriptively to the selected judgments; whether independent coders obtain sufficiently similar results and whether the framework performs consistently across sectors and jurisdictions remain important questions for further testing.

**Minor points**

 A few minor revisions would also improve the manuscript:

- typographical duplications should be corrected, particularly in Section 4.4 (p. 17) and Sections 5.1-5.2 (p. 22);
- some repetition concerning the pilot limitations and the absence of formal reliability statistics could also be reduced, especially across Sections 4.8, 5.2, 5.7 and the Conclusion;
- in Section 4.5 (p. 19), it would be useful to clarify the meaning of “predominantly administrative” in the lowest interpretive band, since the expression may otherwise be understood as referring to an institutional response rather than to the profile generated by the index;
- finally, parts of the broader discussion in Sections 2 and 3 (pp. 4-12) could be shortened slightly to give proportionately more space to the coding rules, measurement choices, and empirical implementation.

Overall, I find the proposed framework original particularly because it makes explicit a set of legal and ecological dimensions that are often assessed less systematically.

 The revisions suggested above are mainly intended to strengthen the transparency of the measurement choices, the reliability of the coding process, and the interpretation of the pilot results.

**Recommended decision: major revisions.**

Are sufficient details provided to allow replication of the method development and its use by others?

Partly

Is the description of the method technically sound?

Partly

Are the conclusions about the method and its performance adequately supported by the findings presented in the article?

Partly

Is the rationale for developing the new method (or application) clearly explained

Yes

If any results are presented, are all the source data underlying the results available to ensure full reproducibility?

Partly

Reviewer Expertise:

Environmental law and economics

We confirm that we have read this submission and believe that we have an appropriate level of expertise to confirm that it is of an acceptable scientific standard, however we have significant reservations, as outlined above.

# Response

**Published:** 2026-09-07

**Authors:** Esteban Morelle-Hungría

**Affiliations:** Public Law, Universitat Jaume I, Castellón de la Plana, Valencian Community, Spain

We thank the reviewer for a detailed and constructive report, which substantially improved the manuscript. Below we summarise how each concern has been addressed in the revised version (V2).

**1. Correspondence between the six conceptual trigger categories and the four operational variables (T1–T4).** This was the most significant methodological gap identified. Section 4.5 and the Appendix A (A9) now provide an explicit mapping: T1 (critically endangered species), T2 (protected area of maximum ecological value), T3 (serious risk to human health), and T4 (extremely hazardous substance). The two remaining conceptual categories—massive/transboundary ecological impact and damage to ecosystem services—are now explicitly identified as *not operationalised* in the present pilot, rather than left ambiguous. Table 3 has been revised accordingly to distinguish operationalised from proposed future trigger categories.

**2. Ambiguity around zero values, missing information, and non-applicability in ecological coding.** Section 4.3 and Section 5.7 now explicitly acknowledge that the current pilot dataset codes EC variables from 0 to 3 without a systematic distinction between a documented low value, missing information, insufficient/contested evidence, and non-applicability. We have not recoded the existing 36 cases retrospectively, since this would require returning to the original judgments; instead, we state this limitation transparently and specify the four-state coding structure (documented / missing / insufficient-contested / non-applicable) that future applications should adopt (Appendix A18–A19).

**3. Treatment of conformity-based convictions as equivalent to contested convictions.** This has been corrected throughout the empirical sections. Section 5.3, Table 6 (renamed in-text as the outcome-type breakdown), Section 5.5, and Appendix A17 now disaggregate judicial outcomes into three categories—acquittals (n=16n=16), contested convictions (n=9n=9), and conformity-based convictions (n=11n=11)—with separate descriptive statistics for each group. Figure 3 has been redesigned to display three outcome categories instead of a binary conviction/acquittal comparison.

**4. Absence of formal inter-coder reliability measures.** Section 4.8 and Section 5.7 now clarify that the reconciled dataset does not preserve complete coder-specific pre-reconciliation values, and therefore that formal coefficients (Cohen's kappa, Krippendorff's alpha, intraclass correlation) cannot be retrospectively calculated. We explicitly recommend these measures for future applications and specify which coefficient is appropriate for which variable type (ordinal vs. continuous/composite outputs).

**5. Sensitivity of the interpretive bands.** Section 5.6 now includes a new sensitivity analysis testing two symmetrical alternative sets of band boundaries (3.5/6.5 and 4.5/7.5) in addition to the previously reported weighting and trigger sensitivity checks. This shows that a small number of borderline cases are sensitive to plausible boundary shifts, reinforcing the heuristic, non-binding status of the bands (Section 4.5).

**6. Sample selection and representativeness.** Section 5.1 has been substantially expanded to clarify that the final sample of 36 judgments resulted from a documented filtering process (1,145 records → 157 first-instance judgments → 36 after qualitative screening), rather than a pre-fixed target size, and that the corpus should be understood as a filtered jurisprudential sample rather than a probabilistic or census-based sample. We believe these revisions directly address the reviewer's concerns while maintaining full transparency about the remaining limitations of the pilot, which are now stated explicitly rather than implied.
